# Navigating Large Chemical Spaces Using Graph Theory and Integer Programming

**DOI:** 10.1021/acs.jcim.6c01810

**Published:** 2026-09-07

**Authors:** Ugochukwu M. Ikegwu, Reid C. Van Lehn, Victor M. Zavala

**Affiliations:** † Department of Chemical and Biological Engineering, 5228 University of Wisconsin—Madison, Madison, Wisconsin 53706, United States; ‡ Department of Chemistry, University of Wisconsin—Madison, Madison, Wisconsin 53706, United States

## Abstract

Navigating and analyzing large chemical spaces are necessary to accelerate the design and discovery of new molecules and chemical processes. In this work, we introduce a computational framework that integrates graph theory and integer programming to enable the efficient navigation of large chemical spaces. Our framework represents the chemical space as a graph, wherein nodes represent molecules and edges represent the degree of similarity or connectivity based on domain-specific information. Using the graph representation, we identify representative molecules by computing the so-called minimum dominating set (MDS), which in our context is the minimum set of molecules that is connected to all other molecules. We present a suite of solution strategies for the MDS problem including heuristic and rigorous integer programming (IP) approaches. We show that these approaches allow us to capture physicochemical properties and domain-specific logic and constraints, facilitating the identification of molecules with the target properties. We demonstrate the effectiveness of the proposed approach by navigating the chemical space of per- and polyfluoroalkyl substances (PFAS); this comprises approximately 15,000 molecular structures. We compare our framework against traditional dimensionality reduction and clustering methods such as t-SNE and K-means clustering.

## Graphical abstract

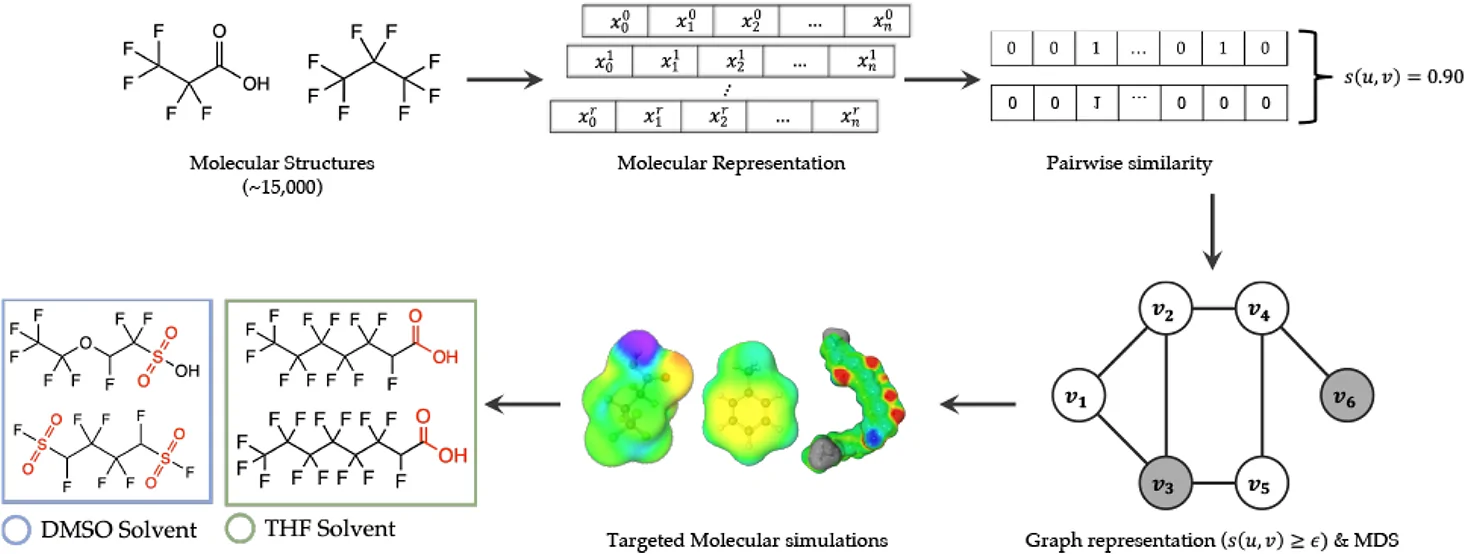

## 1 Introduction

Identifying molecules with target properties is key in areas such as drug discovery, materials design, and solvent design.^1–3^ This requires navigating chemical spaces with potentially thousands to millions of molecules;^4,5^ determining properties for all molecules either by experiments or via molecular simulations is often computationally prohibitive. Such spaces arise across domains, including the exploration of ∼10,000 glycerol-based solvents for plastic recycling,^6^ screening ∼1,000 solvents for selective dissolution in multicomponent plastic waste, yielding combinatorial design spaces on the order of 10,000,^7,8^ virtual screening of ∼120 million ligands for protein binding,^9^ and the screening of solvents for removing ∼17,000 known plastic-associated chemicals.^10,11^

The emergence of cheminformatics and data science approaches, including machine learning, active learning, and advanced statistical approaches, has accelerated the navigation of chemical spaces.^12–15^ In particular, active-learning approaches have been widely adopted across diverse applications. For instance, Qin et al.^2^ applied Bayesian experimental design (BED) to quickly identify green solvent mixtures from a large solvent-mixture design space (i.e., of more than 60,000 unique mixtures) for lignin separation. Khalak et al.^4^ combined active learning with molecular simulations to navigate a library of over 34,000 compounds, quickly identifying high-affinity phosphodiesterase 2 inhibitors in a drug discovery application. Similarly, Chang and colleagues^16^ developed an iterative Gaussian process regression (GPR) method integrated with cheminformatics to explore a design space of more than 300,000 antimicrobial peptides, enabling fast and efficient identification of novel sequences with enhanced antifungal selectivity for therapeutic applications. Hou et al.^17^ introduced a physics-informed active learning protocol to efficiently sample potential energy surfaces, enabling accurate and faster conformer search at a fraction of the cost of pure quantum-chemical calculations. While these data science methods have demonstrated substantial value in exploring large chemical spaces and accelerating decision-making, their performance depends critically on the ability to identify representative molecules that effectively cover the design space.^18–20^

Dimensionality reduction techniques such as principal component analysis (PCA), t-distributed stochastic neighbor embedding (t-SNE), and uniform manifold approximation and projection (UMAP) are powerful methods that have been widely applied to visualize and uncover patterns in large chemical spaces.^15,21–23^ These methods project high-dimensional data representations of molecules into lower-dimensional latent spaces while preserving key structural or molecular information.^24^ Clustering algorithms such as K-means, DBSCAN, and hierarchical clustering further support the identification of molecular families/clusters based on different properties such as structural features and primary functional groups.^25^ Unfortunately, these methods lack the ability to directly encode molecular similarity. As a result, they can struggle to identify a *minimal yet representative set* of unique molecules that covers the chemical space. Identifying such a subset is particularly valuable when aiming to design experiments or targeted simulations to obtain the relevant molecular properties. Specifically, finding representative molecules can help create machine learning models that generalize well to the full space and ensure that the selected molecules capture nonredundant molecular information.^26,27^

In this work, we introduce a computational framework that integrates *graph theory* and *integer programming* (IP) to identify representative molecules in large chemical space. Our framework incorporates a couple of important components: (1) a graph representation of the entire chemical space that captures the degree of similarity between all molecules (nodes are molecules and edges are links that capture similarity) and (2) identification of the minimum representative molecules by computing the dominating sets using heuristic (greedy) and IP approaches.

The concepts of *dominating sets* (DS) and *minimum dominating sets* (MDS) have been extensively studied in graph theory.^28,29^ The DS of a graph is a subset of nodes that is connected to every other node in the graph; in other words, a DS is a representative set of nodes that span the entire graph. The MDS is a DS of the minimum dimension (the smallest possible DS). These concepts are powerful and have been used in many applications such as computer and wireless communication networks,^30^ power systems,^31,32^ and biological networks.^33,34^ For instance, in the context of a power system, an MDS can help identify critical elements in a network that need to be protected to ensure the secure operation of the entire system. In the context of biological and genomic networks, Grady et al.^34^ applied an MDS framework to gene similarity networks to prioritize genes for genome-scale experimental characterization.

An important observation is that the MDS can be computed by solving an IP problem. IP formulations aim to minimize the dimension of the DS while ensuring all nodes are either in the DS or connected to a node in the DS and have been used in several applications.^31,35–37^ To the best of our knowledge, the concepts of DS and MDS have not been applied to chemical sciences. Chemical science also introduces new complexities associated with selecting molecules with specific properties. The proposed framework enables this by leveraging the ability to represent the chemical space in the form of a graph, using notions of molecular similarity. In contrast to prior work in biological contexts,^34^ our proposed framework constructs the graph directly from molecular structural similarity obtained using cheminformatics representations and incorporates molecular properties through node weighting. Thus, formulating the MDS for systematic chemical-space navigation provides a mathematical framework for formalizing the expert-driven identification of structurally representative molecules and enables their selection subject to application-specific similarity and molecular-property criteria. We demonstrate the benefits of the proposed approach via a challenging case study on the analysis of the chemical space of per- and polyfluoroalkyl substances (PFAS).^38^ The U.S. EPA has identified ∼15,000 distinct PFAS structures, and identifying representative molecules can help identify strategies to capture them (e.g., via solvents).

In summary, the contributions of this work are as follows:

- We propose a graph representation of chemical spaces that encodes molecular similarity. Our representation captures similarity in the form of edges and can encode the properties of molecules as node weights. The graph construction is *flexible* and can accommodate different methods to compute the similarity.
- We use the graph representation to formulate variants of the classical MDS problem that leverage a *heuristic approach* and *IP formulations* to compute *approximate* and *exact* solutions, respectively, under varying graph sizes (i.e., different similarity thresholds). This IP approach is generalizable and allows additional constraints to be imposed on the encoded node weight vectors, such as prioritizing representative molecules with the fewest electrons or introducing logic, such as selecting molecules with a specific molecular weight threshold.
- We apply our framework to navigate a combinatorial space of 120 million experiments or molecular simulations (i.e., over 30,000 CPU hours) arising from screening 1,000 solvents for the removal of 15,000 PFAS across several polymer matrices. We further benchmark our approach against traditional dimensionality reduction and clustering techniques, specifically t-SNE and K-means clustering.

## 2 Methods

### 2.1 Graph Representation of Chemical Space

A chemical space can be represented as an undirected graph *G*(*V*,*E*), where the set of nodes *V* corresponds to individual chemical structures (molecules), and the set of edges *E* encodes pairwise similarity. For any node pair (*u* and *v*), an edge exists if molecular similarity is above a certain threshold (*s*(*u*,*v*) ≥ ϵ), where ϵ ∈ [0,1]. When molecules are dissimilar and do not meet the similarity threshold ϵ, the resulting graph is empty, denoted as *G*_empty_ = (*V*, ⌀). Conversely, if every pair of distinct nodes satisfies the threshold, the graph is fully dense and denoted as *G*_full_(*V*, *E*), where *E* = {{*u*,*v*} | *u* ≠ *v*, *u*,*v* ∈ *V*}. Depictions of a full and empty graph are shown in Figure 1a. Intermediate cases reflect chemical spaces that range from chemically diverse to highly similar.

**Figure 1:**
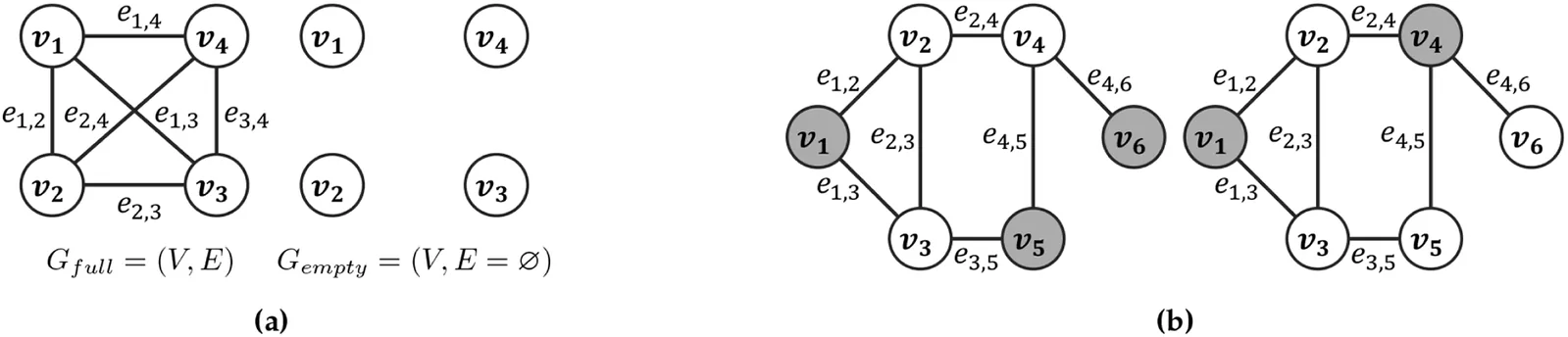
Illustration of (a) a full graph (left) and an empty graph (right) and (b) a dominating set (DS, left) and a minimum dominating set (MDS, right) for the same graph. Nodes (*v_i_*) and edges (*e*_*i,j*_) are represented by circles and lines, respectively, where *i* and *j* denote the end points of an edge, with *e*_*i,j*_ = *e*_*j,i*_. Shaded (gray) nodes indicate members of the DS. In (b), one valid dominating set is $S=\left\{v_{1},v_{5},v_{6}\right\}$ , while the MDS is given by $\mathrm{S̲}=\left\{v_{1},v_{4}\right\}$ . An alternative MDS, $\mathrm{S̲}=\left\{v_{3},v_{4}\right\}$ , also exists, demonstrating the nonuniqueness of MDS solutions. Node *v*_1_ dominates nodes *v*_1_, *v*_2_, and *v*_3_, whereas node *v*_4_ dominates nodes *v*_2_, *v*_4_, *v*_5_, and *v*_6_.

The DS S of a graph is a set of nodes (molecules) that is representative of the entire space. Specifically, for any *G*(*V*,*E*) of a chemical space, the DS S is a subset of (or equal to) the nodes *V* (i.e., S⊆V ) such that every node in *V* is either an element in S or a neighbor to an element in S . A neighbor of node *v* refers to all nodes that are directly connected with *v*. Thus, we can say that for any node *v* ∈ *V*, v∈S holds or there exists a node *u* such that u∈S and (*u*,*v*) ∈ *E* (i.e., *u* and *v* are neighbors). In the latter case, we say that *u* dominates (or represents) *v* and itself.

The DS S with the smallest *cardinality* |S| (i.e., the fewest elements) is referred to as the MDS, which we define as S̲ . Obviously, the MDS can be a subset of or equal to the DS (S̲⁡⊆S) . Figure 1b illustrates the notion of the DS and the MDS. It is also important to note that the MDS might not be unique. For instance, Figure 1b shows that both $\mathrm{S̲}=\left\{v_{1},v_{4}\right\}$ and {*v*_3_, *v*_4_} hold.

### 2.2 Encoding Molecular Similarity

A critical component of the graph representation is the ability to quantify similarity and establish connectivity among molecules. The field of cheminformatics has produced a wide range of approaches for measuring molecular similarity.^39,40^ Depending on the application, molecular connectivity may be defined using diverse molecular representations such as topology, physicochemical properties, functional group or motif overlap, intermolecular interaction behavior, or proximity within a learned latent space. The proposed graph representation is agnostic to the actual approach used to measure the similarity, which is a key advantage of our framework.

While some molecular representations directly provide numerical features, such as physicochemical properties (e.g., molecular weight, partition coefficients, and number of H-bond donors and acceptors), others, particularly those based on molecular structures, require their transformation into numerical representations. For example, molecular structures are commonly encoded using representations such as the Simplified Molecular Input Line Entry System (SMILES) strings^41,42^ or SMILES Arbitrary Target Specification (SMARTS) patterns.^43^ These methods generate fixed-length numerical representations, including binary or count-based fingerprints such as Morgan (extended-connectivity) fingerprints^44,45^ and Molecular ACCess System (MACCS) keys,^46^ which encode the presence or absence of predefined or learned substructural features. In addition to molecular fingerprints, descriptor-based methods such as Mordred^47^ and the Pharmaceutical Data Exploration Laboratory (PaDEL) descriptor framework^48^ compute a wide range of numerical descriptors that capture topological, geometric, and physicochemical characteristics of molecules. These numerical descriptors are then used to compute the molecular similarity among molecules. Because of the ease of computing via the *rdkit* Python library, MACCS keys are adopted as the structural representation for molecules in this work.

Several methods have been developed to compute the similarity between molecular structures, such as the Tanimoto coefficient, cosine coefficient, Tversky coefficient, and Euclidean distance.^49,50^ Figure 2a illustrates examples of the Tanimoto similarity computed using MACCS keys, which is employed in this study as a measure of structural similarity between molecules. This similarity metric captures the ratio of structural feature overlap between molecules and is computed using eq 1, where *X* and *Y* denote the feature vectors of any two molecules. The numerator of eq 1 computes the shared substructures (on-bits) between molecules *X* and *Y*, while |*X*| and |*Y*| represent the number of on-bits in molecules *X* and *Y*, respectively. Although the aforementioned structural similarity methods are implemented differently, they all aim to measure the degree of overlap or proximity between molecules in a feature space that encodes chemical structure or properties. Figure 2b illustrates a practical example of the proposed graph representation using molecular structures. It is important to state that the distance between nodes (i.e., molecules) in the graph representation presented in this work does not reflect the distance or similarity between molecules in a chemical latent space. Rather, the distance or similarity is encoded by the presence or absence of an edge (line) between molecules. As shown in Figure 2b, the imposed similarity threshold ϵ directly influences the topology of the resulting graph. Specifically, for a stricter threshold (ϵ ≥ 0.5), the resulting graph yields a dominating set $S=\left\{v_{1},v_{2},v_{3},v_{7}\right\}$ , whereas a more permissive threshold (ϵ ≥ 0.2) yields a substantially smaller dominating set, $S=\left\{v_{1}\right\}$ .

1 $$T\left(X,Y\right)=\frac{\left|X\cap Y\right|}{\left|X\right|+\left|Y\right|-\left|X\cap Y\right|}$$

**Figure 2:**
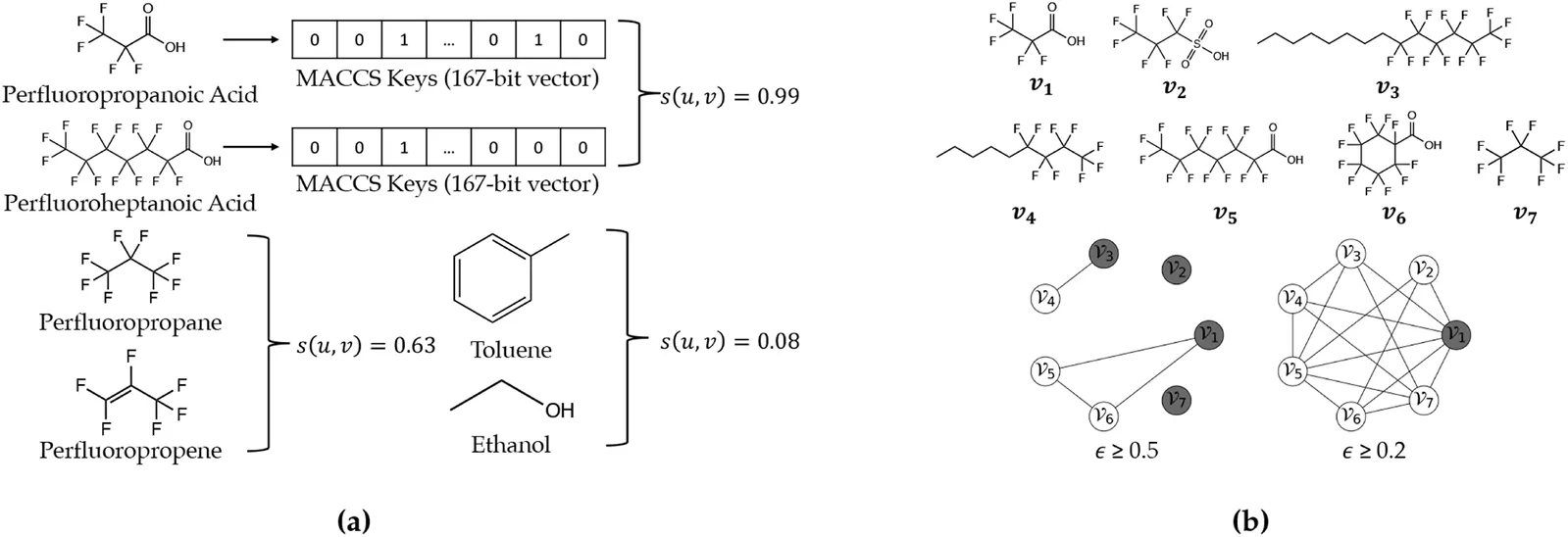
(a) Computed Tanimoto similarity between molecular structures. Each molecule is represented by a 167-bit MACCS fingerprint encoding the presence (1) or absence (0) of predefined structural features. The similarity coefficient *s*(*u,v*) quantifies the structural similarity between molecules *u* and *v*. (b) Effect of the similarity threshold *(*ϵ*)* on the graph representation of a chemical space comprising seven molecules: perfluoropropanoic acid (*v*_1_), perfluoropropanesulfonic acid (*v*_2_), perfluorobutylpentane (*v*_3_), perfluorohexyloctane (*v*_4_), perfluoroheptanoic acid (*v*_5_), perfluorocyclohexanecarboxylic acid (*v*_6_), and perfluoropropane (*v*_7_). Higher similarity thresholds (ϵ ≥ 0.5) result in sparser graphs, whereas lower thresholds (ϵ ≥ 0.2) produce a denser graph. Gray circles indicate nodes included in the dominating set for each graph. *v*_1_, *v*_5_, and *v*_6_ form a family (i.e., a full graph) characterized by their shared carboxylic substructure. Similarly, *v*_3_ and *v*_4_ form a family characterized by their shared perfluoroalkyl and alkyl substructures.

### 2.3 Computing the Minimum Dominating Set (MDS)

#### 2.3.1 Heuristic (Greedy) Approach

Determining the MDS of a graph is an NP-hard problem.^33^ This indicates that, unless P = NP, no polynomial-time method exists that guarantees an exact solution across all graph instances and sizes.^37,51^ Consequently, the computational cost of exact methods grows exponentially with graph size. Substantial effort has been devoted to the development of approximation approaches for solving MDS problems. These approaches aim to provide near-optimal solutions with a tractable computational complexity. Among them, the *greedy approximation method*, hereafter referred to as the *heuristic approach*, is widely used due to its simplicity and scalability, making it particularly suitable for large and dense graphs.^35,52^ Algorithm 1 outlines the implementation of this heuristic approach for solving the DS problem in unweighted graphs; here, *unweighted graphs* denote representations of chemical spaces in which molecular properties are not encoded as node weights.
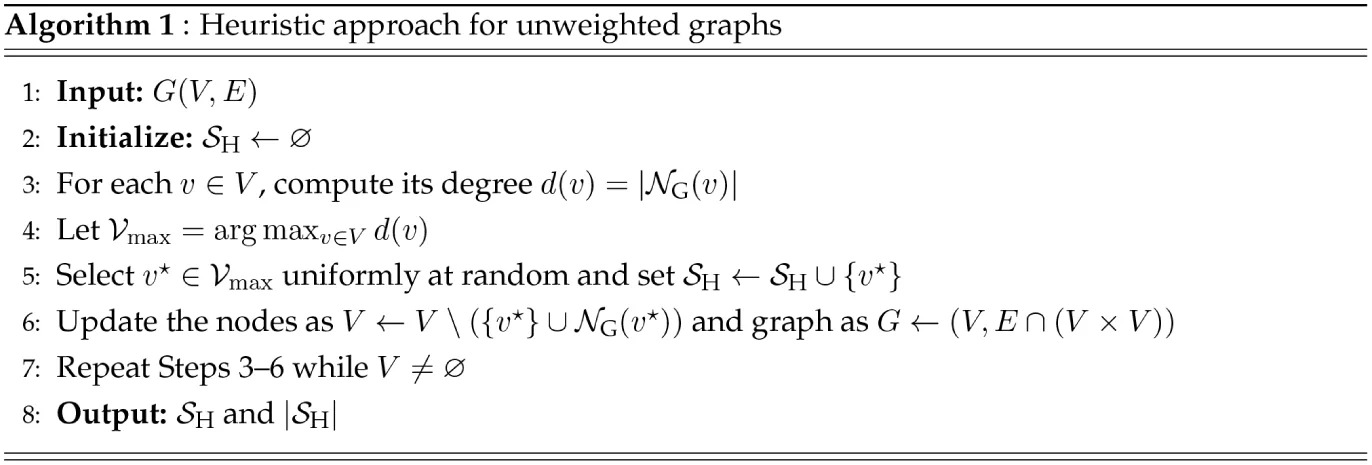


Given an undirected graph *G*(*V*,*E*), as illustrated in Figure 3, we initialize the algorithm by defining an empty dominating set $\left(S_{H}\leftarrow ⌀\right)$ . The degree *d*(*v*) of each node (*v* ∈ *V*) is then computed, where the degree is defined as the cardinality of its neighborhood, $d\left(v\right)=\left|N_{G}\left(v\right)\right|$ . For example, in Figure 3a, node *v*_1_ has degree 4, corresponding to its neighborhood {*v*_1_, *v*_2_, *v*_3_, *v*_4_}; note that a node is considered a neighbor of itself. Next, the set $V_{max}$ is constructed to include all nodes having the maximum degree in the graph. This accounts for cases in which multiple nodes share the same maximum degree. In Figure 3a, nodes *v*_1_, *v*_2_, and *v*_3_ each have degree 4, yielding $V_{\max}=\left\{v_{1},v_{2},v_{3}\right\}$ . One node $v^{*}\in V_{\max}$ is then selected at random and added to the dominating set, i.e., $\left(S_{H}\leftarrow S_{H}\cup \left\{v^{*}\right\}\right)$ . Accordingly, in Figure 3a, selecting v* = *v*_1_ yields $S_{H}=\left\{v_{1}\right\}$ .

**Figure 3:**
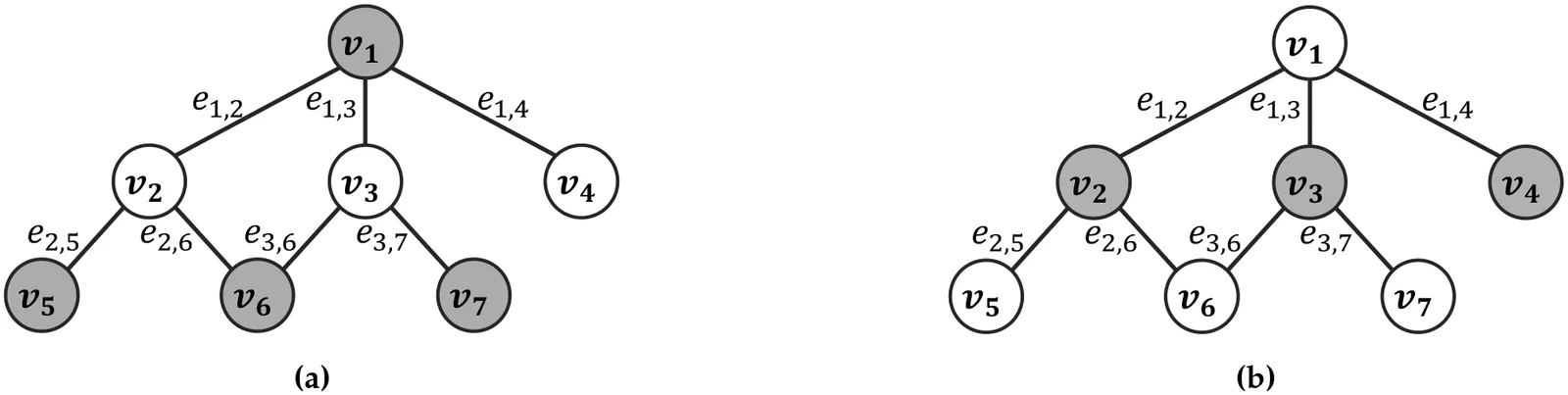
Dominating sets (DS) obtained using the heuristic approach under two different selection rules. Shaded (gray) circles indicate nodes included in the DS. Since nodes *v*_1_, *v*_2_, and *v*_3_ each have degree 4, panel (a) illustrates the DS obtained when v* = *v*_1_ is selected, whereas panel (b) shows the DS obtained when v* = *v*_2_ is selected.

The graph is subsequently updated by removing the node v* and its neighboring nodes $\left(N_{G}\left(v^{*}=v_{1}\right)=v_{2},v_{3},v_{4}\right)$ . Similarly, we keep only edges *E* whose end points are both in the updated graph nodes *V*. In Figure 3a, the remaining nodes are *v*_5_, *v*_6_, and *v*_7_, resulting in the graph *G* = (*V* = {*v*_5_, *v*_6_, *v*_7_}, *E* = ⌀). Note that at this stage, no edges remain among the residual nodes, hence, *E* = ⌀. Repeating steps 3–6 of the algorithm until the graph is empty (*V* = ⌀) yields $S_{H}=\left\{v_{1},v_{5},v_{6},v_{7}\right\}$ with $\left|S_{H}\right|=4$ .

Although this heuristic approach provides a computationally tractable solution to the MDS problem, the resulting DS depends on the choice made in step 5. For instance, as shown in Figure 3b, selecting a different node (e.g., (v* = *v*_2_)) results in $S_{H}=\left\{v_{2},v_{3},v_{4}\right\}$ with $\left|S_{H}\right|=3$ , corresponding to a smaller DS. Such an improvement can translate into significant savings in experimental or computational effort. More importantly, the heuristic approach solution can serve as a useful approximation and an upper bound for the optimal solution to the MDS problem.

#### 2.3.2 Integer Programming Approach

Further efforts have explored alternative approaches and optimization strategies for computing exact or near-optimal solutions to the MDS problem.^35–37^ Among these, mathematical programming has emerged as a widely adopted approach^31,33^ wherein the MDS problem is formulated as an *integer program* (IP). Although the IP formulation remains NP-hard, state-of-the-art mixed-integer linear programming (MILP) solvers such as Gurobi^53^ and cuOpt^54^ can compute high-quality solutions for graph instances with varying sizes within practical times. While the solution to the MDS problem may not always be unique, a key advantage of the IP approach is that MILP solvers are able to provide all possible solutions to the MDS problem of a graph.

2a $$\min\underset{i\in V}{\sum }x_{i}$$

2b $$\mathrm{s.t.}\;x_{j}+\underset{i\in N_{G}\left(j\right)}{\sum }x_{i}\geq 1,\forall j\in V$$

2c $$x_{i}\in \left\{0,1\right\},\forall i\in V$$

Given an undirected graph *G* = (*V*,*E*), we assign an integer variable *x*_*i*_ to each node *i* ∈ *V*, where *x*_*i*_ = 1 indicates that node *i* is included in the DS denoted as $S_{\mathrm{IP}}$ and *x*_*i*_ = 0 otherwise. Note that the aim of the MDS problem is to minimize the cardinality of $\left|S_{\mathrm{IP}}\right|$ while ensuring that every node is either selected or adjacent to at least one selected node. Thus, the IP is given in eq 2. The objective function presented in eq 2 minimizes the sum of all nodes *i* ∈ *V* that are included in the DS to yield the MDS. The constraint presented in eq 3 is referred to as the *domination constraint* and enforces the rule that either a node *j* or its neighbor *i* (i.e., $i\in N_{G}\left(j\right)$ ) must be included in the MDS. This ensures that each molecule, or at least a structurally similar counterpart, is represented in the MDS. In the absence of the domination constraints, the trivial solution *x*_*i*_ = 0 for all *i* would minimize the objective; the constraints are therefore essential to prevent an empty MDS.

3a $$v_{1}:x_{1}+x_{2}+x_{3}+x_{4}\geq 1$$

3b $$v_{2}:x_{2}+x_{1}+x_{5}+x_{6}\geq 1$$

3c $$v_{3}:x_{3}+x_{1}+x_{6}+x_{7}\geq 1$$

3d $$v_{4}:x_{4}+x_{1}\geq 1$$

3e $$v_{5}:x_{5}+x_{2}\geq 1$$

3f $$v_{6}:x_{6}+x_{2}+x_{3}\geq 1$$

3g $$v_{7}:x_{7}+x_{3}\geq 1$$

The number of constraints in the IP formulation scales with the number of nodes in the graph as presented in eq 3, corresponding to the size of the chemical space. For example, considering the graph shown in Figure 3b, the constraint in eq 2 can be explicitly expanded as in eq 3, where the decision variable *x*_*j*_ associated with node *j* is summed with the decision variables corresponding to all nodes adjacent to *j*. Therefore, we observe that the MDS ${\mathrm{S̲}}_{\mathrm{IP}}=\left\{v_{2},v_{3},v_{4}\right\}$ minimizes the objective function in eq 2 and satisfies the constraints outlined in eq 3. Other optimal solutions include ${\mathrm{S̲}}_{\mathrm{IP}}=\left\{v_{1},v_{2},v_{3}\right\}$ and ${\mathrm{S̲}}_{\mathrm{IP}}=\left\{v_{1},v_{3},v_{5}\right\}$ . The overall computational complexity therefore depends on both the number of molecules in the space and the connectivity of the graph, as defined by the edge set *E*.

#### 2.3.3 Hybrid Heuristic–IP Approach

Although the IP approach has been shown to obtain optimal solutions for moderate-sized graphs,^33^ further improvements are required to effectively handle denser graphs. Strategies such as early-stage pruning of the solution space have been widely used to improve the efficiency of large optimization problems.^55,56^ We reformulate the IP constraints presented in eq 2 as eq 4, which we term the *hybrid heuristic–IP* (HIP) approach, and the resulting MDS is denoted as ${\mathrm{S̲}}_{\mathrm{HIP}}$ .

4a $$\min\underset{i\in V}{\sum }x_{i}$$

4b $$\mathrm{s.t.}x_{j}+\underset{i\in N_{G}\left(j\right)}{\sum }x_{i}\geq 1,\forall j\in V$$

4c $$\underset{i\in V}{\sum }x_{i}\leq \left|S_{H}\right|$$

4d $$x_{i}\in \left\{0,1\right\},\forall i\in V$$

While the domination constraints in eq 13 remain unchanged, eq 14 introduces a *bounding constraint* that limits the objective $\left|{\mathrm{S̲}}_{\mathrm{HIP}}\right|$ by the heuristic solution cardinality $\left|S_{H}\right|$ , restricting the feasible region to solutions no worse than the heuristic approach. For instance, looking at Figure 3a with $\left|S_{H}\right|=4$ , this hybrid heuristic–IP approach restricts computational resources to exploring only better solutions. This approach is particularly useful in larger and denser graphs as better solutions to the MDS problem can be attained more efficiently compared to the IP approach.

### 2.4 Computing the Weighted Minimum Dominating Set (MDS)

So far, we have considered graphs without node weights. In the chemical sciences, however, nodes representing molecules are often associated with features such as physicochemical, thermodynamic, economic, and environmental properties. Consequently, beyond identification of the MDS of a chemical space, it may be necessary to prioritize molecules with specific features. For example, when density functional theory (DFT) calculations are required to generate molecular data, prioritizing molecules with fewer electrons is critical, as DFT computational cost scales with electron count.^57,58^ Similarly, when molecular simulations are required to generate data on the selected set of molecules, those with smaller chain lengths or lower molecular weights may be prioritized due to computational limitations.^59^ This capability transforms the MDS framework from a purely structural selection tool into a domain-aware optimization strategy, enabling the targeted and computationally efficient navigation of chemical spaces.

Two main scenarios are considered: (1) An upper or lower bound on a specific molecular feature is known, and (2) no explicit bound is known. For example, in the first scenario, the selection of a representative set can be constrained by a maximum allowable molecular weight due to limitations in molecular modeling or by cost considerations associated with compound procurement for experiments. In this case, the approach imposes a *hard constraint* by explicitly enforcing the prescribed bounds during MDS selection. In the second scenario, when an explicit bound is unspecified or unknown, the approach imposes a *soft constraint* such that the MDS minimizes the feature across the selected molecules. This distinction motivates the formulation of alternative optimization objectives and constraints within the proposed framework. We present the *heuristic* and *IP* approaches for both scenarios to *weighted* graphs, wherein each node *v* ∈ *V* is associated with a feature vector **w**_*v*_ as depicted in Figure 4, hereafter referred to as a weight vector. Although the application of node-weighted graphs is relatively limited in the literature, several studies have explored node-weighted graphs in different applications. For example, Demaine and co-workers^60^ investigated optimization problems on node-weighted networks. Wu and co-workers^61^ employed node-weighted graphs to study structure–activity relationships and activity cliffs in molecular systems. Göke and colleagues^62^ investigated graph transversals, a classical class of graph algorithms in which the objective is to identify a minimum-weight subset of nodes in a node-weighted graph.

**Figure 4:**
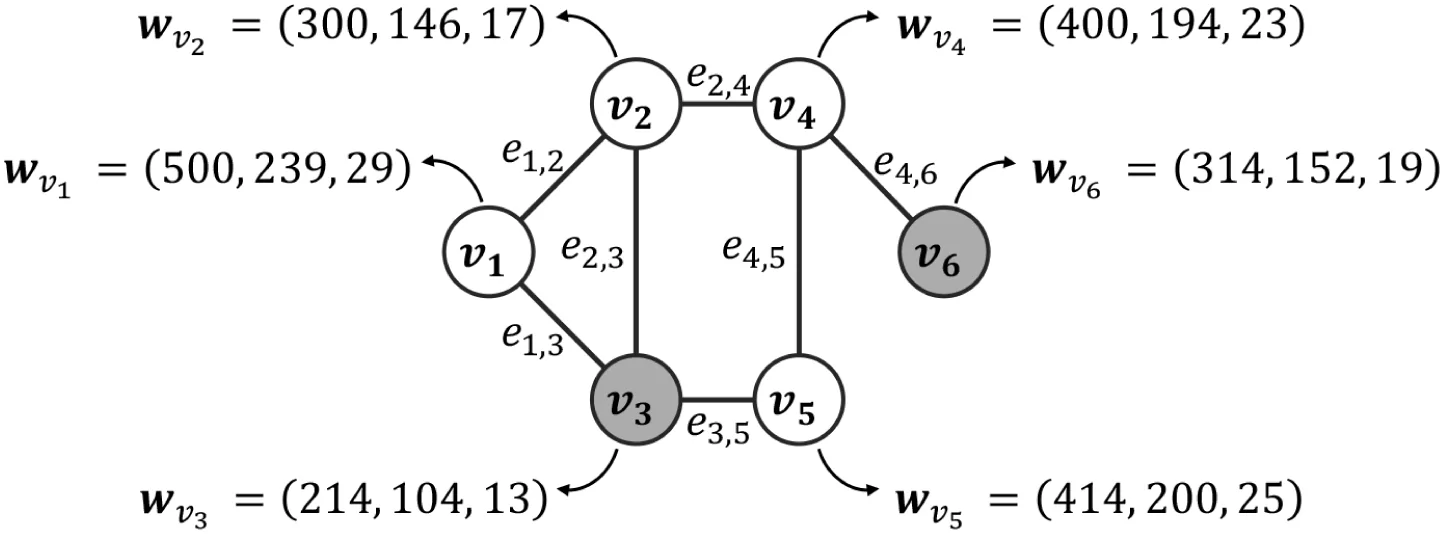
Dominating set (DS) solutions, indicated by shaded nodes, obtained using the weighted heuristic approach with $V_{\theta }^{\left(k\right)}=V_{\theta }^{\left(2\right)}$ . Each node *v* is associated with a weight vector **w** *_v_* encoding node-specific properties (e.g., molecular weight, number of electrons, heavy atom count). Following Algorithm 2, nodes *v*_3_ and *v*_6_ are selected, respectively, as they minimize the second component of the weight vectors across all graph nodes.

#### 2.4.1 Scenario I: Known Molecular Feature Bounds

##### 2.4.1.1 Weighted Heuristic Approach

In the unweighted heuristic approach, the node v* selected in step 5 of Algorithm 1 is chosen at random. However, in this approach wherein nodes *v* ∈ *V* of graph *G*(*V*,*E*) are associated with weight vectors **w**_*v*_ and a desired bound *(θ)* of a weight vector component *w*_*v*,*k*_ is specified, this step is modified such that v* satisfies the logical constraint with respect to the particular weight vector component(s) *k*. Thus, Algorithm 2 outlines the implementation of the heuristic approach for solving the DS problem in weighted graphs with known bounds.
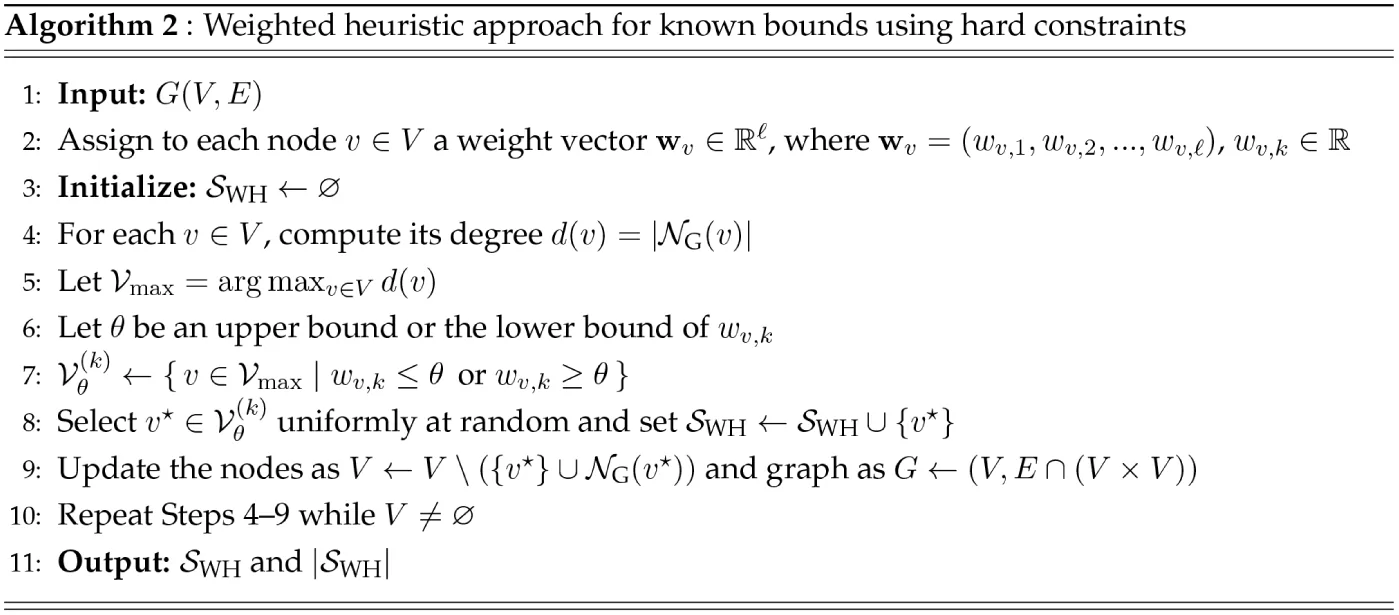


Compared to Algorithm 1, Algorithm 2 includes three additional steps (steps 2, 6, and 7) and a modification to step 8. In step 2, we assign weight vectors **w**_*v*_ to each node *v* ∈ *V*, where **w**_*v*_ is a real space with l− dimensions and *w*_*v*,*k*_ denotes the *k* ^*th*^ element of the weight vector of node *v*. For example, as shown in Figure 4, the 1^*st*^, 2^*nd*^, and 3^*rd*^ elements of each **w**_*v*_ correspond to their molecular weight, number of electrons, and heavy atom count, respectively.

After computing the set of nodes with the maximum degree in step 5, the known bound to the molecular feature of interest is assigned *θ* in step 6. For example, in Figure 4, the set of nodes with the maximum degree is given by $V_{\max}=\left\{v_{2},v_{3},v_{4}\right\}$ , where each node $v\in V_{\max}$ has degree 4. Suppose the objective is to select a representative set of molecules with molecular weight not exceeding 350 g/mol. In this case, the molecular feature of interest corresponds to the first component of the weight vector, i.e., *w*_*v,k*_ = *w*_*v*,1_, and the threshold is θ = 350 g/mol.

In step 7 of Algorithm 2, all nodes $v\in V_{\max}$ that satisfy the imposed constraint on the molecular feature are assigned to $V_{\theta }^{\left(k\right)}$ . Accordingly, for Figure 4, this yields $V_{\theta =350}^{\left(1\right)}=\left\{v_{2},v_{3}\right\}$ . A node v* is then selected at random from this set and added to the dominating set, i.e., $S_{\mathrm{WH}}\leftarrow S_{\mathrm{WH}}\cup \left\{v^{*}\right\}$ . For the illustrated example, this results in $S_{\mathrm{WH}}=\left\{v_{3}\right\}$ . The graph is subsequently updated as described in step 9 of Algorithm 2, and steps 4–9 of Algorithm 2 are repeated until the graph is empty, i.e., *V* ≠ ⌀. Accordingly, the resulting dominating set for the graph presented in Figure 4 is given by $S_{\mathrm{WH}}=\left\{v_{3},v_{6}\right\}$ .

##### 2.4.1.2 Weighted Hybrid Heuristic–IP Approach

A couple of IP approaches are presented to incorporate hard constraints on the molecular features. The first approach integrates a *logical constraint* within the IP formulation as introduced by Raman and Grossmann.^63^ The second approach considers a *preselection approach* where feasible molecules that satisfy the logical constraints are identified, before then applying the IP formulation presented in eq 4. Despite their implementation differences, both approaches identify the MDS exclusively from feasible molecules that satisfy the imposed feature constraints. Details of the *preselection approach* are provided in Text S1 of the Supporting Information (SI).

The incorporation of logical constraints in mixed-integer programming (MIP) formulations is well-established, with common methodologies including the Big-M method and indicator constraints.^63–66^ In the logical constraint approach, we employ *indicator constraints* to maintain numerical stability and avoid the potential pitfalls of large constants associated with the Big-M method.^65,66^ Consequently, the IP is reformulated as shown in eq 5. Here, unlike the unweighted IP formulation in eq 2, we enforce feasibility with respect to molecular feature *k* by introducing a *logical constraint* in eq 18 that prevents the selection of nodes whose molecular feature exceeds a prescribed threshold. This logical constraint approach directly restricts the feasible decision space based on known molecular properties during screening.

5a $$\min\underset{i\in V}{\sum }x_{i}$$

5b $$\mathrm{s.t.}x_{j}+\underset{i\in N_{G}\left(j\right)}{\sum }x_{i}\geq 1,\forall j\in V$$

5c $$x_{i}\leq I\left(w_{i,k}\leq \theta \right),\forall i\in V$$

5d $$x_{i}\in \left\{0,1\right\},\forall i\in V$$

The objective function, domination constraint, and variables in eq 5 retain their meanings as presented in eq 2. The introduced indicator constraint in eq 18 is expanded as eq 20, where I(·) is a known constant that reflects whether the logical constraint on each node *i* with respect to the molecular property *k* is satisfied or otherwise, and *θ* is the known bound on the molecular property. We note that the constant I(·) is typically computed before solving the IP.

6 $$I\left(w_{i,k}\leq \theta \right)=\left\{ \begin{array}{ll} 1, & \mathrm{if}w_{i,k}\leq \theta ,\\ 0, & \mathrm{otherwise.} \end{array}\right.$$

For example, adopting the graph presented in Figure 4, suppose we aim to identify the MDS containing only molecules whose molecular weights do not exceed 350 g/mol, i.e., *w*_*i*,*k*_ = *w*_*i*,1_ and *θ* = 350 g/mol, which implies that $I\left(w_{1,1}\leq \theta \right)=I\left(w_{4,1}\leq \theta \right)=I\left(w_{5,1}\leq \theta \right)=0$ while $I\left(w_{2,1}\leq \theta \right)=I\left(w_{3,1}\leq \theta \right)=I\left(w_{6,1}\leq \theta \right)=1$ Therefore, the resulting indicator constraint can be expanded as *x*_1_ ≤ 0, *x*_2_ ≤ 1, *x*_3_ ≤ 1, *x*_4_ ≤ 0, *x*_5_ ≤ 0, *x*_6_ ≤ 1. However, since *x*_*i*_ ∈ {0,1}, therefore *x*_1_ = *x*_4_ = *x*_5_ = 0. Lastly, we can observe that *x*_3_ = *x*_6_ = 1 satisfies all constraints while minimizing the objective function presented in eq 5, resulting in the MDS as *S̲*_WIP_ = {*v*_3_, *v*_6_}. In the case where the upper and lower bounds on a molecular property are known and wish to be implemented, the indicator constraint presented in eq 5 is modified as $x_{i}\leq I\left(\mathrm{\theta ̲}\leq w_{i,k}\leq \mathrm{\theta ̅}\right),\forall i\in V$ where *θ̲* and *θ̅* are the lower and upper bounds, respectively.

A limitation of this hard constraint approach is that some molecules may remain nondominated if isolated molecules fail to satisfy the imposed feature constraints (see Text S2 of the Supporting Information). Moreover, this formulation requires explicit feature bounds, which may be unavailable in practice, and overly restrictive bounds can render the MDS problem to be infeasible.

#### 2.4.2 Scenario II: Unknown Molecular Feature Bounds

Rather than enforcing a strict bound on the molecular feature, here, we employ a soft constraint that aims at identifying representative sets of molecules that minimize the aggregate value of the molecular property *k*. This approach ensures that the MDS fully spans the entire chemical space. We also present both the heuristic and hybrid heuristic–IP approaches.

##### 2.4.2.1 Weighted Heuristic Approach

Unlike in the heuristic approach with known feature bound(s), here, v* is selected from $v\in V_{min}^{\left(k\right)}$ wherein the weight component *w*_*v*,*k*_ ∈ w_*v*_ is minimized across all nodes. Thus, Algorithm 3 outlines the implementation of the heuristic approach for solving the DS problem in weighted graphs with unknown bounds.
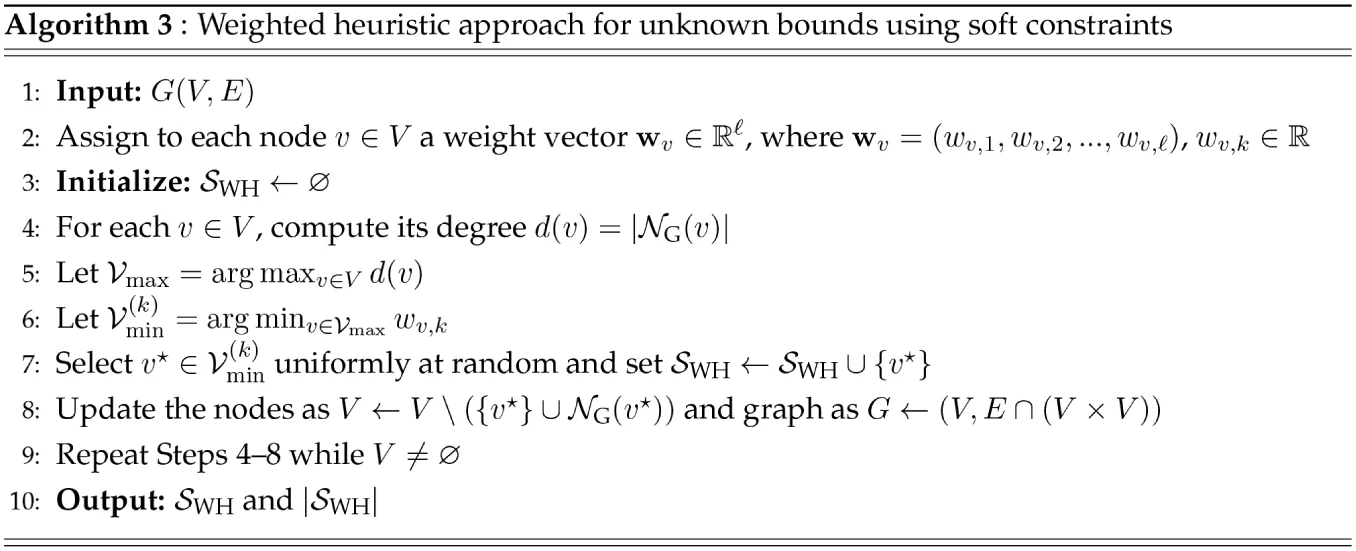


Compared to Algorithm 1, Algorithm 3 includes two additional steps (steps 2 and 6), and step 7 was modified in comparison. Similar to Algorithm 2, in step 2, we assign weight vectors **w**_*v*_ to each node *v* ∈ *V*. After computing the set of nodes with the maximum degree in step 5, step 6 determines the subset of nodes, denoted by $V_{\min}^{\left(k\right)}$ , whose *k* ^*th*^ weight component *w*_*v*,*k*_ is minimal across all nodes $v\in V_{max}$ , thus, $V_{\min}^{\left(k\right)}\subseteq V_{max}$ . Subsequently, v* is selected at random from $V_{\min}^{\left(k\right)}$ and updated to $S_{\mathrm{WH}}$ in step 7. From Figure 4, nodes *v*_2_, *v*_3_, and *v*_4_ exhibit the maximum degree of four, therefore, $V_{max}=\left\{v_{2},v_{3},v_{4}\right\}$ . If the selection criterion prioritizes minimizing the number of electrons (i.e., the *k* ^th^ = second weight component), then $V_{\min}^{\left(2\right)}=\left\{v_{3}\right\}$ , as *v*_3_ has the lowest electron count $\left(w_{v_{3,2}}=104\right)$ . The remaining steps proceed analogously to the unweighted heuristic approach, yielding $S_{\mathrm{WH}}=\left\{v_{3},v_{6}\right\}$ .

##### 2.4.2.2 Weighted Hybrid Heuristic–IP Approach

In this approach, the IP is reformulated to incorporate node-weight vectors and simultaneously minimize the molecular property across all selected molecules contained in the MDS. Specifically, *w*_*v*,*k*_ is incorporated into the objective function, as shown in eq 21. All variables in eq 21 retain their meaning as presented in previous formulations.

7 $$\begin{array}{ll} \min & \underset{i\in V}{\sum }w_{v,k}\cdot x_{i}\\ \mathrm{s.t.} & x_{j}+\underset{i\in N_{G}\left(j\right)}{\sum }x_{i}\geq 1,\forall j\in V\\ & \underset{i\in V}{\sum }x_{i}\leq \left|S_{\mathrm{WH}}\right|\\ & x_{i}\in \left\{0,1\right\},\forall i\in V \end{array}$$

A soft objective is formulated in eq 21, implicitly allowing any molecule to be selected but penalizing those whose molecular property *k* has high magnitude. Minimizing this objective function simultaneously reduces the size of the DS and the aggregate value of the selected weight component across all nodes *v* ∈ *V*. For example, adopting Figure 4, the aim of identifying the MDS with the least aggregate value of the number of electrons (i.e., *w*_*v*,*k*_ = *w*_*v*,2_) across all molecules will lead to minimizing the function *S̲*_WHIP_ = 239 · *v*_1_ + 146 · *v*_2_ + 104 · *v*_3_ + 194 · *v*_4_ + 200 · *v*_5_ + 152 · *v*_6_. We can observe that all solutions to the MDS problem include {*v*_1_,*v*_4_}, {*v*_1_,*v*_6_}, {*v*_2_,*v*_4_}, {*v*_3_,*v*_4_}, and {*v*_3_,*v*_6_}. Consequently, substituting the decision variables *x*_*i*_ into the objective function (i.e., *x*_*i*_ = 1 if $v_{i}\in \mathrm{S̲}$ ) for the different solutions S̲ will lead to {*v*_1_,*v*_4_} = 433, {*v*_1_,*v*_6_} = 391, {*v*_2_,*v*_4_} = 340, {*v*_3_,*v*_4_} = 298, and {*v*_3_,*v*_6_} = 256. Therefore, the MDS solution that minimizes the aggregate value of the number of electrons across all molecules and satisfies all constraints is given as ${\mathrm{S̲}}_{\mathrm{WHIP}}=\left\{v_{3},v_{6}\right\}=256$ .

### 2.5 Computing the Minimum Independent Dominating Set (MIDS)

Here, the framework is extended by imposing constraints on the connectivity of molecules within the MDS rather than on molecular features, further demonstrating its flexibility as a robust chemical-space navigation tool. For example, considering the unweighted graphs represented in Figure 5, several MDS solutions exist including {*v*_1_,*v*_3_}, {*v*_1_,*v*_4_}, {*v*_1_,*v*_5_}, {*v*_2_,*v*_3_}, {*v*_2_,*v*_4_}, {*v*_2_,*v*_5_}, and {*v*_3_,*v*_5_}. However, solutions such as {*v*_1_,*v*_5_}, {*v*_2_,*v*_3_}, and {*v*_3_,*v*_5_} do not contain molecules that are diverse because the two molecules contained in these MDS solutions are connected, i.e., somewhat similar. Ensuring molecular diversity is particularly valuable within an active learning framework, where training predictive models on a diverse and representative set of molecules is essential for improving exploration and the overall model accuracy.^4,67^ The MDS variant that thus imposes restraints on connectivity is called the *minimum independent dominating set* (MIDS).

**Figure 5:**
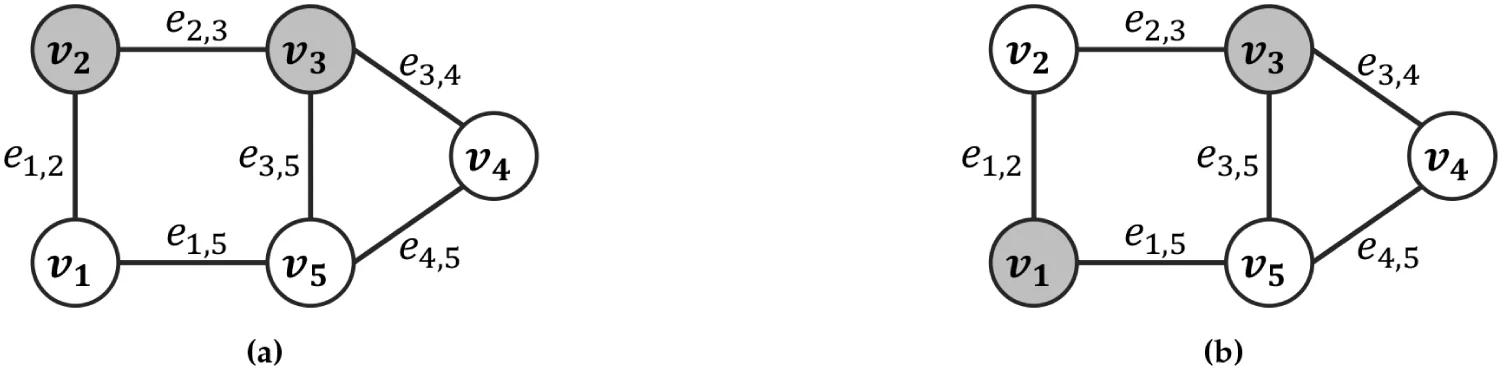
Minimum dominating sets (MDS) under two different formulations: (a) without logical constraints, where the selected molecules may not be independent (i.e., can be connected) and (b) with a logical constraint, where the selected molecules in the MDS must be independent, i.e., the minimum independent dominating set (MIDS) should be as diverse as possible.

A close look at the heuristic approach presented in this work, i.e., Algorithm 1, Algorithm 2, and Algorithm 3, reveals that these inherently yield the MIDS of a given chemical space. For example, applying Algorithm 1 to Figure 5a will yield Figure 5b if v* = *v*_3_ (see step 5 of Algorithm 1). This solution is more diverse than the solution presented in Figure 5a, where $\mathrm{S̲}=\left\{v_{2},v_{3}\right\}$ which may be obtained via the IP approach. Therefore, we reformulate the IP presented in eq 4 such that the molecules in the MDS are independent (unique), leading to eq 8. While the objective function and the constraints retain their meaning as described in eq 4, a new constraint, which we refer to as the *independent domination constraint*, is introduced in eq 24. This constraint enforces that only one node in a neighborhood can belong to the MDS. An example showing the detailed implementation of this IP formulation in Figure 5 is presented in Text S3 of the Supporting Information.

8a $$\min\underset{i\in V}{\sum }x_{i}$$

8b $$\mathrm{s.t.}x_{j}+\underset{i\in N_{G}\left(j\right)}{\sum }x_{i}\geq 1,\;\forall j\in V$$

8c $$x_{i}+x_{j}\leq 1,\forall \left(i,j\right)\in E$$

8d $$\underset{i\in V}{\sum }x_{i}\leq \left|S_{H}\right|$$

8e $$x_{i}\in \left\{0,1\right\},\forall i\in V$$

Lastly, although several solutions may still exist for the unweighted MIDS, the objective function presented in the IP can be reformulated to ∑_*i∈V*_ *w*_*v*,*k*_ · *x*_*i*_ such that the solution that minimizes a specific molecular feature (*w*_*v*,*k*_) across the molecules in MIDS is selected as the optimal solution, thus showing the adaptability of the IP approach of solving the MDS problem particularly for molecular science applications.

In the following sections, we apply the proposed framework to a challenging chemical engineering problem: the removal of PFASs from plastic waste using a solvent. This case study integrates molecular simulations with the developed framework to efficiently navigate the large PFAS space by identifying the MDS, thereby enabling a faster and more informed solvent selection for PFAS removal.

## 3 Case Study on Per- and Polyfluoroalkyl Substances (PFAS)

PFAS have attracted regulatory and scientific attention due to their environmental persistence and health hazards.^68–70^ Consequently, the development of effective strategies for PFAS removal has become critical. Here, we focus on PFAS removal from postconsumer plastic waste, which is necessary to ensure high-quality, contaminant-free recycled plastics.^11,71^ PFAS may be found in plastic waste as intentionally added polymeric species in plastic products to impart water-, oil-, and stain-resistant properties;^72,73^ small-molecule PFAS have also been detected in plastic waste formed unintentionally during the direct fluorination of polymers^74^ or due to migration from other sources. Despite their desirable hydrophobic and oleophobic characteristics, PFAS exhibit extreme environmental persistence and bioaccumulation, posing significant human health risks, including endocrine-disrupting effects.^75–77^ Examples of these PFAS are presented in Figure 2b and in Figure S2 of the Supporting Information and have been detected in fossil-based and plant-based plastic wastes.^78,79^

Solvent-based recycling has emerged as a promising approach for removing contaminants like PFAS from plastic waste.^11^ The removal of small-molecule PFAS from plastic waste using solvent-based approaches requires the identification of solvents that favorably partition PFAS from the polymer matrix into the solvent phase, as demonstrated by Zhou and colleagues.^11^ This solvent selection process is governed by PFAS solubilities and their distribution coefficients (log *D*) across polymer–solvent systems, which can be predicted via molecular simulations.^11^ However, the combinatorial complexity arising from the 14,735 EPA-identified PFAS structures,^38^ eight common polymers (e.g., polyethylene (PE), ethylene vinyl alcohol (EVOH), polypropylene (PP), etc.), and approximately 1,000 candidate solvents^59^ yields hundreds of millions of possible experiments or molecular simulations (e.g., 14,735 × 8 × 1000 = 117,880,000). While experimental screening is impractical, exclusive reliance on high-fidelity molecular simulations (e.g., density functional theory (DFT)) is also computationally prohibitive, requiring years of compute time (i.e., ∼30,000 CPU-hours). Therefore, to efficiently navigate this large chemical space, we apply our framework to identify the MDS of the PFAS space based on structural similarity, enabling targeted molecular simulations and highlighting that structurally similar compounds exhibit comparable molecular properties.^11,80,81^ In other words, using our framework, we aim to identify the smallest set of representative parent PFAS structures such that identifying solvents capable of removing these parent structures would imply effective removal of all structurally connected (similar) PFAS, thereby significantly reducing the number of required simulations. We also identify dominant substructures and functional groups within the PFAS chemical space, as well as overlapping structures among parent groups, which we term *bridging molecules*, a key advantage over existing data science approaches.

We adopt the PFAS structural database, PFASSTRUCTv5,^82^ curated by the U.S. Environmental Protection Agency (EPA) and last updated in August 2022, which contains 14,735 PFAS structures for our analyses. A detailed description of the data curation and classification procedures is provided in Text S4 of the Supporting Information. Following database cleaning and preprocessing, 14,722 valid PFAS structures remained for further analysis. Additional details on the structural classification of the database are presented in Figure S6 and Text S5 of the Supporting Information.

### 3.1 Molecular Simulations and Solvent Screening Analysis

As expounded by Zhou and colleagues,^11^ the removal of PFAS contaminants from a polymer is guided by the distribution coefficient (log *D*) across polymer–solvent systems and is given by eq 27, where [*c*]^*s*^ and [*c*]^*p*^ are the concentration of contaminant *c* in the solvent *s* and polymer *p*, $\mu_{c}^{p}$ and $\mu_{c}^{s}$ are the infinite-dilution chemical potential of the contaminant *c* in the pure polymer (represented as an effective *liquid* based on the assumption that contaminants can only partition into amorphous, liquid-like regions of the polymer phase) and solvent phase, respectively, *V* ^*p*^ and *V* ^*s*^ are the molar volumes of the polymer and solvent phases, and *α* is the correction term that accounts for the degree of ionization of the contaminant in the aqueous phase. This correction term *α* may be of the form log­(1 + 10^*pH* –^ *^pKa^*) for anions, log­(1 + 10^*pKa* –^ *^pH^*) for cations, or a more complex sum of multiple ionized states with multiple *pK*_*a*_ for amphoteric structures. For neutral compounds, the correction term α is negligible.

9 $$\log D=\log\frac{{[c]}^{s}}{{[c]}^{p}}=\frac{\mu_{c}^{p}-\mu_{c}^{s}}{RT\;\ln10}+\log\left(\frac{V^{p}}{V^{s}}\right)-\alpha$$

The distribution coefficient quantifies the equilibrium partitioning of a contaminant between the solvent and polymer phases. A positive distribution coefficient (log *D* > 0) indicates that, at equilibrium, the contaminant preferentially partitions into the solvent phase and is therefore likely to migrate from the polymer into the solvent. Conversely, a negative distribution coefficient (log *D* < 0) indicates preferential retention within the polymer phase. While the solubility of the contaminant in the selected solvent is also important, this work focuses on the log *D* value. Moreover, the log *D* value is often reflective of the solubility of the component.

In this study, we employ the conductor-like screening model for real solvents (COSMO-RS) to predict log *D* values of PFAS compounds.^83^ Although other equation-of-state-based and quantum-mechanical-based approaches, such as UNIversal quasichemical Functional-group Activity Coefficients (UNIFAC)^84^ Solvation Model based on Density (SMD)^85^ and openCOSMO-RS^86^ are available, COSMO-RS offers distinct advantages for analyzing structurally complex molecules. Specifically, it requires only DFT calculations as input and incorporates fine-tuned interaction parameters that enable the prediction of a wide range of thermodynamic properties, including log *D*.^87–89^ All predicted log *D* values generated in this work are available in the SI.

A multistep workflow is adopted to predict log *D* values of PFAS contaminants in several polymer–solvent systems and is depicted in Figure 6. First, each PFAS 3D structure is built and optimized using the Avogadro software version 1.2.0. This step produces a single PFAS conformer corresponding to a minimum-energy structure and is converted to a Gaussian input file for DFT calculation. This step includes a geometry optimization in implicit water using the conductor-like polarizable continuum model and a single-point calculation in the infinite dielectric constant limit and is performed with Gaussian 16 at the BVP86/TZVP/DGA1 level of theory.^90^ These DFT calculations provide the necessary COSMO files containing screening charge densities and corresponding σ-profiles needed for the COSMO-RS calculations. The screening charge densities for the solvents used in the study were precomputed and are contained in the COSMObase-1901-BP-TZVP database. Similarly, the screening charge densities for the polymers employed in this study were obtained from our previous studies.^59,91^ Unlike the small-molecule PFAS structures, molecular dynamics simulations are performed to generate the different polymer conformers in solvent, after which DFT calculations are performed on the conformers to generate the screening charge densities and corresponding σ-profiles needed for the COSMO-RS calculations. A summary of the molecular simulation workflow for generating the polymer screening charge densities adapted from the aforementioned studies is presented in Text S6 of the SI.

**Figure 6:**
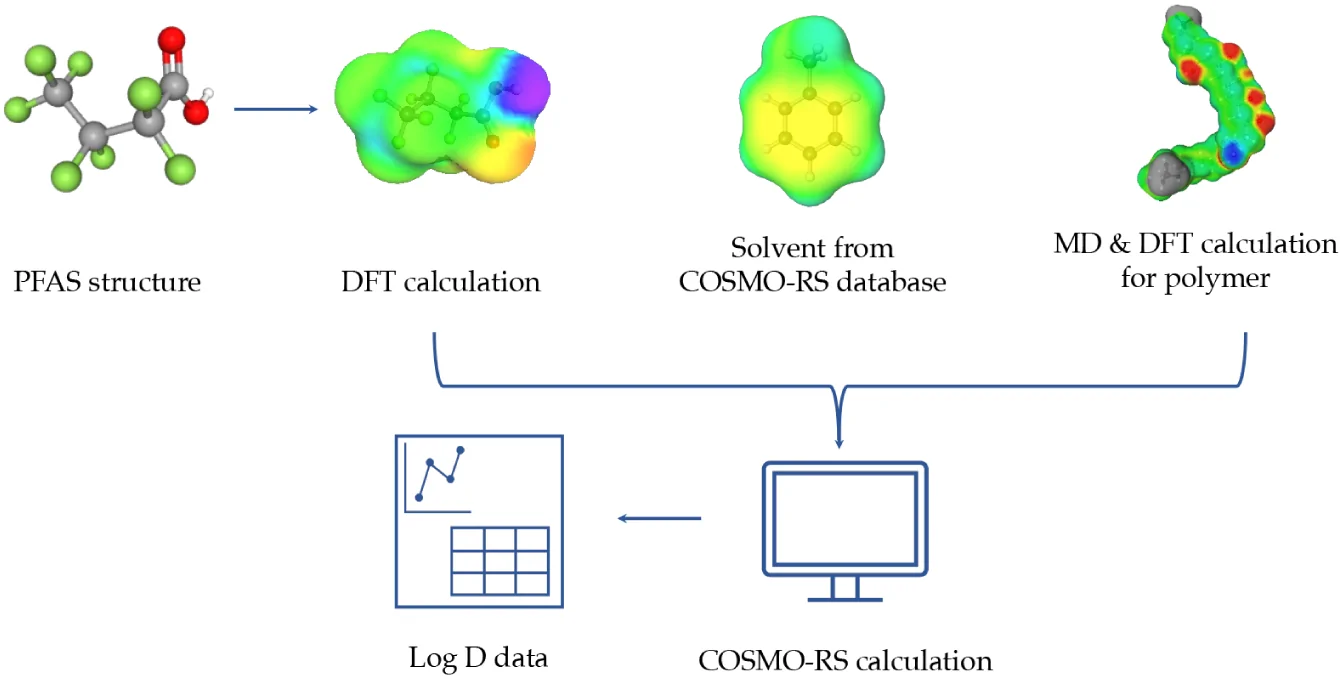
Summary of the computational workflow for predicting log *D* of PFAS contaminants across polymer–solvent systems. DFT: density functional theory, COSMO-RS: conductor-like screening model for real solvents, MD: molecular dynamics. Representative screening charge densities for three components (PFAS: perfluorobutanoic acid, solvent: toluene, and polymer: polyethylene (PE)) are shown and are used as inputs to COSMO-RS for all log *D* calculations. The PE polymer (represented by its oligomer) has its terminal groups deactivated to represent the corresponding polymer.

The screening charge densities (i.e., the COSMO files) for each component (PFAS, solvent, and polymer) serve as inputs into COSMO-RS for log *D* calculations at room temperature as per the work done by Zhou and coworkers.^11^ All COSMO-RS calculations were performed using the COSMOtherm19 software with the BP_TZVP_19 parametrization.^87^

### 3.2 Similarity Threshold Analysis

The graph representation of the PFAS space requires the specification of an appropriate molecular similarity threshold, ϵ, to define connectivity between nodes (i.e., PFAS structures). For this application, however, the threshold must be selected such that connected PFAS exhibit similar partitioning behavior. Accordingly, this section aims to determine an appropriate similarity threshold for the graph representation. Specifically, we seek to answer the following question: to what extent must two PFAS structures be structurally similar to exhibit analogous partitioning behavior?

To determine ϵ, a couple of chemically distinct solvents—dimethyl sulfoxide (DMSO) and toluene—and polymers—PE and EVOH—forming chemically unique polymer–solvent systems, were employed. Their screening charge densities and corresponding σ-profiles (Figure S10 in the SI) highlight their chemical differences, thereby providing a chemically diverse basis for evaluation. Due to the computational cost of performing these molecular calculations, a set of 50 PFAS structures was selected for this analysis. While a small subset of these compounds was chosen in part based on their documented detection in the environment and in food samples purchased from U.S. grocery stores,^92,93^ most of these PFAS were selected randomly to span different functional groups and ensure unbiased analyses. In particular, five PFAS were randomly selected from each of the following groups: sulfonic acids; cyclic/aromatic fluoroalkanes/fluoroalkenes; cyclic/aromatic ethers/carboxylic acids; aliphatic ethers/carboxylic acids; aliphatic amines/amides; and cyclic/aromatic amines/amides. Given their prevalence in the database, 15 additional PFAS were selected from the aliphatic fluoroalkane/fluoroalkene group, while the remaining five were selected from the FDA-identified PFAS list.^92,93^ These groups were chosen because they formed the broad structural groups of the entire PFAS space from the t-SNE analysis conducted in this study (*vide infra*). The complete list of the 50 PFAS is provided in the SI. The molecular calculations for the 50 PFAS required approximately 100 CPU-hours in total. The CPU-hours required for each PFAS were correlated with its chain length (i.e., electron count) in line with previously reported studies.^57,58^ Pairwise Tanimoto similarity using the MACCS keys molecular representation (i.e., eq 1, *s*(*u*,*v*)) was computed across all 50 PFAS, yielding 1,225 unique pairwise comparisons (i.e., excluding diagonal elements and redundant upper-triangular entries).

10 $$\left|\Delta \log D\right|=\max\left(0,\;\left|\log D_{u}-\log D_{v}\right|-1\right)+\lambda I\left(\log D_{u}\cdot \log D_{v}<-1\right)$$

The absolute log *D* difference in eq 28 between any PFAS pair explicitly captures the qualitative physicochemical distinction between partitioning behavior, as reflected by the sign of log *D*, thereby providing a more chemically meaningful measure of dissimilarity while accounting for molecular calculation uncertainties. Studies have investigated the use of COSMO-RS for predicting the physicochemical properties, including partition coefficient, of PFAS and fluorinated molecules and compared their predictions with experimentally derived data;^94,95^ these studies identified a ±1 log unit error associated with COSMO-RS-derived predictions. To account for this uncertainty, pairwise log *D* differences were corrected by subtracting 1 log unit from the absolute difference, flooring at zero to avoid negative values (see eq 28), where *u* and *v* are any PFAS pair. Also, computing the mere absolute log *D* difference treats changes of equal magnitude symmetrically, irrespective of transitions between partitioning behaviors. For example, a PFAS pair with log *D* values of −1 and 1 yields the same absolute difference (2) as a pair with values of 2 and 4. However, the former pair spans a change in sign indicating a shift in partitioning behavior, whereas the latter remains within the same physicochemical regime. Consequently, the former pair represents a more substantive chemical distinction than the latter, despite identical absolute differences. To account for such regime transitions, we incorporate a sign-change penalty into the absolute log *D* difference presented in eq 28; here, λ is a hyperparameter that penalizes PFAS pairs whose log *D* product is less than −1. In detail, λ penalizes PFAS pairs with significantly different partitioning behaviors, i.e., when the log *D* values are on opposing sides of the physicochemical threshold, defined here as one value below −1 and the other above +1. Lastly, I(·) is an indicator constraint that activates the penalizing term if the condition is satisfied. For instance, a PFAS pair with log *D* values of −0.9 and +0.9 should not be penalized based on sign change, as this PFAS pair in reality may have similar partitioning behavior due to molecular calculation error (±1 log unit).

Each point cloud in Figure 7 represents a unique PFAS pair, with the two panels reporting partitioning behavior between two different polymer–solvent systems. The Tanimoto structural similarity score is plotted along the *x*-axis, while the corresponding absolute log *D* difference, computed using eq 28, is plotted along the *y*-axis. In an ideal scenario, a strong monotonic inverse relationship would be observed, with data points aligning along a negatively sloped trend from the upper-left to the lower-right of the plots. Such a pattern would indicate a perfect correlation between structural similarity and physicochemical behavior, implying that structurally similar PFAS consistently exhibit comparable log *D* values and vice versa. However, this idealized behavior was not observed across the analyzed polymer–solvent systems; comparable log *D* values were primarily evident for only PFAS pairs with structural similarity scores of at least 70% (0.7), as reflected by the clustering of low |Δlog *D*| values in the lower-right region of the plots (see Figure 7 and Figure S10 of the SI). Structurally dissimilar PFAS pairs occasionally exhibited comparable log *D* values. For example, the red-highlighted point in Figure 7a corresponds to a PFAS pair (shown in Figure S11 of the SI) with a similarity of 9% and an absolute log *D* difference of 0.00 log units using eq 28. Conversely, moderately similar PFAS pairs may display substantial differences in partitioning behavior; the purple-highlighted point in Figure 7a represents a pair with 57% structural similarity but an absolute log *D* difference of 12.18 log units (see Figure S11 of the SI).

**Figure 7:**
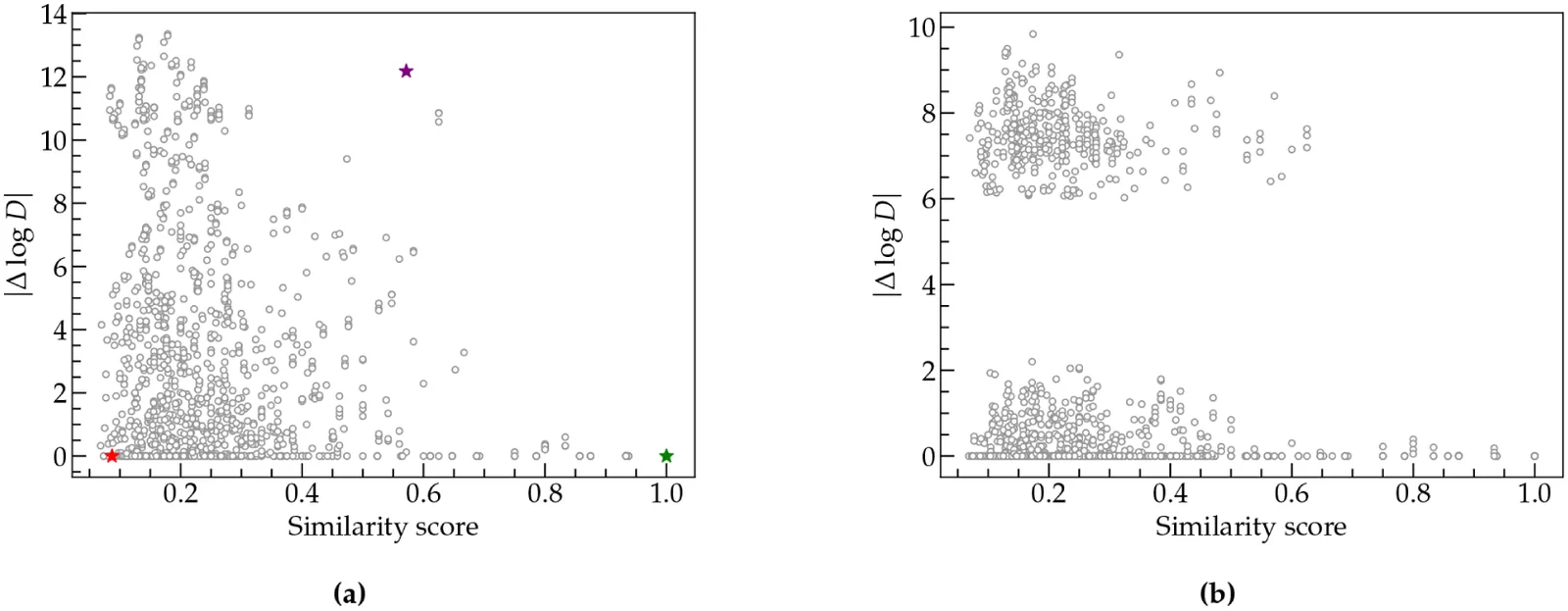
Similarity threshold analysis across polymer–solvent systems: (a) PE–DMSO system and (b) EVOH–toluene system. Three cloud points in the top-left plot are highlighted for further analysis. Results corresponding to the EVOH–DMSO and PE–toluene systems are presented in Figure S10 of the SI.

Overall, while low structural similarity does not preclude similar log *D* values, our analysis indicates that a structural similarity threshold of approximately 70% reliably ensures comparable log *D* values across the systems examined. An example of such a pair is highlighted in green in Figure 7a and Figure S11 of the SI. It is important to emphasize that this threshold is specific to the PFAS data set and the MACCS keys molecular representation employed in this study. For alternative data sets or fingerprinting methods, a similar analysis or domain-informed heuristics would be required to establish an appropriate similarity threshold.

## 4 Results and Discussion

### 4.1 Identifying Representative PFAS via T-SNE and K-Means Clustering

We first applied t-distributed stochastic neighbor embedding (t-SNE), a conventional dimensionality reduction technique particularly effective at revealing local cluster structures in high-dimensional data sets, followed by K-means clustering to identify structurally similar groups within the PFAS data set with the goal of identifying representative PFAS structures of the entire PFAS space with varying structural similarity thresholds.^96,97^ Subsequently, K-means and the elbow method were employed to clearly identify these clusters of PFAS, represented in the 2-D latent space.^98,99^ We note that the similarity threshold established in the preceding section was not applied in this section. Instead, K-means clustering and the elbow method were used solely to guide cluster formation, as these conventional data-driven approaches cannot enforce intracluster similarity thresholds and thus serve as a comparison against our alternative approach. The methodological implementation of t-SNE, K-means, and the elbow method is summarized in Text S7 of the SI. The results obtained from these analyses are presented in Figure 8.

**Figure 8:**
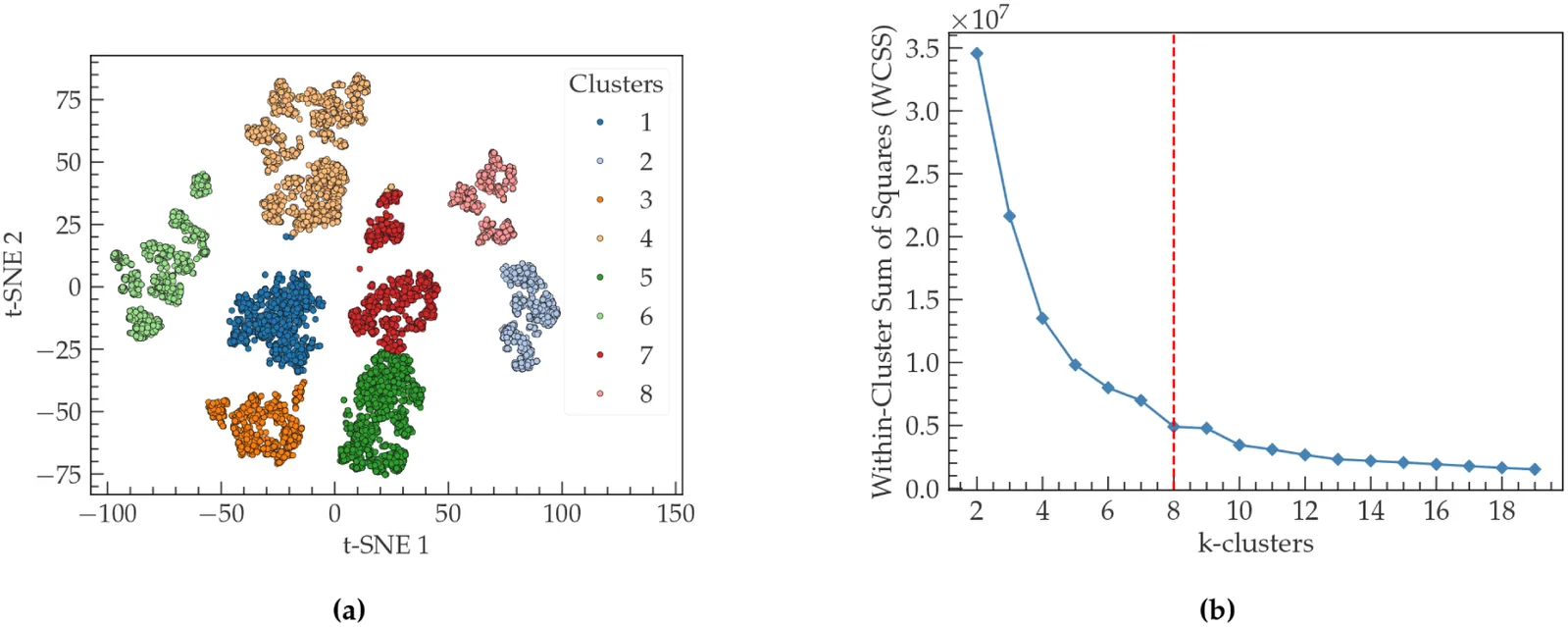
Clustering of the PFAS latent space. (a) 2-D t-SNE map showing eight identified PFAS families. (b) Elbow plot used to determine the optimal number of clusters (*k* = 8).

Eight distinct PFAS families were identified, depicted by the different color schemes presented in Figure 8a. The elbow plot (see Figure 8b) shows that beyond *k* = 8, the increment in the number of clusters only leads to a relatively marginal reduction in the Within Cluster Sum of Squares (WCSS) (see Text S7 of the SI); hence, *k* = 8 was determined as the optimal number of clusters. Text S8 and Figure S12 of the SI present detailed structural analyses of the eight PFAS families identified from the t-SNE analysis. A summary of the key functional groups defining each PFAS cluster, along with examples of PFAS structures from each cluster, is also provided in Table S1 of the SI. The results indicate that the combined application of t-SNE and K-means clustering effectively identifies PFAS families characterized by distinct functional groups and structural features. Each cluster can therefore be interpreted and described based on well-defined structural motifs with clear physicochemical features. However, the objective of this study extends beyond merely grouping PFAS into families with similar functional groups. Rather, our goal is to identify representative PFAS structures from highly similar PFAS groups that satisfy a predefined structural similarity threshold (e.g., ϵ = 0.7). To this end, we further evaluated each cluster to determine whether it is possible to identify such a representative PFAS structure within each cluster that meets a specified similarity criterion.

We first performed a pairwise similarity analysis within each cluster to characterize the distribution of structural similarity among the PFAS compounds. Specifically, the Tanimoto similarity was computed for all of the unique pairs of PFAS structures within each cluster. For example, in cluster 1, which contains 1,678 PFAS structures, the Tanimoto similarity was calculated for all unique structure pairs, excluding self-comparisons and duplicate pairs (i.e., recognizing that *s*(*u*,*v*) = *s*(*v*,*u*)). The resulting similarity values were then visualized as a distribution as presented in Figure 9 and Figure S13 of the SI. In an ideal scenario, one would expect PFAS structures within a given cluster to exhibit high structural similarity, resulting in a distribution strongly skewed toward higher similarity values (i.e., to the right); however, this behavior was not observed. Instead, the similarity across all clusters exhibited an approximately Gaussian distribution, indicating a substantial structural variability within each cluster. These results suggest that the clusters obtained via traditional data science approaches do not consist exclusively of highly similar structures, despite sharing common substructural features.

**Figure 9:**
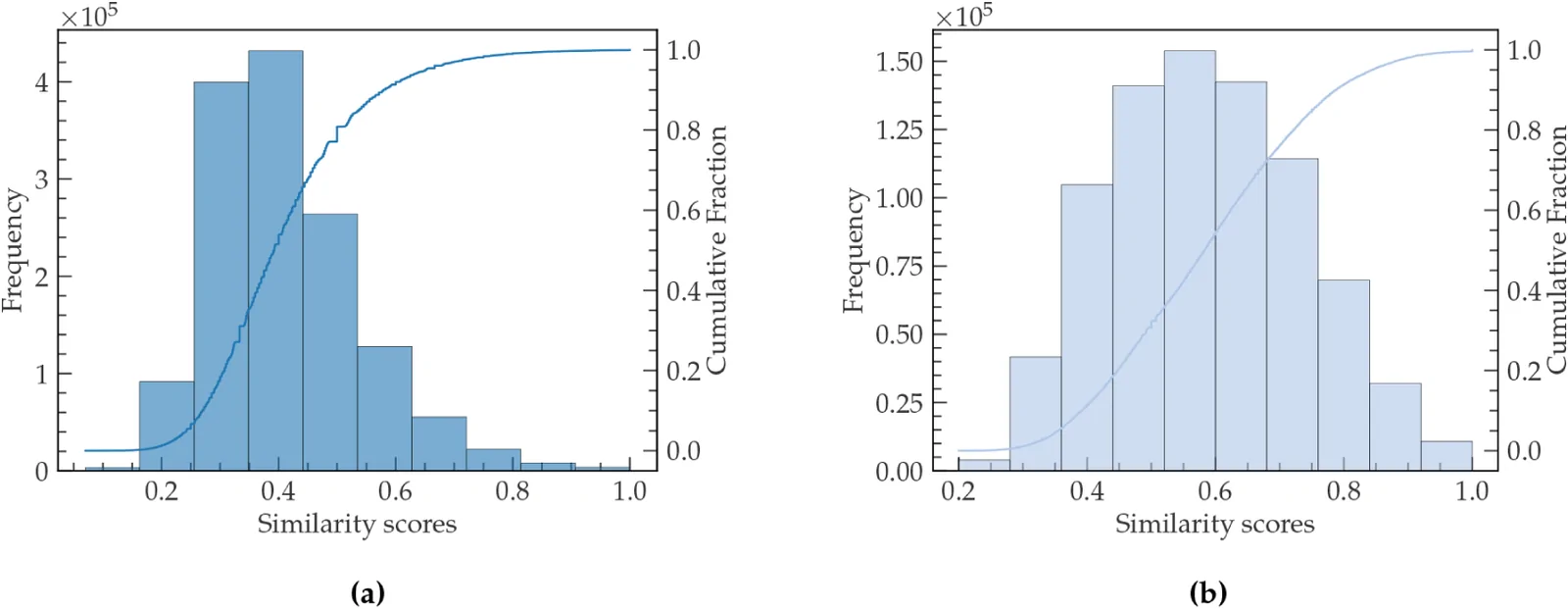
Distribution of pairwise PFAS structural similarity within each identified cluster: (a) Cluster 1 and (b) cluster 2. The complete distribution plots for all clusters are presented in Figure S13 of the SI. Colors in the distribution plots are consistent with the PFAS cluster color scheme used in Figure 8a.

The approximately Gaussian distribution of pairwise PFAS structural similarities observed within each cluster indicates that the traditional dimensionality reduction and clustering approaches captured localized regions of the PFAS chemical space; PFAS structures within a cluster share common structural motifs, as shown in Table S1 of the SI, while differing in smaller peripheral features. Note that the molecules listed in Table S1 of the SI are not representative molecules for the clusters identified using t-SNE and K-means clustering. Rather, they are illustrative examples of molecular structures within each cluster. Because the structural Tanimoto similarity is computed using a 167-bit fingerprint, the cumulative effect of these small structural variations tends to produce a symmetric distribution of pairwise similarities distributed approximately normally around a cluster-specific mean similarity. Additionally, K-means clustering minimizes WCSS, promoting compact groups of structurally related PFAS around a representative centroid.^98,100^ Since t-SNE preserves local neighborhood relationships during dimensionality reduction, these compact regions remain coherent in the embedded space via their probability functions (see Text S7 of the SI). Consequently, each cluster can be interpreted as a structural family centered around a representative PFAS with pairwise similarities distributed approximately normally around the cluster mean.

In summary, identifying representative PFAS structures for the entire PFAS chemical space that satisfy a predefined similarity criterion is challenging by using these methods. In particular, enforcing a specific similarity threshold, an essential requirement for the case study considered in this work, is not feasible when relying solely on traditional dimensionality-reduction and clustering algorithms. This limitation motivates the proposed graph-theoretic framework, which explicitly guarantees that every identified representative molecule satisfies the specified similarity threshold with all of the molecules it represents (i.e., its dominated or child molecules). This provides a rigorous and controllable criterion for representative selection that is not afforded by conventional clustering methods.

### 4.2 Identifying Representative PFAS via the Unweighted MDS Solution Approaches

We first represented the PFAS space as graphs using different similarity thresholds, ϵ, and subsequently applied the unweighted MDS solution approaches to the graphs; Table 1 shows the number of edges formed at different ϵ. Note that the number of nodes (*V*) is the total number of PFAS structures (14,722). Values of ϵ varying between 0.3 (30%) and 0.9 (90%) were adopted. Similar to Figure 2b, as ϵ increases (i.e., stricter thresholds), the number of formed edges reduces, leading to more isolated PFAS indicating distinct structural features.

**Table 1:** Summary of Results Obtained from Applying the Unweighted MDS Solution Approaches to Graphs Constructed at Varying Similarity Thresholds (ϵ), Evaluated under a 300 Second Time Limit^a^

Similarity threshold (ϵ)	No. of edges	|*S*_*H*_|	|*S*_*IP*_|	|*S*_*HIP*_|	MIP gap
0.3	29,966,836	17	23	17	inf
0.4	13,055,813	63	69	63	inf
0.5	5,906,127	183	111	111	0.24
0.6	2,427,930	566	359	359	0.05
0.7	992,494	1,436	1,103	1,103	0.00
0.8	372,039	2,824	2,555	2,555	0.00
0.9	119,533	5,216	5,082	5,082	0.00

a The total number of nodes is the total number ofPFAS structures (14,722). Here, $\left|S_{H}\right|$ , $\left|S_{IP}\right|$ , and $\left|S_{HIP}\right|$ denote the sizes of the minimum dominating sets (MDS) obtained using the heuristic (H), integer programming (IP), and hybrid heuristic–IP (HIP) approaches, respectively. The MIP gap represents the relative difference between the best known feasible solution and the optimal or best-bound solution. *inf* indicates an infinite MIP gap, i.e., while a feasible solution is found, its optimality is unknown within the set time limit.

The sizes of the MDS obtained from the different unweighted MDS formulations are also presented in Table 1 and visualized in Figure 10a. Gurobi MILP solver (version 12.0.1) was used to solve all the optimization problems presented in this work.^53^ First, as expected, the sizes of the MDS increase as stricter thresholds are implemented; this observation is logical as fewer edges (i.e., connections) are formed between PFAS structures, forming more isolated graph nodes (PFAS). Furthermore, it was observed that at ϵ ≥ 0.5, the sizes of the dominating sets obtained from the heuristic approach $\left(|S_{H}|\right)$ were consistently higher than those obtained from the IP approach $\left(|S_{IP}|\right)$ . This observation suggests that the IP approach outperforms the heuristic approach in less dense graphs (i.e., graphs with fewer edges). On the contrary, the heuristic approach proved to be more suitable for highly dense graphs, evident by the smaller-sized MDS solutions found at ϵ ≤ 0.4; the heuristic approach prioritizes *quickly* finding a feasible solution to the MDS problem.

**Figure 10:**
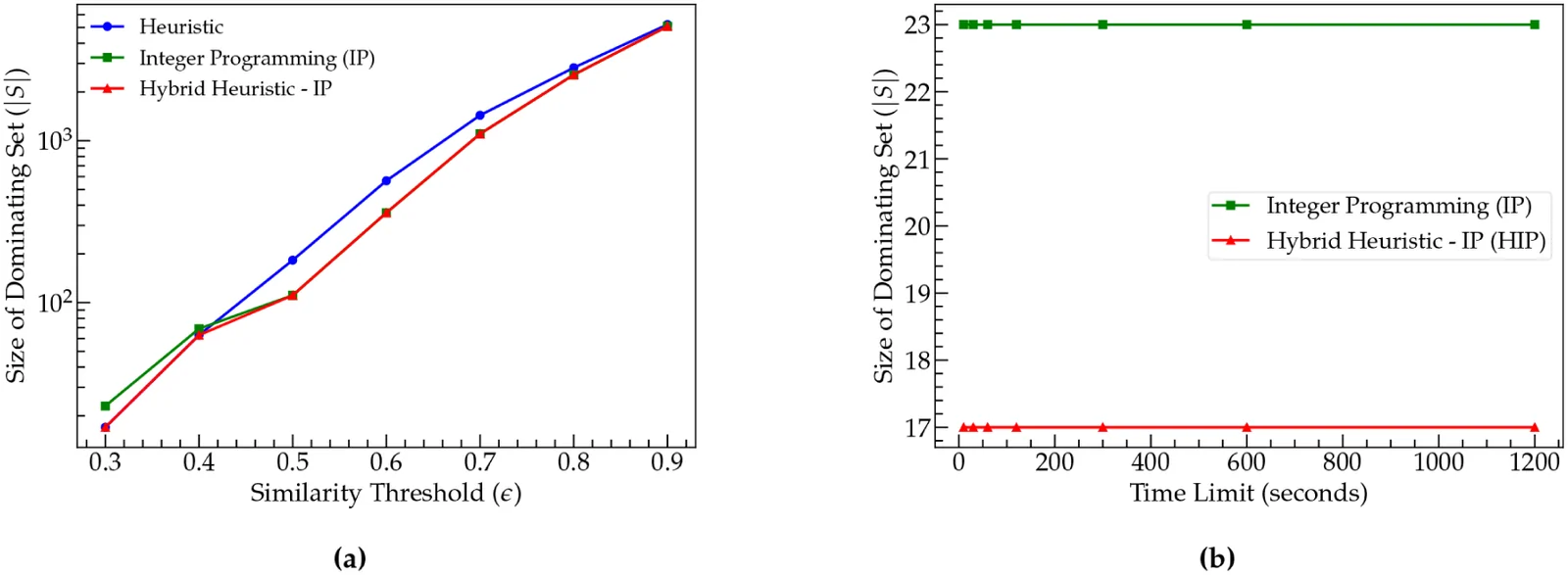
Solutions obtained from the different unweighted MDS formulations. (a) Comparisons between the heuristic, integer programming, and hybrid heuristic–IP approaches and (b) MDS solutions at different time limits for the graph corresponding to ϵ ≥ 0.3.

However, because an extra element in the MDS may correspond to hours of molecular simulations or expensive, laborious experiments, finding the optimal possible solution (i.e., the MDS) is highly beneficial. The novel hybrid heuristic–IP (HIP) approach presented in this work consistently outperformed or was as good as the MDS solutions obtained from the heuristic and IP approaches across all similarity thresholds (see Table 1 and Figure 10a). In the implementation of the hybrid heuristic–IP approach, the heuristic approach was used as a warm start, and the Gurobi solver tried to find better solutions. Although at least a feasible solution was found for all graph sizes presented in Table 1, in denser graphs (i.e., for ϵ = 0.3–0.5), large optimality gaps were observed within the 300 s time limit. For ϵ = 0.3 and ϵ = 0.4 within the 300 s time limit, an infinite optimality gap was observed, implying that because only one feasible solution was obtained, the optimality of the solution is unknown. To show the computational benefits of the hybrid heuristic–IP approach, we solved for the MDS of a dense graph with *s*(*v*,*u*) ≥ 0.3 using both the IP and the hybrid heuristic–IP approaches at different time limits. Figure 10b shows that even with an increased time limit of 1,200 s, the hybrid heuristic–IP approach consistently offered a better solution than the IP approach. Furthermore, as shown in Figure 10b, increasing the solver time limit from 10 to 1200 s did not improve the MDS solution, indicating that solution quality is largely insensitive to additional computation time for the graph sizes considered.

Based on the obtained MDS results presented in Table 1, adopting ϵ ≥ 0.7, we observe that the entire set of 14,722 PFAS structures can be represented by 1,103 PFAS structures. This implies that the number of molecular simulations required to screen solvents to target the entire PFAS data set is cut down by about 92%. The top five representative structures (dominating nodes) with the largest number of similar structures (dominated nodes) and their structures are presented in Table 2. Example solvents with positive distribution coefficients (log *D*) in PE and their values are also presented in Table 2; these solvents would be expected to extract the corresponding group of PFAS from PE. Based on these results, we observe that these five PFAS structures are able to represent 2,168 unique PFAS structures.

**Table 2:**
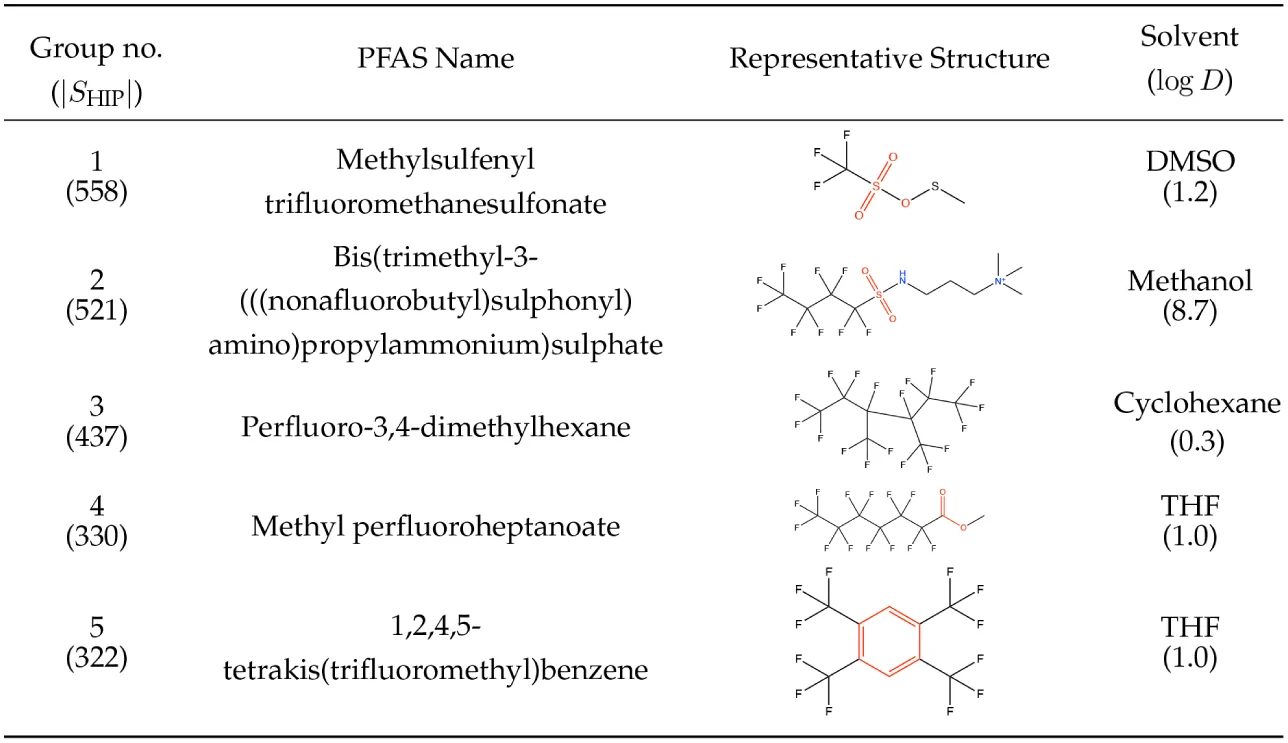
Top Five Representative Structures of the PFAS Chemical Space with the Most Uniquely Similar PFAS Structures^a^

a The number in brackets $\left(|S_{\mathrm{HIP}}|\right)$ is the number of unique PFAS structures that are at least 70% similar to the representative structure. The reported Log *D* values are the distribution coefficients of each PFAS between PE polymer and the corresponding solvent. The highlighted substructures (in red) show the common functional group across all PFAS structures within each group (see Figures S16 and S17 of the SI).

Figure S14 of the SI presents a detailed structural analysis of the five largest PFAS groups identified by using the proposed MDS-HIP framework (Table 2). Comparison of these results with the t-SNE/K-means clustering analysis (Figure S12 of the SI) reveals substantial agreement in the dominant structural motifs identified by both approaches. For example, group 1 identified by the MDS framework and cluster 8 obtained from the t-SNE approach are both characterized by sulfonic/sulfonate-containing PFAS. As shown in Figures S12 and S14, 100% of the molecules in these groups contain the sulfonyl motif. PFAS common to both groups include sodium 8-chloroperfluoro-1-octanesulfonate, perfluoro-2-butenesulfonic acid, and 5-(pentafluorosulfanyl)­perfluoropentyl sulfonic acid (see Figure S15a, S15b, and S15c of the Supporting Information).

Despite this overall agreement, the proposed graph-theoretic framework provides a finer level of structural discrimination by distinguishing molecules with subtle but chemically meaningful differences. For example, perfluoro-3-ethenyloxy-1-propanesulfonyl fluoride, perfluoro­(3-[1-(ethenyloxy)­propan-2-yl]­oxypropane-1-sulfonyl) fluoride, and 2,3,3,4,4-pentafluorothiolane 1,1-dioxide are assigned to cluster 8 by the t-SNE approach but are excluded from group 1 identified by the MDS framework. These structures are shown in Figure S15d, S15e, and S15f of the Supporting Information. Although these PFASs share the same sulfonyl functional group as the group 1 representative molecules, they also contain additional structural motifs, such as alkene double bonds, ether, and cyclic substructures, that reduce their overall structural similarity below the prescribed threshold (e.g., 70%). Consequently, the graph-theoretic framework assigns them to different PFAS groups. This distinction highlights a key advantage of the proposed framework over that of conventional clustering methods. Rather than grouping molecules based primarily on global embedding proximity, the MDS framework guarantees that every PFAS assigned to a representative molecule satisfies the prescribed minimum structural similarity threshold, thereby ensuring a representative group that dominates all molecules in the chemical space.

Furthermore, for illustration purposes, Figures S16 and S17 of the Supporting Information present some example structures found in groups 1 and 4 of Table 2. In particular, groups 1 and 4 show PFAS families with only sulfonate and carboxylate substructures, respectively. Our proposed large chemical-space navigation framework makes it possible to identify such representative compounds of the entire space with varying predefined similarity thresholds. For instance, if a much higher predefined ϵ = 0.9 is used, the number of required molecular simulations will still be reduced by 65%, implying that the PFAS database used in this study contains highly similar PFAS structures. These observations imply that the PFAS class of chemicals can be targeted by extensively studying a reduced number of structures that are representative of the entire space.

It is important to note that although the proposed chemical space navigation framework is agnostic to the choice of molecular representation, the resulting MDS solution may vary with the representation used. This is because different molecular representations capture structural features at different levels of detail, resulting in different pairwise similarity values and consequently graph structures. For example, MACCS keys assign a Tanimoto similarity of 1.0 to perfluorobutanedioic acid and perfluoropentanedioic acid, despite their different carbon-chain lengths (Figure S18 of the Supporting Information). In contrast, Morgan fingerprints distinguish these molecules and, therefore, produce a different graph structure and MDS solution. More generally, Morgan fingerprints provide greater structural discrimination and typically yield lower similarity values than those of MACCS keys. To demonstrate the applicability of the framework to alternative molecular representations, we also obtained an MDS solution using Morgan fingerprints, as described in Text S9 of the Supporting Information. For the remainder of the paper, all analyses were performed using MACCS keys unless stated otherwise. This representation was used because the study by Zhou and co-workers^11^ reported that small-molecule PFAS can be grouped according to their primary functional groups (e.g., carboxylic and sulfonic acids), with a given solvent capable of removing PFAS within a functional group class regardless of carbon-chain length. MACCS keys provide a computationally efficient representation that captures these key functional group features and were therefore considered appropriate for demonstrating the proposed framework in this case study.

### 4.3 Identifying Representative PFAS via the Weighted MDS Solution Approaches

We also sought to identify the MDS of the PFAS space by using a weighted solution approach. Contrary to the unweighted MDS solution approaches, each structure (i.e., node) in the graph is not considered equal; each node is assigned a weight vector **W**_*v*_ with *k* components. Here, we build a graph and assign its *number of electrons* (*N*_e_) to each structure within the graph. In this case, we adopt the *number of electrons* as an attribute of each PFAS structure because this property significantly impacts computational cost during molecular simulations.^57,58^ The number of electrons for each structure is computed as *N*_e_ = sum­(*Z*_i_) – *q*, where *Z*_i_ is the atomic number of each element in the structure and *q* is the sum of the formal charges on the molecule. Since an explicit bound on *N*_e_ for the PFAS structures contained in the MDS is not known for this application, we apply both the weighted heuristic (Algorithm 3) and the weighted hybrid heuristic–IP (eq 21) approaches with *unknown feature bounds* to compute the MDS. Therefore, the objective is not only to identify an MDS but also to minimize the total number of electrons associated with selected PFAS structures. The resulting solutions to the weighted MDS problem, evaluated across different graph sizes (i.e., varying similarity thresholds), are presented in Figure 11 and Table S4 of the Supporting Information.

**Figure 11:**
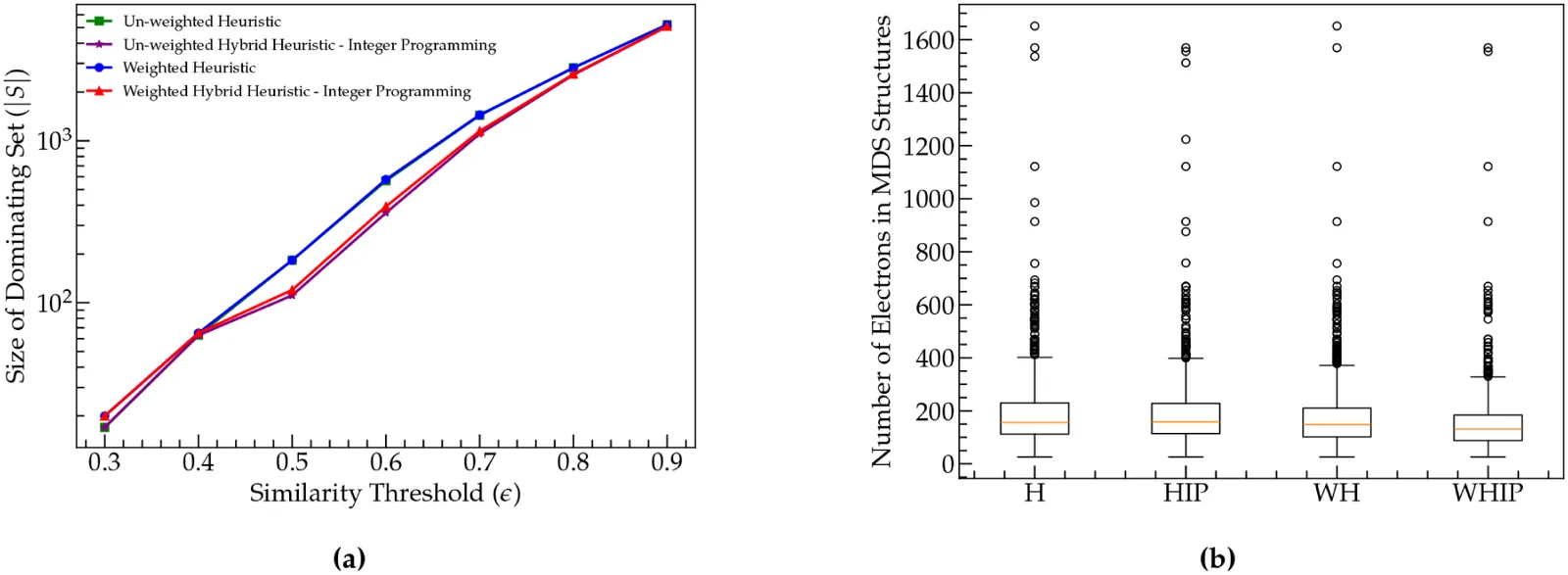
Solutions to the weighted MDS problem with unknown bounds. (a) Comparison between unweighted and weighted MDS formulations. (b) Box plot showing the distribution of electron counts for PFAS structures included in the MDS, obtained using the unweighted heuristic-based (H), unweighted heuristic-based integer programming (HIP), weighted heuristic-based (WH), and weighted heuristic-based integer programming (WHIP) methods. The interquartile ranges for H, HIP, WH, and WHIP are 112.0–230.0, 114.0–228.0, 102.0–210.0, and 88.0–184.0, respectively, while the corresponding average electron counts are 186.8, 118.6, 172.9, and 154.4.

Consistent with the observations from the unweighted MDS approach, the weighted hybrid heuristic–IP approach consistently provides solutions that are either better or comparable with those obtained using the weighted heuristic approach. Furthermore, when comparing the solutions from the unweighted and weighted hybrid heuristic–IP approaches (see Figure 11a), the unweighted approach generally yields smaller MDS sizes, due to the additional constraint placed on the molecular property *k* (i.e., electron count) of molecules in the weighted MDS approach. More importantly, given that our objective is to simultaneously minimize the MDS size while prioritizing structures with lower electron counts, we analyzed the distribution of the number of electrons for structures included in the MDS solutions obtained from the unweighted heuristic (H), unweighted hybrid heuristic–IP (HIP), weighted heuristic (WH), and weighted hybrid heuristic–IP (WHIP) approaches. These results are summarized using a box plot in Figure 11b. Overall, the MDS obtained via the WHIP approach contains structures with the lowest electron counts, as evidenced by the smallest average number of electrons (154.4) and the narrowest interquartile range (88.0–184.0), representing the middle 50% of the data. Additionally, the maximum number of electrons within the upper whisker is the lowest for the WHIP method (328.0).

The box plot in Figure 11b reveals the presence of outliers corresponding to structures with significantly higher electron counts. For instance, the structure with the largest number of electrons in the MDS obtained via the WHIP approach has 1,570 electrons. This structure corresponds to an isolated node (i.e., not similar to any other structure) in the graph at ϵ = 0.7, indicating a highly unique structural profile (see Figure S19 of the Supporting Information). Its inclusion in the MDS is therefore necessary, as all nodes in the graph must be dominated by at least one selected structure (see eq 21). Furthermore, this observation indicates that incorporating *hard constraint(s)* presented in Algorithm 2 and eq 5, where selected structures are restricted to those with electron counts below a specified threshold (e.g., *θ* = 328.0), may result in solutions where some structures are not dominated. In practice, this arises when isolated nodes such as the aforementioned PFAS structure may not be dominated by any structure within the MDS and are themselves excluded by the imposed hard constraint(s).

### 4.4 Identifying the MIDS of the PFAS Space

We also solved the weighted minimum independent dominating set (WMIDS) problem of the PFAS space to identify a set of independent (i.e., structurally nonadjacent and thus unique) representative PFAS that both cover the entire chemical space and minimize the total electron count. First, we construct a graph, assigning to each node (PFAS) its corresponding number of electrons (*N*_*e*_). We then apply the hybrid heuristic–IP approach presented in eq 8, with the objective function reformulated as ∑_*i∈V*_ *w*_*v*,*k*_ · *x*_*i*_ (i.e., for weighted graphs) to simultaneously minimize the total electron count of the selected PFAS structures. The resulting WMIDS solutions across different graph sizes (i.e., varying similarity thresholds) are reported in Table 3 and Figure S20 of the SI. For comparison, the MDS solutions obtained via all approaches presented in this work are also included in Table 3.

**Table 3:** Summary of the MDS and MIDS Results Obtained Using Different Approaches: Unweighted Integer Programming (IP), Weighted Heuristic (WH), Weighted Hybrid Heuristic–IP (WHIP), and Weighted Minimum Independent Dominating Set (WMIDS)^a^

	Size of dominating set				No. of edges in dominating set			
Similarity threshold (ϵ)	$\left|S_{\mathrm{IP}}\right|$	$\left|S_{\mathrm{WH}}\right|$	$\left|S_{\mathrm{WHIP}}\right|$	$\left|S_{\mathrm{WMIDS}}\right|$	IP	WH	WHIP	WMIDS
0.3	23	20	20	20	51	0	0	0
0.4	69	65	65	65	150	0	0	0
0.5	111	183	120	183	101	0	122	0
0.6	359	577	393	577	218	0	296	0
0.7	1103	1443	1148	1205	333	0	489	0
0.8	2555	2820	2579	2648	293	0	453	0
0.9	5082	5207	5085	5137	156	0	192	0

a The WMIDS extends the weighted hybrid heuristic–IP approach by incorporating an additional constraint that enforces independence among the selected nodes Here, $\left|S_{\mathrm{IP}}\right|$ , $\left|S_{\mathrm{WH}}\right|$ , $\left|S_{\mathrm{WHIP}}\right|$ , and $\left|S_{\mathrm{WMIDS}}\right|$ denote the sizes of the dominating sets obtained from each approach. The “No. of edges in dominating set” indicates the number of edges in the induced subgraph of selected nodes, where a value of zero corresponds to an independent dominating set and any nonzero value indicates a connected dominating set .

To assess whether the resulting dominating sets are independent, we examined the induced subgraphs formed by the selected nodes and recorded the number of edges among them; a value of zero indicates an independent dominating set, whereas any nonzero value indicates a connected dominating set. As shown in Table 3, the solutions obtained via the weighted heuristic (WH) and weighted minimum independent dominating set (WMIDS) formulations consistently yielded independent dominating sets across all graph sizes, as evidenced by the absence of edges among selected nodes. As observed in Algorithm 3, the weighted heuristic (WH) approach inherently enforces independence, since the selection of any node results in the removal of both the node and its neighbors from the graph. In addition, the WMIDS solution not only enforces independence but also minimizes the size of the dominating set, as demonstrated in Table 3 (i.e., comparing $\left|S_{\mathrm{WH}}\right|$ with $\left|S_{\mathrm{WMIDS}}\right|$ ). Conversely, while the MDS solutions obtained via the integer programming $\left(|S_{\mathrm{IP}}|\right)$ and weighted hybrid heuristic–IP $\left(|S_{\mathrm{WHIP}}|\right)$ approaches often produce smaller dominating sets, they do not consistently guarantee independence.

Finally, as illustrated in Figure S20b of the Supporting Information, the box plot showing the electron count distributions indicates that although the WHIP method yields solutions with the lowest overall electron counts, the WMIDS formulation provides comparable performance and outperforms both the IP and WH methods while maintaining independence with the DS. In summary, the *modified* weighted hybrid heuristic–IP approach (WHIP, eq 8) effectively solves the *minimum independent dominating set* problem, producing solutions with competitive set sizes while prioritizing structures with low electron counts and satisfying all model constraints. Moreover, these IP approaches offer the flexibility to incorporate additional logical constraints that are tailored to specific application requirements.

## 5 Conclusions and Future Work

The identification of representative molecules from large chemical spaces remains a critical challenge across applications in chemical and data sciences. Traditional dimensionality reduction and clustering techniques exhibit notable limitations in addressing this problem, particularly when the objective is to identify representative sets with predefined similarity constraints. In this work, we develop a framework that integrates graph theory and integer programming to systematically identify such subsets, referred to as the *minimum dominating set* (MDS). By leveraging cheminformatics, large chemical spaces are modeled as graphs, where nodes correspond to molecules and edges encode pairwise structural similarity. Within this framework, the MDS corresponds to the smallest subset of molecules that collectively “dominate” the graph, i.e., each molecule in the chemical space is either included in the subset or is sufficiently similar to at least one selected representative. To address different modeling requirements, we present several MDS solution approaches across three broad categories: *unweighted graphs*, *weighted graphs*, and *minimum independent dominating set* (MIDS) for both unweighted (MIDS) and weighted (WMIDS) graphs. Within these categories, we develop heuristic, integer programming, and hybrid approaches, enabling the efficient computation of high-quality or optimal solutions to the MDS problem across a range of graph sizes.

We applied our framework to the PFAS chemical space, comprising over 14,722 structures, with the objective of identifying representative PFAS molecules that can be used to efficiently screen and select solvents capable of targeting the broader chemical space. By integrating cheminformatics descriptors with molecular simulations, we established a correlation between structural similarity and key molecular properties, specifically the distribution coefficient (log *D*). Notably, the application of this framework enabled a reduction of 65–92% in the number of molecular simulations that would be required to compute (log *D*) values while maintaining representative coverage of the PFAS space. Also, the complete workflow, from generating molecular fingerprints, computing the pairwise Tanimoto similarity, and constructing the graph to identifying the MDS, requires less than 500 s depending on the graph size, making the proposed framework computationally efficient and readily scalable to large chemical data sets. Compared to t-SNE and K-means clustering methods, the proposed framework more effectively identifies representative molecules across varying graphs with different similarity thresholds and is readily extendable to other large chemical spaces and data sets. Furthermore, our framework, particularly the *weighted MDS* and *weighted MIDS* approaches, demonstrates a strong potential for integration into machine learning workflows in molecular science. These formulations enable the simultaneous identification of structurally diverse representative molecules while prioritizing selections based on relevant physicochemical properties such as electron count. Such an approach can be adapted for other molecular properties, such as cost, solubility, viscosity, etc.

Beyond its comparison with t-SNE followed by K-means, the proposed framework provides several advantages over conventional clustering approaches for representative selection. Unlike methods that primarily partition the chemical space into clusters such as single-linkage hierarchical clustering, the proposed MDS formulation directly identifies a minimum set of representative molecules that provide complete coverage of the chemical space subject to a predefined similarity threshold. Furthermore, the optimization formulation allows application-specific molecular properties and selection constraints to be incorporated directly into the representative-selection problem, providing greater control over which molecules are selected for simulations or experiments. A further advantage is that the similarity threshold is explicitly encoded in the graph construction, providing a direct and interpretable relationship between the desired level of molecular similarity and the resulting representative sets. This distinguishes the proposed framework from approaches that are susceptible to the chaining effect or those requiring a separate procedure to select representatives after clustering.

We note that the current choice of molecular representation and similarity metric may introduce important limitations for other chemical space applications, other than PFAS. Specifically, the use of MACCS keys in conjunction with the Tanimoto similarity coefficient does not adequately capture differences in the molecular chain length among structurally related compounds. For example, the Tanimoto similarity between perfluorobutanoic acid *C*_4_ *HF*_7_ *O*_2_ and perfluorooctanoic acid *C*_8_ *HF*_15_ *O*_2_ is computed as 1.00 using MACCS keys despite the clear difference in chain length. This arises because both molecules share identical structural motifs (i.e., similar atom types and bonding patterns), leading to indistinguishable fingerprint representations in this scheme. The Morgan fingerprint offers a different level of detail in differentiating between molecular structures as illustrated in this study. Also, depending on the specific application, such structural nuances can be more effectively captured by using advanced molecular representations, particularly graph-based approaches that explicitly encode connectivity and substructural variations. Future work will therefore focus on integrating more expressive and generalizable molecular representations into the proposed framework for broader application.

Furthermore, instead of preselecting a similarity threshold, future work could extend the proposed framework to a multiobjective MDS formulation that simultaneously minimizes the MDS size and maximizes the similarity threshold within each molecular family. In this formulation, ϵ would be incorporated as edge weights representing pairwise molecular similarity. In addition, we envision extending this framework within an active-learning framework applied to larger and more diverse chemical data sets. In this setting, the framework can provide a well-representative subset of molecules for initial model training, after which active learning strategies can iteratively prioritize molecules with targeted physicochemical properties. This integration has the potential to significantly enhance efficiency and decision-making in cheminformatics and molecular science applications.

## Supplementary Material



## Data Availability

All data, including the list of the 50 PFAS used in the similarity threshold analysis, and code necessary to replicate the analyses done in this work are publicly available at GitHub via https://github.com/Ikegwuugo/Research_DS.

## Notes

Additional methodological details, approaches, data preparation and classification procedures, and analyses supporting the main text; Texts S1 and S2 describing additional approaches for solving weighted MDS problems; Text S3 detailing the formulation and solution approach for the MIDS problem; Text S4 describing PFAS data curation and cleaning; Text S5 detailing the classification of the PFAS database; Text S6 presenting the polymer modeling methodology used in this study; Text S7 providing the theoretical basis of the t-SNE and K-means clustering algorithms; Text S8 presenting a structural analysis of the PFAS clusters identified from the t-SNE analysis; Text S9 presenting MDS results obtained by applying the unweighted MDS formulation to the PFAS data set using Morgan fingerprint molecular representations; and four tables (Tables S1–S4) and 20 figures (Figures S1–S20) supporting the analyses presented in the main text and Supporting Information (PDF)
