# Supplementary material for: Navigating Large Chemical Spaces Using Graph Theory and Integer Programming
Source: J Chem Inf Model. 2026 Sep 7;66(18):11862–81. doi: 10.1021/acs.jcim.6c01810 (PMC13625972; doi:10.1021/acs.jcim.6c01810)
Supplement: Supplementary file 1 [file ci6c01810_si_001.pdf]

Supporting Information for Navigating Large Chemical Spaces using Graph Theory and Integer Programming

Ugochukwu M. Ikegwu[a],

Reid C. Van Lehn[a,b*], and Victor M. Zavala[a*]

[a]Department of Chemical and Biological Engineering

University of Wisconsin-Madison, Madison, Wisconsin, 53706, USA.

[b]Department of Chemistry

University of Wisconsin-Madison, Madison, Wisconsin, 53706, USA.

*Corresponding Authors: victor.zavala@wisc.edu and vanlehn@wisc.edu.

Text S1: Pre-selection approach

In this approach, the IP is reformulated as presented in eq S1. Herein, $\mathcal{V}_\theta$ is the set of nodes (i.e., molecules) that satisfy the logical constraint on the molecular property k, and is defined as $\mathcal{V}_\theta = \{i \in V \mid w_{i,k} \leq \theta \text{ or } w_{i,k} \geq \theta\}$, where all variables retain its meaning as described in the main text. For instance, using Figure 4 of the main text, where $w_{i,k} = w_{i,1}$ and $\theta = 350\text{g/mol}$, therefore, $\mathcal{V}_\theta = \{i \in V \mid w_{i,1} \leq 350\text{g/mol}\} = \{v_2, v_3, v_6\}$. Here, the domination constraint (i.e., the first constraint) can be expanded as eq S2.

$$\begin{aligned} \min \quad & \sum_{i \in \mathcal{V}_\theta} x_i \\ \text{s.t.} \quad & x_j + \sum_{i \in \mathcal{N}_G(j) \bigcap \mathcal{V}_\theta} x_i \geq 1, \quad \forall j \in \mathcal{V}_\theta, \\ & \sum_{i \in V} x_i \leq |\mathcal{S}_{\text{WH}}|, \\ & x_i \in \{0,1\}, \quad \forall i \in \mathcal{V}_\theta. \end{aligned} \tag{S1}$$

Observe that the domination constraint in eq S1 only considers nodes from the pre-selected set. For example, looking at the expanded form in eq S2, only constraints placed on nodes v_2, v_3, and v_6 are included. Consequently, from eq S2, we observe that setting $x_3 = x_6 = 1$ satisfies all constraints while minimizing the objective function in eq S1. The resulting MDS is $\underline{\mathcal{S}}_{\text{WHIP}} = \{v_3, v_6\}$. Additionally, in the case where the upper and lower bounds on the molecular feature(s) are known, the pre-selected feasible nodes $\mathcal{V}_\theta$ is modified to $\mathcal{V}_\theta = \{i \in V \mid \underline{\theta} \leq w_{i,k} \leq \overline{\theta}\}$, where $\underline{\theta}$ and $\overline{\theta}$ are the lower and upper bounds, respectively.

$$v_2: \quad x_2 + x_3 \geq 1 \tag{S2a}$$

$$v_3: \quad x_3 + x_2 \geq 1 \tag{S2b}$$

$$v_6: \quad x_6 \geq 1 \tag{S2c}$$

$$\sum_{i \in V} x_i \leq |\mathcal{S}_{\mathrm{WH}}|; \quad |\mathcal{S}_{\mathrm{WH}}| = 2 \tag{S2d}$$

Text S2: Limitations of hard-constrained approach on weighted graphs

A limitation of using this hard-constrained approach is that while an MDS of molecules that satisfy the imposed feature constraints is obtained, not all nodes (molecules) may be dominated (represented). This particularly comes up when a molecule whose molecular feature does not satisfy the logical constraint and is isolated, i.e., is not dominated by a node that satisfies the logical constraint. For example, suppose the aim is to find the MDS of a chemical space presented in Figure S1 adopted from Figure 2b of the main text with real molecules, such that all selected molecules should have molecular weight not more than $250\mathrm{g/mol}$ using the pre-selection approach (i.e., $\mathcal{V}_\theta = \{i \in V \mid w_{i,1} \leq 250\mathrm{g/mol}\}$). This implies that $\mathcal{V}_\theta = \{v_1, v_2, v_7\}$. Therefore, applying the IP presented in eq S1 without including the bounding constraint will seek to minimize the objective function $(x_1 + x_2 + x_7)$, $x_i \in \{0, 1\}$, and lead to the expanded domination constraint outlined in eq S3.

$$v_1: \quad x_1 \geq 1 \tag{S3a}$$

$$v_2: \quad x_2 \geq 1 \tag{S3b}$$

$$v_7: \quad x_7 \geq 1 \tag{S3c}$$

From Figure S1, we can observe that molecules v_1, v_2, v_3, and v_7 ideally form an MDS for the chemical space without enforcing the logical constraint. However, only v_1, v_2, and v_7 satisfy the logical constraint on the molecular weight (i.e., $w_{i,1} \leq 250\mathrm{g/mol}$). Therefore the solution to the MDS problem becomes $\underline{\mathcal{S}}_{\text{WIP}} = \{v_1, v_2, v_7\}$. This observation shows that the MDS solution obtained via this *hard constraint* approach may not fully cover the entire chemical space. This solution is, however, still useful in situations where experiments and molecular simulations are tightly constrained.

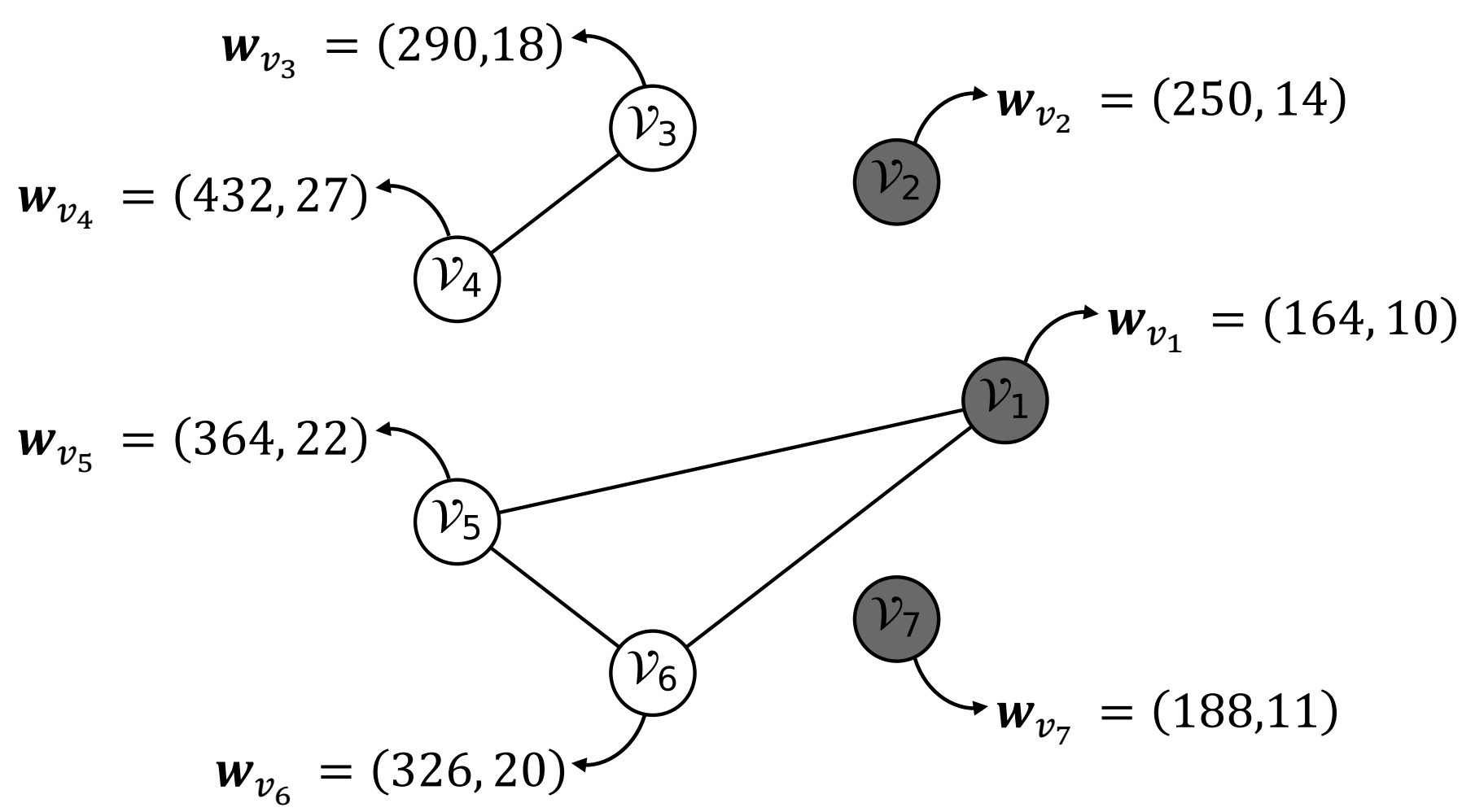

Figure S1: Weighted graph-theoretic representation of a chemical space comprising seven molecules: perfluoropropanoic acid (v_1), perfluoropropanesulfonic acid (v_2), perfluorobutylpentane (v_3), perfluorohexyloctane (v_4), perfluoroheptanoic acid (v_5), perfluorocyclohexanecarboxylic acid (v_6), and perfluoropropane (v_7). Each weight vector $\mathbf{w}_v$ encodes molecule-specific properties (i.e., molecular weight in $\mathrm{g/mol}$ and heavy atom count, respectively). Gray circles indicate nodes included in the MDS satisfying the logical constraint $w_{i,1} \leq 250\mathrm{g/mol}$ using the pre-selection approach. However, molecule v_3 is included in the MDS to satisfy all constraints in the IP formulation presented in eq S1.

Text S3: Computing the minimum independent dominating set (MDS)

Applying the reformulated IP in eq 8 of the main text to the graph presented in Figure 5 of the main text will yield the constraints presented in eq S4 where eq S4a – eq S4e represent the *domination constraints*, eq S4f – eq S4k represent the *independent domination constraints*, and eq S4l represents the *bounding constraint* obtained from the heuristic approach. Therefore applying the reformulated IP presented in eq S4, while satisfying all the constraints will yield several solutions including $x_1 = x_3 = 1$, $x_1 = x_4 = 1$, $x_2 = x_4 = 1$, $x_2 = x_5 = 1$, corresponding to MIDS solutions of the form $\underline{S}_{\text{MIDS}} = \{v_1, v_3\}$ or $\{v_1, v_4\}$ or $\{v_2, v_4\}$ or $\{v_2, v_5\}$, respectively.

$$v_1: \quad x_1 + x_2 + x_5 \geq 1 \tag{S4a}$$
$$v_2: \quad x_2 + x_1 + x_3 \geq 1 \tag{S4b}$$
$$v_3: \quad x_3 + x_2 + x_4 + x_5 \geq 1 \tag{S4c}$$
$$v_4: \quad x_4 + x_3 + x_5 \geq 1 \tag{S4d}$$
$$v_5: \quad x_5 + x_1 + x_3 + x_4 \geq 1 \tag{S4e}$$
$$e_{1,2}: \quad x_1 + x_2 \leq 1 \tag{S4f}$$
$$e_{1,5}: \quad x_1 + x_5 \leq 1 \tag{S4g}$$
$$e_{2,3}: \quad x_2 + x_3 \leq 1 \tag{S4h}$$
$$e_{3,5}: \quad x_3 + x_5 \leq 1 \tag{S4i}$$
$$e_{3,4}: \quad x_3 + x_4 \leq 1 \tag{S4j}$$
$$e_{4,5}: \quad x_4 + x_5 \leq 1 \tag{S4k}$$
$$\sum_{i \in V} x_i \leq |\mathcal{S}_{\text{H}}|; \quad |\mathcal{S}_{\text{H}}| = 2 \tag{S4l}$$

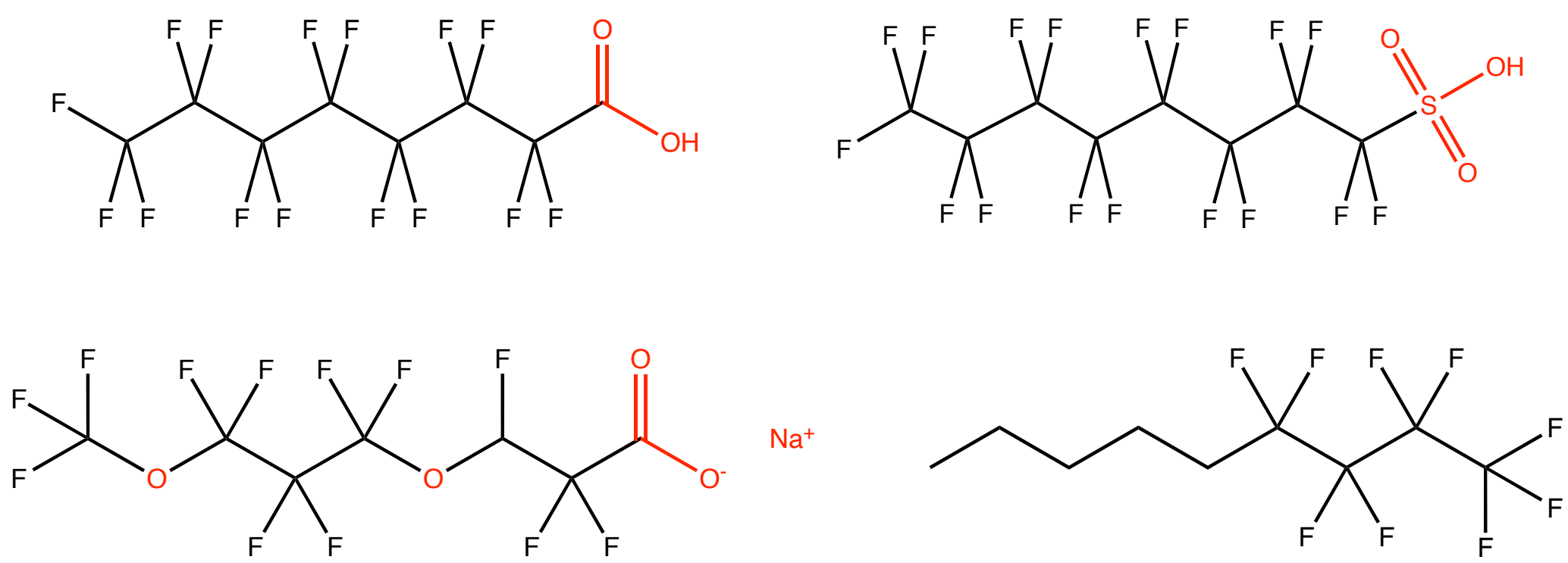

Figure S2: Examples of small-molecule PFAS with different functional groups. The highlighted (in red) substructures show the main functional groups of some common small-molecule PFAS, namely: carboxylic PFAS (top-left), sulfonic PFAS (top-right), ionized sulfonic PFAS (bottom-left), and alkane PFAS (bottom-right). Shown are perfluorooctanoic acid (PFOA, top-left), perfluorooctanesulfonic acid (PFOS, top-right), Sodium dodecafluoro-3H-4, 8-dioxanonanoate (bottom-left), and perfluorobutylpentane (bottom-right).

Text S4: PFAS data curation, summary, and pre-processing

Several researchers and regulatory agencies have attempted to establish a clear definition of PFAS. Although these varying definitions have complicated global identification and regulation, they are uniformly grounded in structural criteria.[1] For example, Buck and co-workers[2] defined PFAS as aliphatic substances containing at least one fully fluorinated carbon atom, specifically incorporating a perfluoroalkyl moiety ($-C_nF_{2n+1}-$). The state of Maine adopted a broader definition by describing PFAS as any fluorinated organic chemical containing at least one fully fluorinated carbon atom (i.e., beyond aliphatic substances).[3] This definition is largely consistent with that proposed by the Organization for Economic Co-operation and Development (OECD), which characterizes PFAS as chemicals containing at least one per-fluorinated methyl ($-CF_3$) or per-fluorinated methylene ($-CF_2-$) group.[4] However, subtle distinctions exist among these definitions. For instance, compounds containing the moiety R_3C-F, in which the only hydrogen substituent on the carbon is replaced by fluorine, would fall within the definitions of Buck et al. and the Maine legislation but would not meet the OECD criteria.

Given the existence of highly fluorinated compounds with diverse and complex structures, and the challenges associated with purely structural definitions, Gaines et al.[1] of the U.S. Environmental Protection Agency (EPA) proposed a more concise and operational definition of PFAS. Their objective was to establish a pragmatic reference framework to better focus regulatory and scientific efforts on substances of greatest environmental concern. The aforementioned study proposed a revised PFAS definition based on four principal structural subcategories, in addition to any compound containing $\geq 30\%$ fluorine by atom count, excluding hydrogen atoms. In parallel with the evolving definitions and continued refinement of the U.S. EPA's public PFAS database within the CompTox Chemicals Dashboard,[5,6] five curated PFAS structure lists reflecting successive definitional updates have been developed over the past decade. These structure inventories (PFASSTRUCT) were derived from the PubChem database, which contained more than 19.5 million fluorinated and PFAS-related compounds, of which approximately 6.1 million met the OECD structural definition of PFAS as of July 2022.[1]

The first version (PFASSTRUCTv1),[7] comprising 4,357 structures, includes compounds satisfying the following criteria: (1) molecular formulas containing 4–1000 fluorine atoms; (2) the presence of two adjacent CF_2 groups, either within a chain or ring system; (3) a fluorine-to-carbon ratio $(F/C) \geq 0.5$; and (4) exclusion of Markush structures, charged species (e.g., anions), radicals, and isotopically labeled compounds (e.g., deuterated or ^{13}C-labeled species). The second version (PFASSTRUCTv2),[8] containing 6,648 structures, reflects compounds incorporating the substructure $R-(CF_2)-C(F)(R^{'})-R^{"}$, where none of the R groups is hydrogen. This requirement ensures that both the per-fluorinated methylene ($-CF_2-$) and per-fluorinated methine ($-CF-$) carbons are fully substituted and saturated, in line with the U.S. EPA's Office of Pollution Prevention and Toxics PFAS definition, which was intended for reporting and handling of substances under the Toxic Substances Control Act in 2021. The third version (PFASSTRUCTv3),[9] comprising 8,163 structures, includes compounds containing at least one of the substructures presented in Figure S3. The fourth version (PFASSTRUCTv4),[10] which contains 10,776 structures, encompasses compounds containing all substructures shown in Figure S3, with the exception of Substructure 6 (trifluoroacetic acid), which

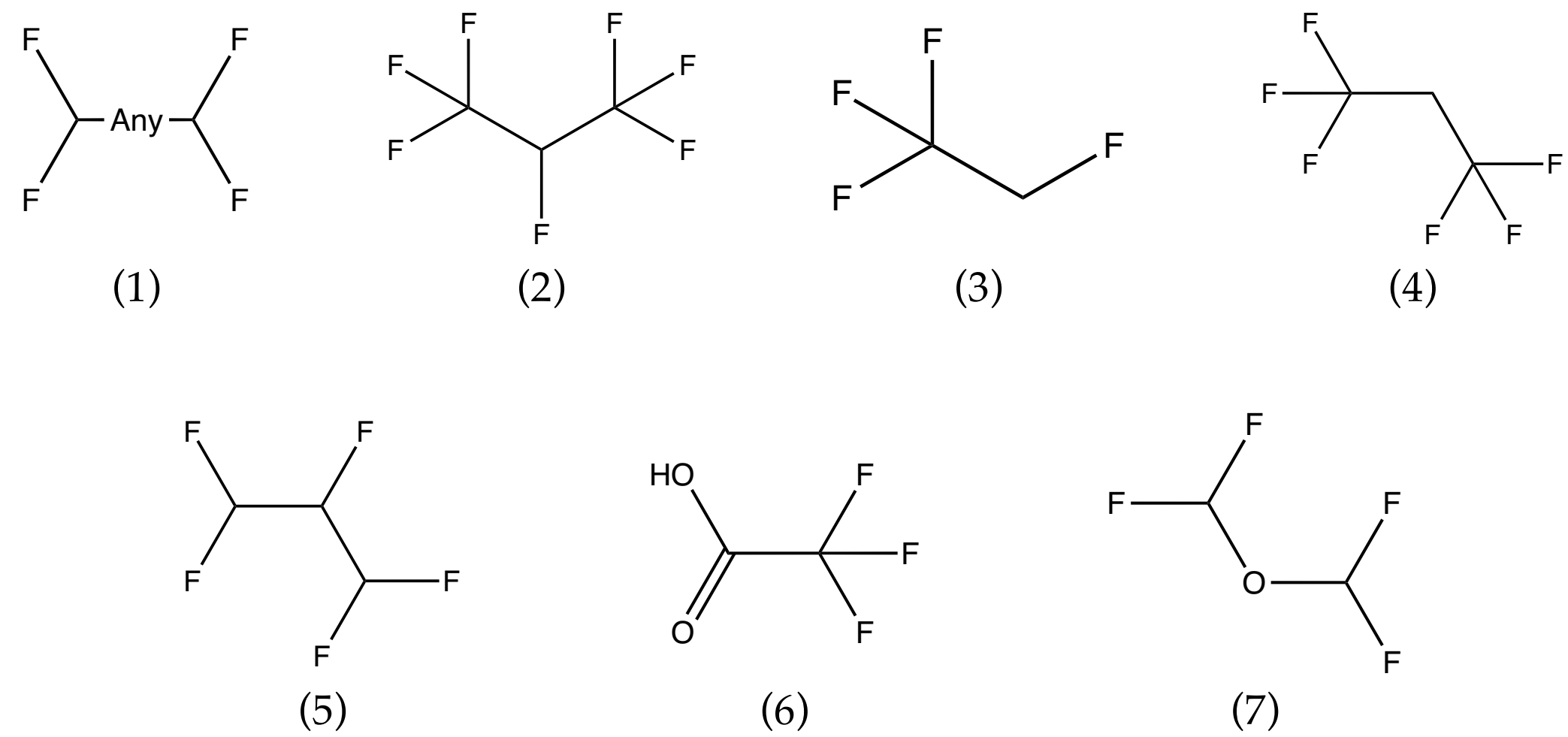

Figure S3: Substructures used for building PFASSTRUCTv3 and PFASSTRUCTv4 databases.

was excluded due to its widespread presence in common solvent systems. The changes in the size of these PFAS structural databases result from both the different PFAS definitions adopted as well as the changes in the size of the original PFAS and fluorinated compounds contained in the PubChem database. Comprehensive descriptions of the curation procedures and filtering methodologies used to assemble these PFAS structural databases are provided by Williams and co-workers.[11]

In this work, while the size of the PFAS and fluorinated compounds contained in the PubChem database as of the preparation of this manuscript (February, 2026) has increased to 23.4 million structures[12] (accessed on February 20 2026), we adopt the most recent PFAS structure database called PFASSTRUCTv5 containing 14,735 structures curated by the U.S. EPA, hosted on the CompTox Chemical Dashboard,[13] which was last updated in August 2022. This PFAS structure database contains well-structured data including their structures, molecular mass, as well as different compound identifiers such as their preferred names, Distributed Structure-Searchable Toxicity Substance Identifier (DTXSID), SMILES, and Chemical Abstracts Service Registered Number (CASRN). Gaines and co-workers[1] identified that the previous four PFASSTRUCT versions, which were solely structure-based definitions, were not robust enough to account for complex fluorinated compounds and were not easy or definite to identify. Hence, a new simplistic approach, defining PFAS as substances whose structure contains up to 30% fluorine excluding hydrogen or containing one of the four substructures depicted in Figure S4, was adopted in the curation of PFASSTRUCTv5, hereafter referred to as the EPA-identified PFAS.

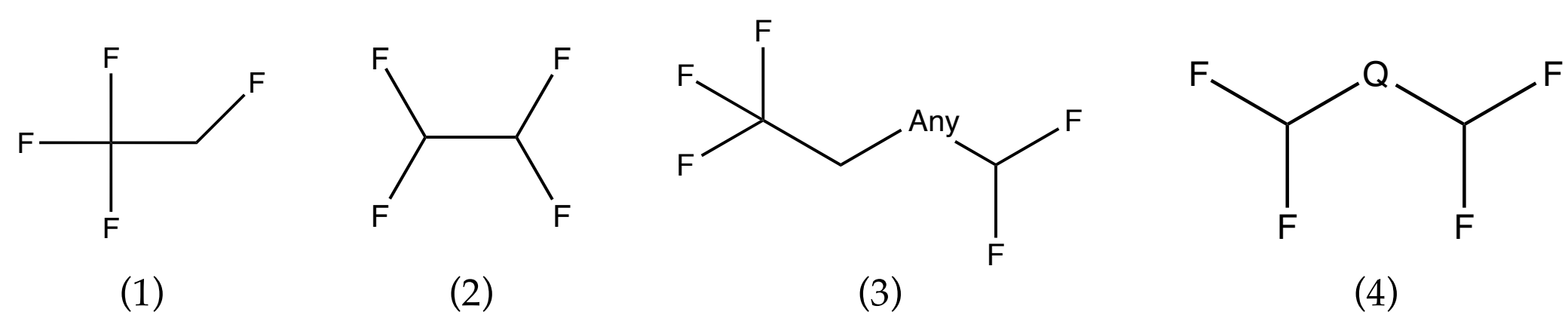

Figure S4: Substructures used in combination with the percentage fluorine by atom count, excluding hydrogen, used to curate PFASSTRUCTv5.[13] The heteroatom ”Q” can be Boron (B), Oxygen (O), Nitrogen (N), Phosphorous (P), Sulfur (S), Silicon (Si).

Text S5: PFAS data classification

After retrieving the EPA-identified PFAS data from the EPA CompTox Chemical Dashboard on the 16th of January, 2026, the data was first cleaned prior to any analysis. A total of 14,735 PFAS structures are included in the PFASSTRUCTv5 database, as shown in Figure S5.[13] The Simplified Molecular Input Line Entry System (SMILES) strings for all entries were sanitized to ensure structural validity. Of these, 14,722 structures possessed valid SMILES representations, while 13 were determined to be invalid and were excluded from further analyses. The validated PFAS structures were subsequently classified into ionic (1,989) and non-ionic (12,733) categories. The ionic species were further subdivided into anionic (net negative charge; 253), cationic (net positive charge; 330), and zwitterionic (overall neutral with internally balanced charges; 1,406) compounds. Representative examples of these categories are provided in Figure S6. Ionic PFAS were also categorized based on structural connectivity as either whole structures (1,060) or disjointed structures (929), with illustrative examples shown in Figure S7. Similarly, the non-ionic (neutral) PFAS structures were classified as whole (12,479) or disjointed (254), with representative structures presented in Figure S8. All PFAS entries with valid SMILES strings were retained for subsequent analyses.

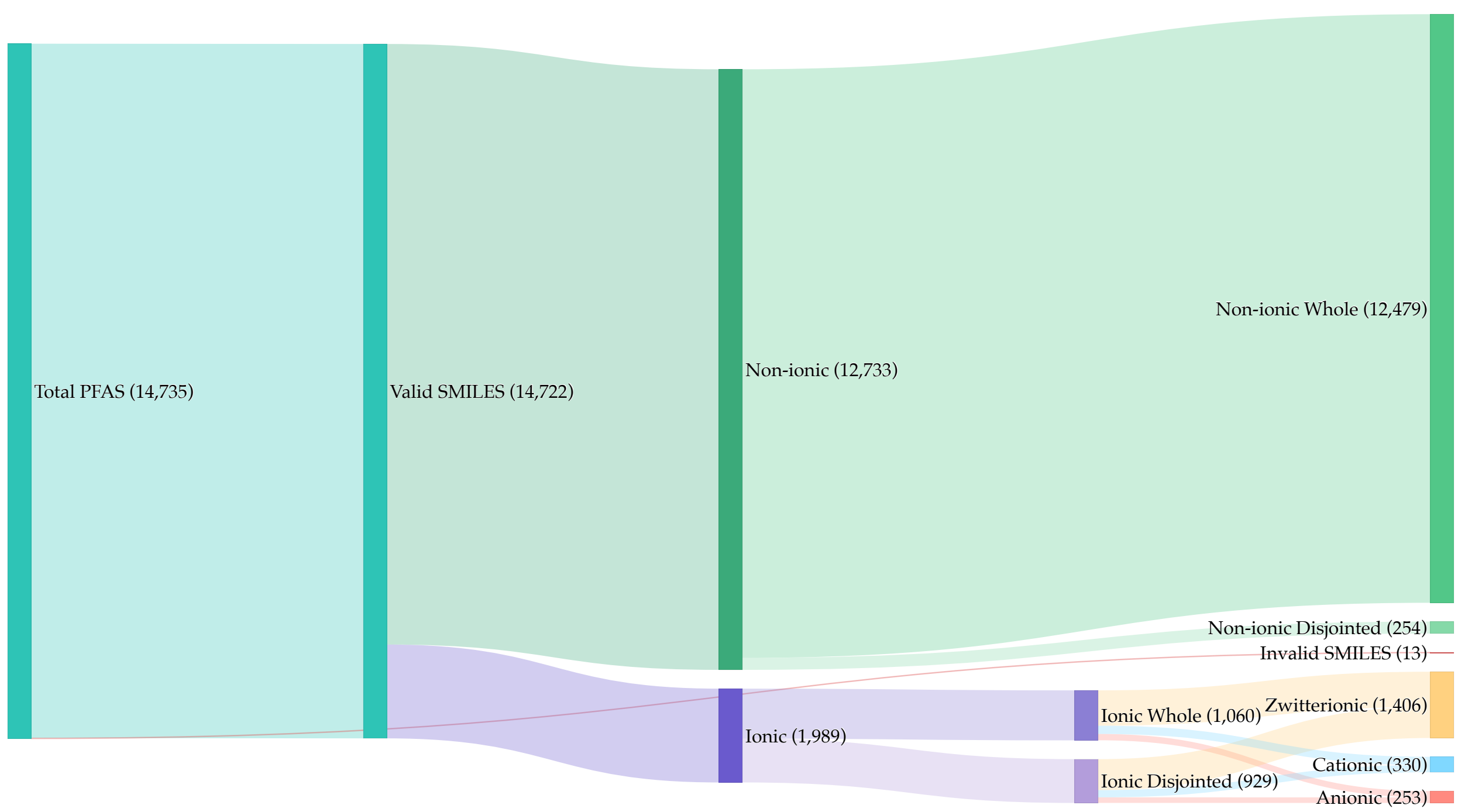

Figure S5: Sankey diagram showing the structural classification of PFAS included in the PFASSTRUCTv5 database. The number of structures within each class is indicated in parentheses.

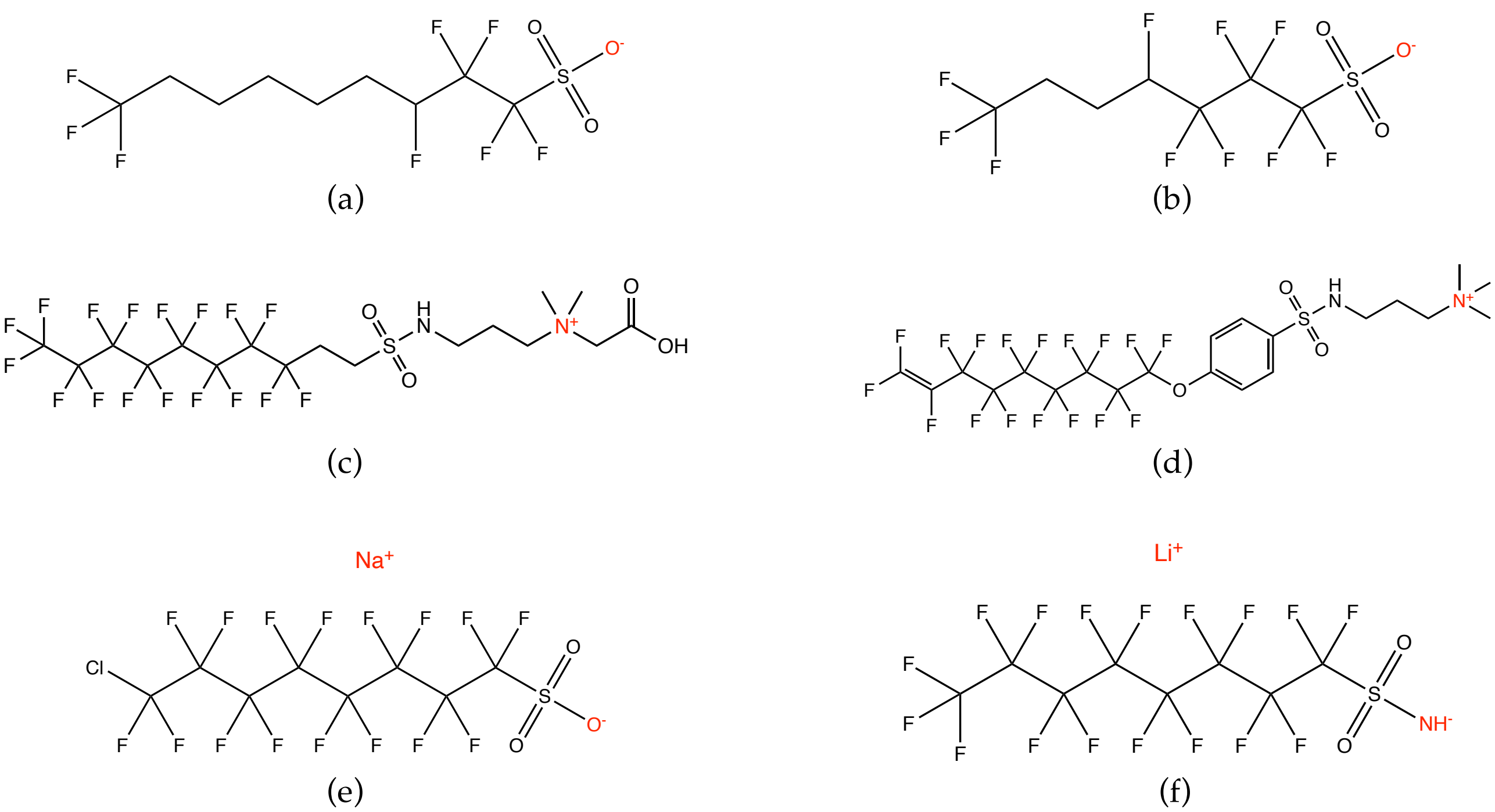

Figure S6: Examples of anionic (a&b), cationic (c&d), and zwitterionic (e&f) PFAS structures contained in the PFASSTRUCTv5 database. (a) 1,1,2,2,3,9,9,9-Octafluorononane-1-sulfonate, (b) 1,1,2,2,3,3,4,7,7,7-Decafluoroheptane-1-sulfonate, (c) N-(Carboxymethyl)-N,N-dimethyl-3-(1H,1H,2H,2H-perfluoro-1-decanesulfonamido)propan-1-aminium, (d) 3-[(4-[(1,1,2,2,3,3,4,4,5,5,6,6,7,7,8,9,9-Heptadecafluoro-8-nonen-1-yl)oxy]phenylsulfonyl)amino]-N,N,N-trimethyl-1-propanaminium, (e) Sodium 8-chloroperfluoro-1-octanesulfonate, (f) Perfluorooctanesulfonamide lithium salt (1:1).

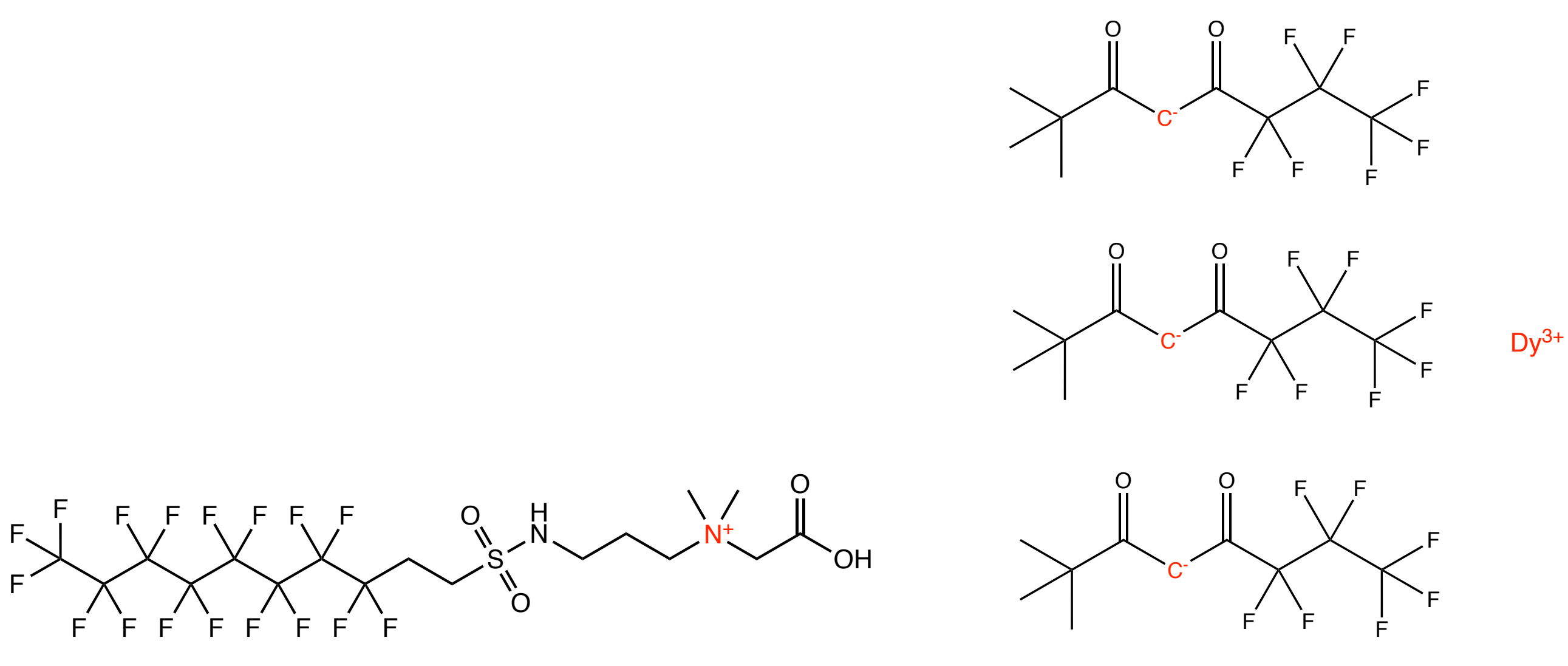

(a) (b)

Figure S7: Examples of (a) ionic PFAS whole (N-(Carboxymethyl)-N,N-dimethyl-3-(1H,1H,2H,2H-perfluoro-1-decanesulfonamido)propan-1-aminium) and (b) ionic PFAS disjointed (Tris(perfluorobutanoylpivaloylmethano)dysprosium) structures.

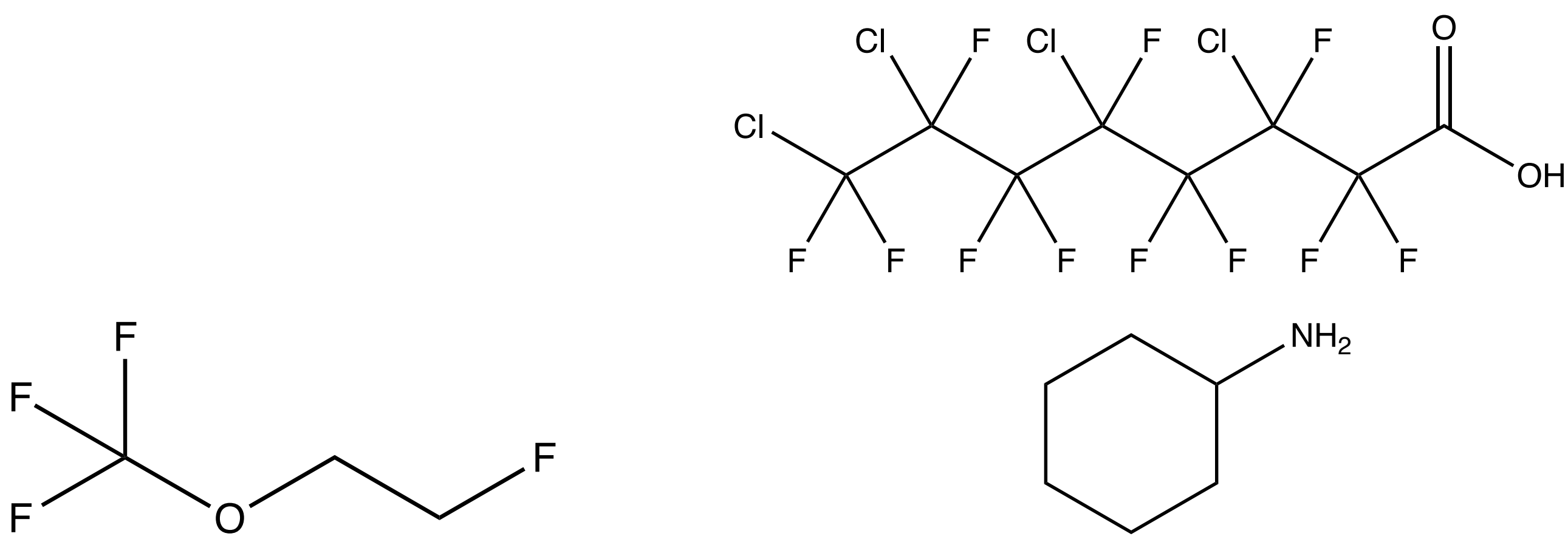

(a) (b)

Figure S8: Examples of (a) whole non-ionic PFAS (1-Fluoro-2-(trifluoromethoxy)ethane) and (b) disjointed non-ionic PFAS (3,5,7,8-Tetrachloro-2,2,3,4,4,5,6,6,7,8,8-undecafluorooctanoic acid–cyclohexanamine (1/1)) structures.

Text S6: Molecular simulation approach for polymer modeling

The generation of the required screening charge densities for the polymers studied in this work can be summarized in three steps:[14,15] (1) perform a molecular dynamic (MD) simulation of a single oligomer of the desired polymer in a dilute solvent to obtain simulation trajectories that sample a wide range of chain conformations that are representative of the behavior of the actual polymer in a real solvent system, (2) select a representative subset of conformers from the saved MD trajectories to span a wide range of representative oligomers, and (3) subject the selected oligomers to DFT calculations, producing cosmo files which stores important thermodynamic quantities including the chemical potential and surface screening charges of each conformer. While all screening charge densities in this work were obtained from our previously published studies,[14,15] this section provides details on the methods used to generate them.

Polymers were modeled as oligomers and simulated in dilute solution. All atomistic MD simulations were performed in the isothermal–isobaric (NPT) ensemble using GROMACS 2016. Each simulation system consisted of one oligomer molecule and 216 solvent molecules. Solvents were selected based on literature reports of good solvents for each polymer: PE, PP, and PS were simulated in toluene, whereas PVC and PET were simulated in dichloromethane. Molecular parameterization was performed using Antechamber with the Generalized AMBER Force Field (GAFF). Systems were initialized in cubic simulation boxes containing a single polymer chain, solvated, energy minimized, and equilibrated for 2 ns in the NPT ensemble at 300 K and 1 bar using a velocity-rescale thermostat and Berendsen barostat. Production simulations of 10 ns were subsequently performed under NPT conditions using the Nosé–Hoover thermostat and Parrinello–Rahman barostat. All simulations employed a leapfrog integrator with a 2 fs timestep. A 1.2 nm cutoff was used for neighbor lists. van der Waals interactions were described using a shifted Lennard–Jones potential with a smooth truncation at 1.2 nm. Electrostatic interactions were computed using the smooth Particle Mesh Ewald (PME) method with a 1.2 nm short-range cutoff, 0.14 nm grid spacing, and fourth-order interpolation. All bonds were constrained using the LINCS algorithm. Thermostat and barostat coupling constants were set to 2.0 ps, with an isothermal compressibility of 3.0×10^{-5} bar^{-1}.

Representative oligomer conformations (hereafter referred to as conformers) were extracted from the MD trajectories based on two structural descriptors: the radius of gyration (R_g) and the solvent-accessible surface area (SASA). To ensure broad conformational coverage, a two-dimensional R_g–SASA scatter plot was partitioned using a square grid, and conformers closest to grid intersection points were selected. The selected conformers were subjected to density functional theory (DFT) calculations to generate COSMO files containing screening charge densities. Geometry optimizations were performed in implicit water using the conductor-like polarizable continuum model (CPCM), followed by single-point calculations in the infinite dielectric constant limit. All DFT calculations were carried out using Gaussian 16 at the BVP86/TZVP/DGA1 level of theory.

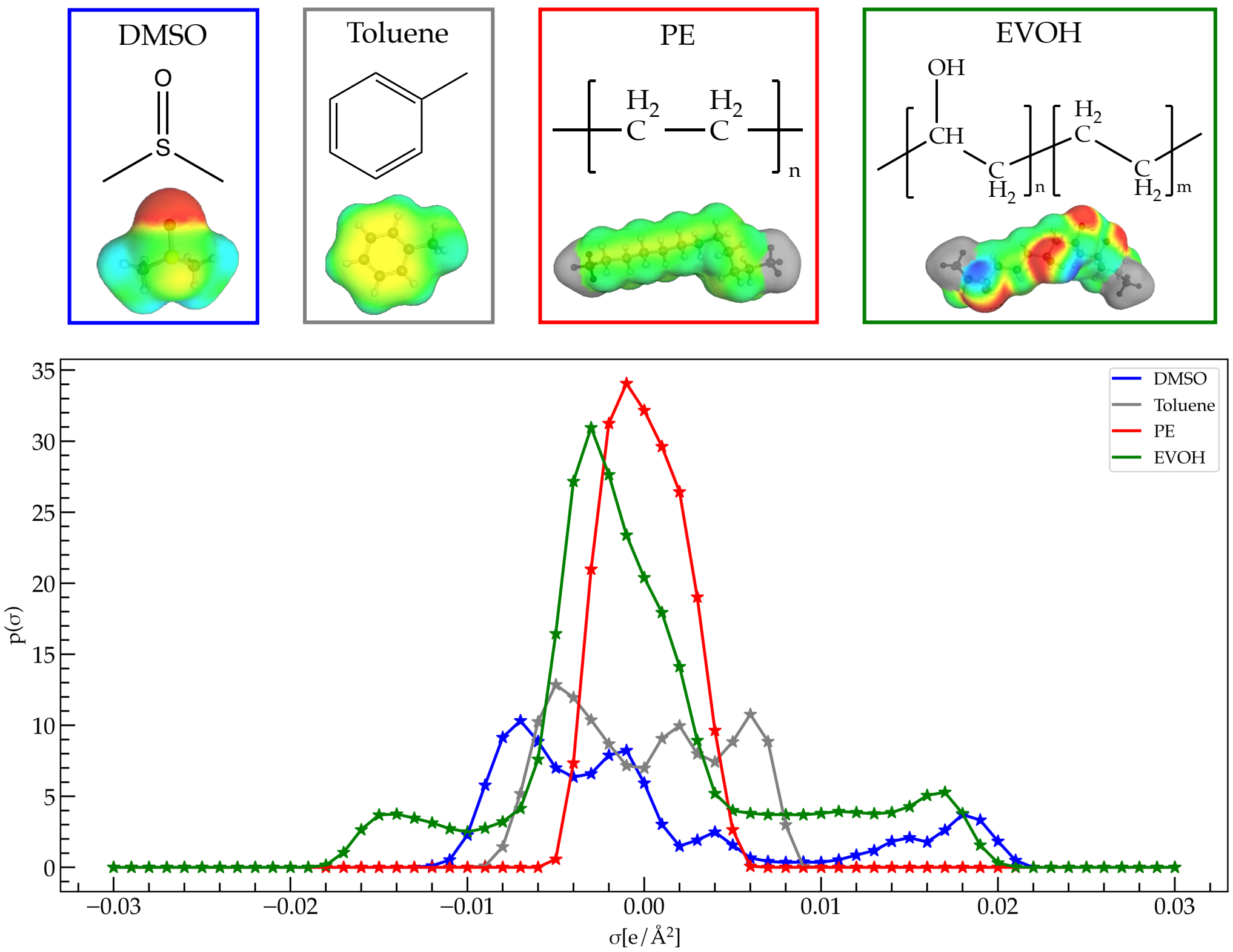

Figure S9: Screening charge densities and corresponding $\sigma-$profiles of representative solvents: dimethyl sulfoxide (DMSO) and toluene, and polymers: polyethylene (PE) and ethylene vinyl alcohol (EVOH) used in the similarity threshold analysis. The topological difference between their $\sigma-$profiles highlights their chemical uniqueness.

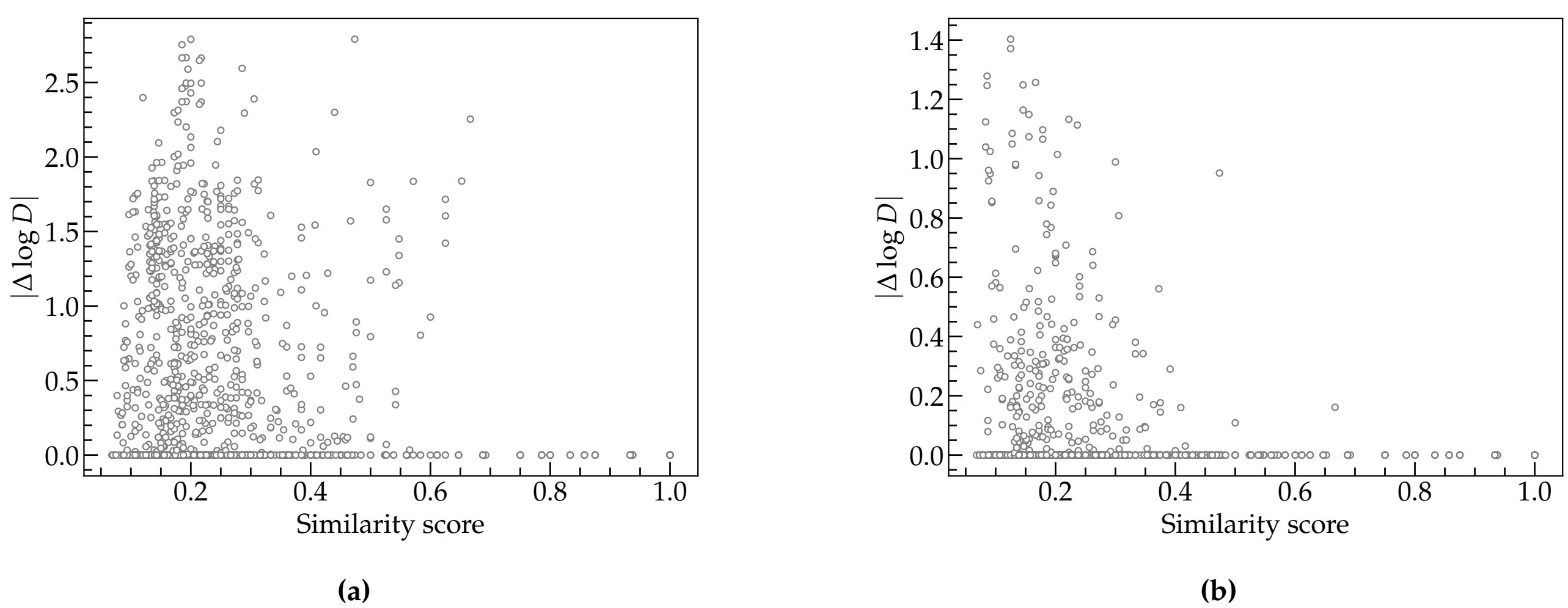

Figure S10: Similarity threshold analysis across polymer–solvent systems: (a) EVOH–DMSO system, and (b) PE–toluene system.

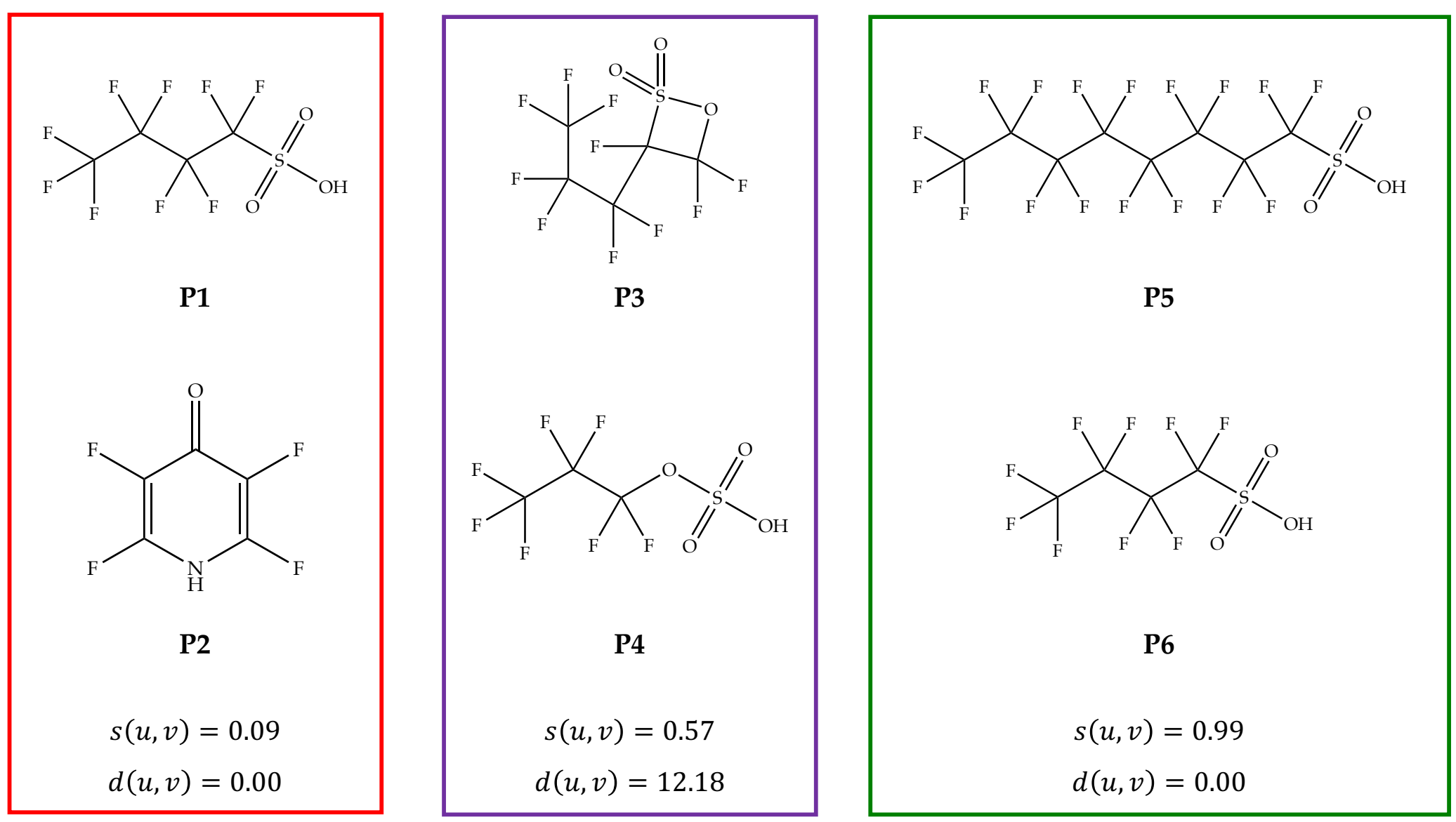

Figure S11: Examples of PFAS pairs with different Tanimoto similarity scores and their absolute $\log D$ difference. Perfluorobutanesulfonic acid (**P1&P6**, $\log D = 6.64$), 2,3,5,6-tetrafluoropyridin-4-ol (**P2**, $\log D = 6.37$), 3,4,4-Trifluoro-3-(heptafluoropropyl)-1,2λ^6-oxathietane-2,2-dione (**P3**, $\log D = -0.22$), perfluoropropyl hydrogen sulfate (**P4**, $\log D = 7.96$), and perfluorooctanesulfonic acid (**P5**, $\log D = 6.36$). The color of each box corresponds to the cloud points highlighted in Figure 7a of the main text. $s(u,v)$ and $d(u,v)$ corresponds to the similarity score and absolute $\log D$ difference between any PFAS pair.

Text S7: t-distributed stochastic neighbor embedding (t-SNE), K-means, and elbow method

t-distributed stochastic neighbor embedding (t-SNE) is a non-linear dimensionality reduction technique developed to visualize high-dimensional data in 2-D or 3-D. At its core, t-SNE transforms pairwise distances in the original high-dimensional space into conditional probabilities that quantify similarity between data points.[16] Specifically, in the high-dimensional space, the conditional probability $p_{j|i}$ (see eq S5) that a data point x_j is a neighbor of x_i is modeled using a Gaussian (normal) distribution centered at x_i. Here, x_i and x_j denote arbitrary data points in the dataset, $\sum_{k \neq i}$ represents the normalization over all distinct points, and σ_i corresponds to the standard deviation of the Gaussian centered at x_i, which governs the local neighborhood scale around that point.

$$p_{j|i} = \frac{\exp\left(- \|x_i - x_j\|^2 / 2\sigma_i^2\right)}{\sum_{k \neq i} \exp\left(- \|x_i - x_k\|^2 / 2\sigma_i^2\right)} \tag{S5}$$

After computing the conditional probabilities in the high-dimensional space, an analogous set of joint probabilities, q_{ij} (see eq S6), is defined in the low-dimensional embedding space (2-D or 3-D). In contrast to the Gaussian kernel used in the high-dimensional space, t-SNE employs a heavy-tailed Student's t-distribution to model pairwise similarities in the embedding. Here, y_i and y_j denote the low-dimensional coordinates of data points i and j, respectively, and the normalization term $\sum_{k \neq l}$ accounts for the summed similarities over all distinct point pairs in the map. To ensure that the low-dimensional distribution (Q) closely represents the data structure in the original high-dimensional space (P), t-SNE minimizes a cost function C (see eq S7) defined as the Kullback–Leibler (KL) divergence between P and Q. This optimization procedure iteratively adjusts the embedding coordinates to preserve local neighborhood relationships while mitigating crowding effects in the reduced space.[17]

$$q_{ij} = \frac{\left(1 + \|y_i - y_j\|^2\right)^{-1}}{\sum_{k \neq l} \left(1 + \|y_k - y_l\|^2\right)^{-1}} \tag{S6}$$

$$C = KL(P \,||\, Q) = \sum_i \sum_j p_{ij} \log \frac{p_{ij}}{q_{ij}} \tag{S7}$$

K-means is a centroid-based iterative algorithm developed to partition cloud points into k distinct, non-overlapping clusters. This algorithm seeks to make cloud points within a cluster as similar as possible while ensuring points in different clusters are as far apart as possible. K-mean algorithm is formulated as an optimization problem that seeks to minimize the *Within-Cluster Sum of Squares (WCSS)* often referred to as *inertia* (see eq S8);[18] where k is an input variable that pre-specifies the number of cluster, S_i is the set of points in the ith cluster, μ_i is the centroid (or mean) of all points in S_i, and $\|x - \mu_i\|^2$ computes the squared Euclidean distance between a point and its assigned centroid. In detail, the algorithm begins by carefully picking the specified k points in a latent space and assigning them as the centroids.[19] Each data point x is then assigned to clusters whose centroid is the closest, typically measured using the squared Euclidean distance. Once each point x is assigned to a

cluster, new centroids are calculated as the arithmetic mean of all points contained in a cluster. These steps are repeated until either the centroids no longer significantly move, the assignment of points to clusters no longer changes, or the maximum number of iterations is met.[20]

$$J = \sum_{i=1}^{k} \sum_{x \in S_i} \|x - \mu_i\|^2 \tag{S8}$$

Selecting an appropriate value for k, i.e., the number of clusters, is as important as the application of the k-means algorithm itself. In this study, the *Elbow method* was employed to determine the optimal number of clusters.[21] Examining the within-cluster sum of squares (WCSS), denoted by J in eq S8, reveals that increasing the number of clusters (k) generally leads to a reduction in WCSS. For example, consider a dataset containing 1000 points in a high-dimensional space. Specifying $k = 1$ (a single cluster) would yield a relatively large WCSS, whereas specifying $k = 1000$ (each point forming its own cluster) would produce $J = 0$. Consequently, plotting WCSS (J) against the number of clusters (k) typically yields a curve resembling a bent arm. As k increases, WCSS decreases rapidly at first, followed by a region where the rate of decrease becomes more gradual. The point at which this transition occurs, forming an "elbow" in the curve, is commonly interpreted as the optimal value of k, as further increases in k beyond this point result in only marginal reductions in WCSS.

Text S8: Structural analyses of the identified PFAS clusters obtained from t-SNE

As shown in Figure 8 of the main text, eight PFAS families were identified based on structural similarity. The most prevalent substructures within each cluster are summarized in Figure S12. Notably, 100% of the PFAS structures in every cluster contain at least one fluorine atom (F, bit 42), consistent with the defining characteristic of a PFAS.

In Cluster 1 (Figure S12a), all PFAS structures contain an oxygen atom (O, bit 164). Additionally, 77% of the structures exhibit a cyclic substructure (bit 163), and 48% contain a carboxylic group ($C = O$, bit 154). Cluster 2 (Figure S12b) is characterized by the universal presence of sulfonate or sulfonic functional groups (bit 73 and bit 60), along with nitrogen atoms (bit 161), indicating the occurrence of functional groups such as sulfinylamines. Cluster 3 (Figure S12c) is primarily composed of compounds containing cyclic or aromatic rings (bit 163), although no single dominant functional group distinguishes this cluster. In Cluster 4 (Figure S12d), all PFAS structures also contain oxygen atoms (O, bit 164), and a substantial fraction includes carboxylic or ester functionalities ($-O-$, $C = O$). However, in contrast to Cluster 1, the majority of compounds in this cluster are predominantly aliphatic. Cluster 5 (Figure S12e) is characterized by the universal presence of nitrogen atoms (N, bit 161), corresponding to amine ($-NH$) and amino ($-NH_2$) functional groups. Furthermore, 85% of the structures contain cyclic rings, and 60% contain oxygen atoms, suggesting the prevalence of amide functionalities within this cluster. Cluster 6 (Figure S12f), similar to Cluster 3, consists of fluorinated compounds without a clearly dominant functional group, although the structures are primarily aliphatic. Cluster 7 (Figure S12g) shares similarities with Cluster 5, containing amine, amino, and amide functional groups; however, the structures are predominantly aliphatic rather than cyclic. Finally, Cluster 8 (Figure S12h) resembles Cluster 2 in that it contains sulfonate or sulfonic functional groups, but in contrast, nitrogen atoms are absent from the structures in this cluster. A summary of the key functional groups defining each cluster, along with examples of PFAS structures from each cluster, is provided in Table S1 of the main text.

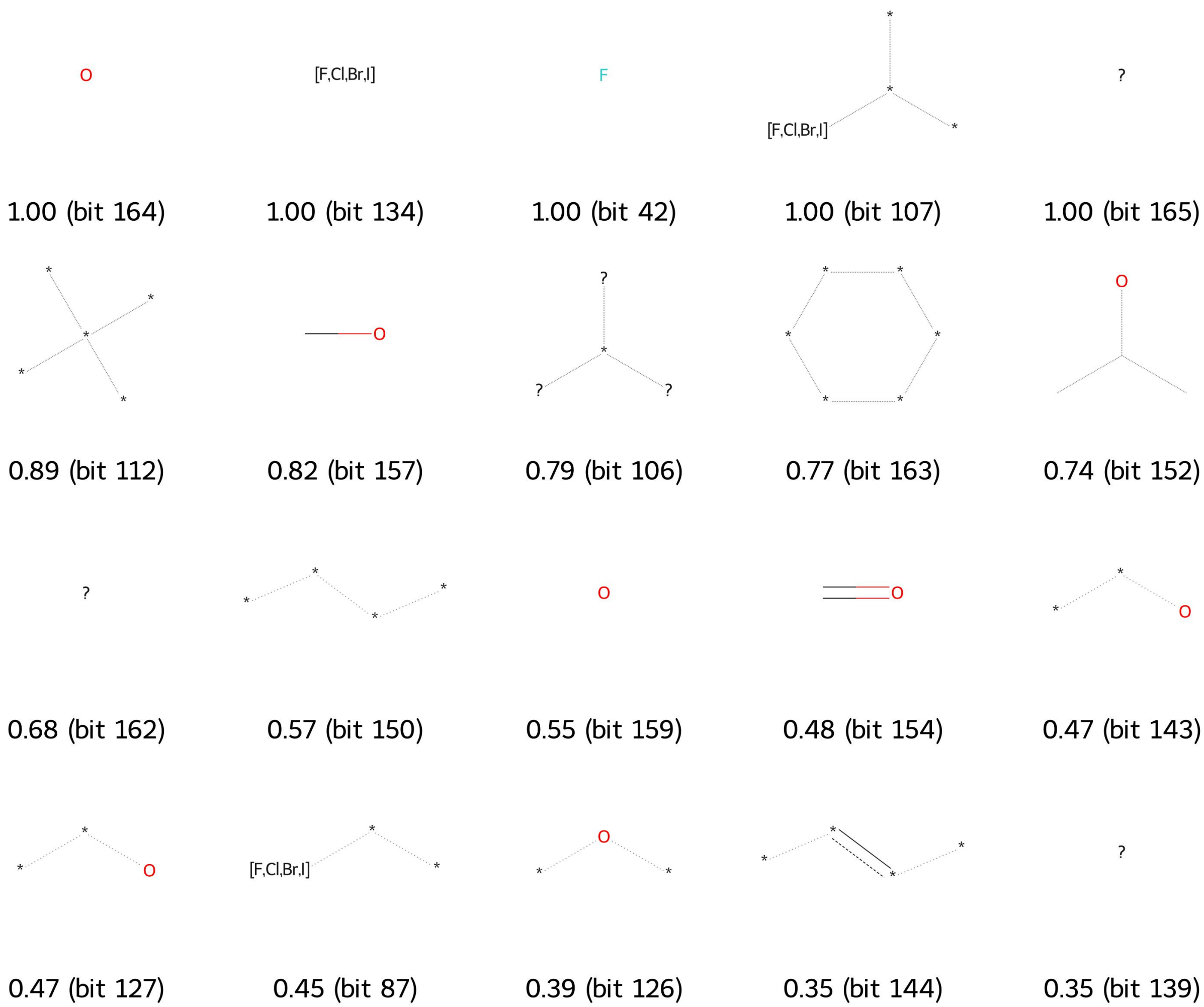

(a)

Figure S12: Top 20 most common substructures based on the MACCS keys contained in (a) cluster 1, (b) cluster 2, (c) cluster 3, (d) cluster 4, (e) cluster 5, (f) cluster 6, (g) cluster 7, and (h) cluster 8. The numbers (outside the brackets) depict the fraction of PFAS structures in the clusters having the specified substructure, while the bit number in brackets corresponds to the bit that encodes the substructure based on the MACCS keys.

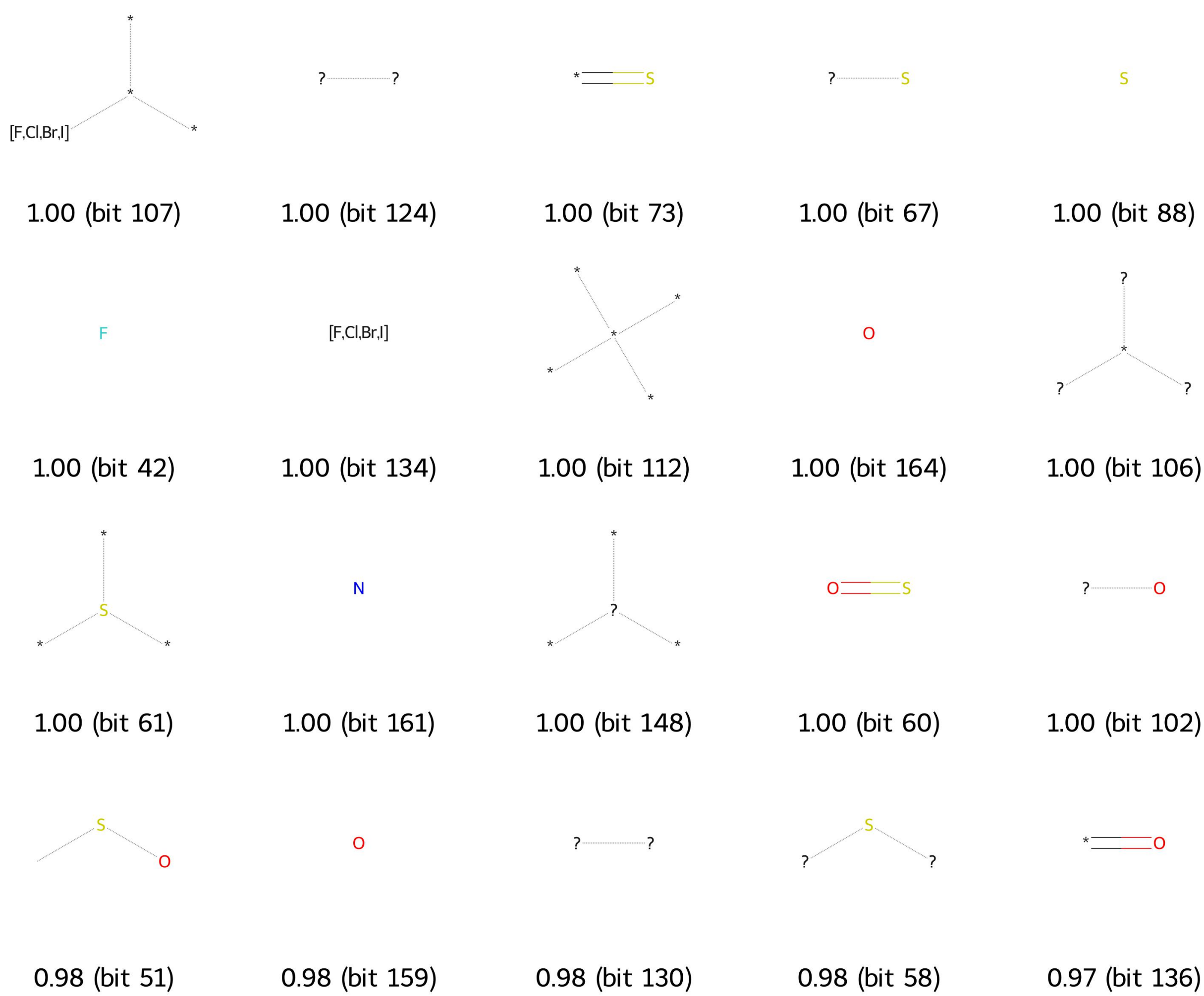

(b)

Figure S12: (continued)

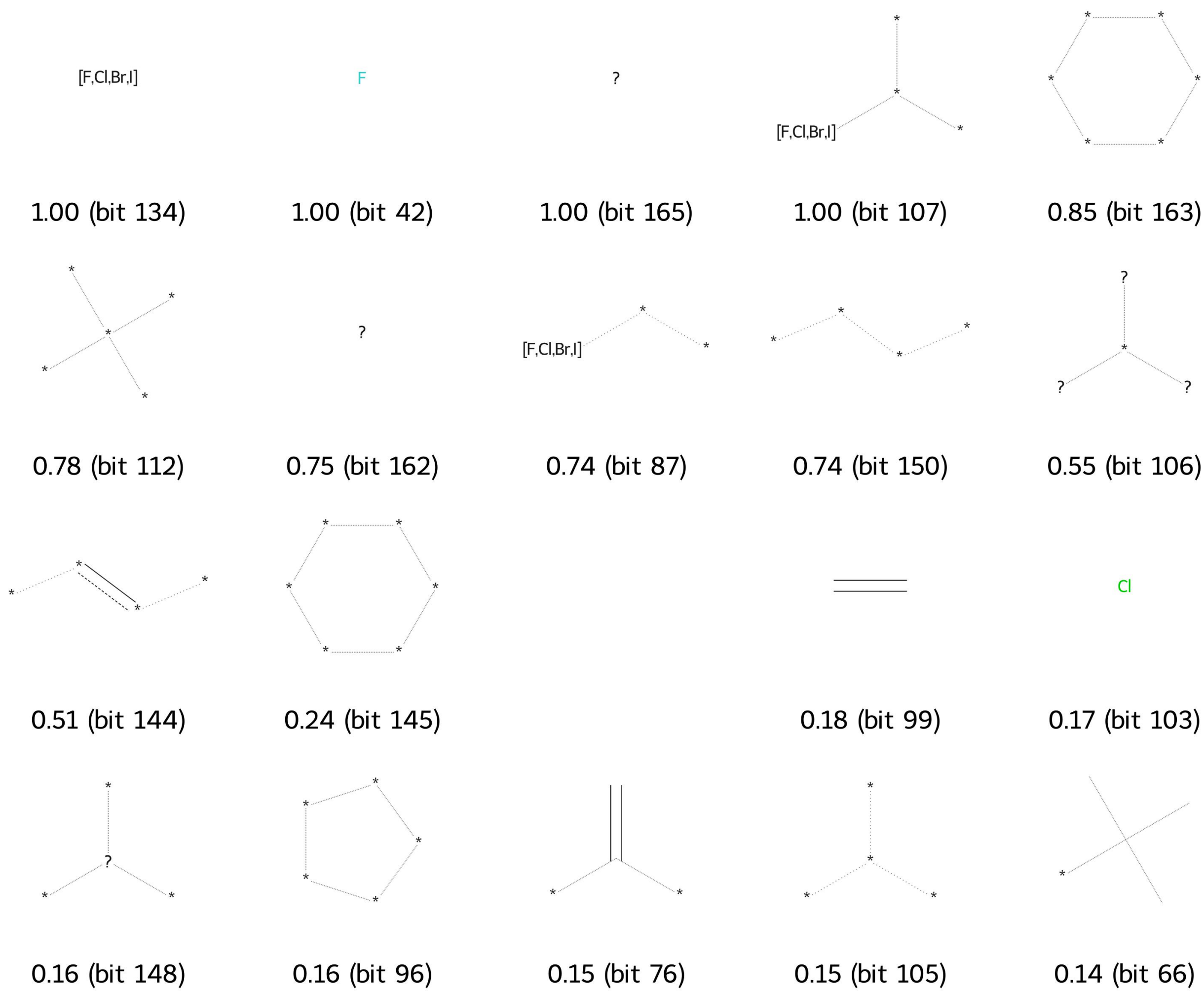

(c)

Figure S12: (continued)

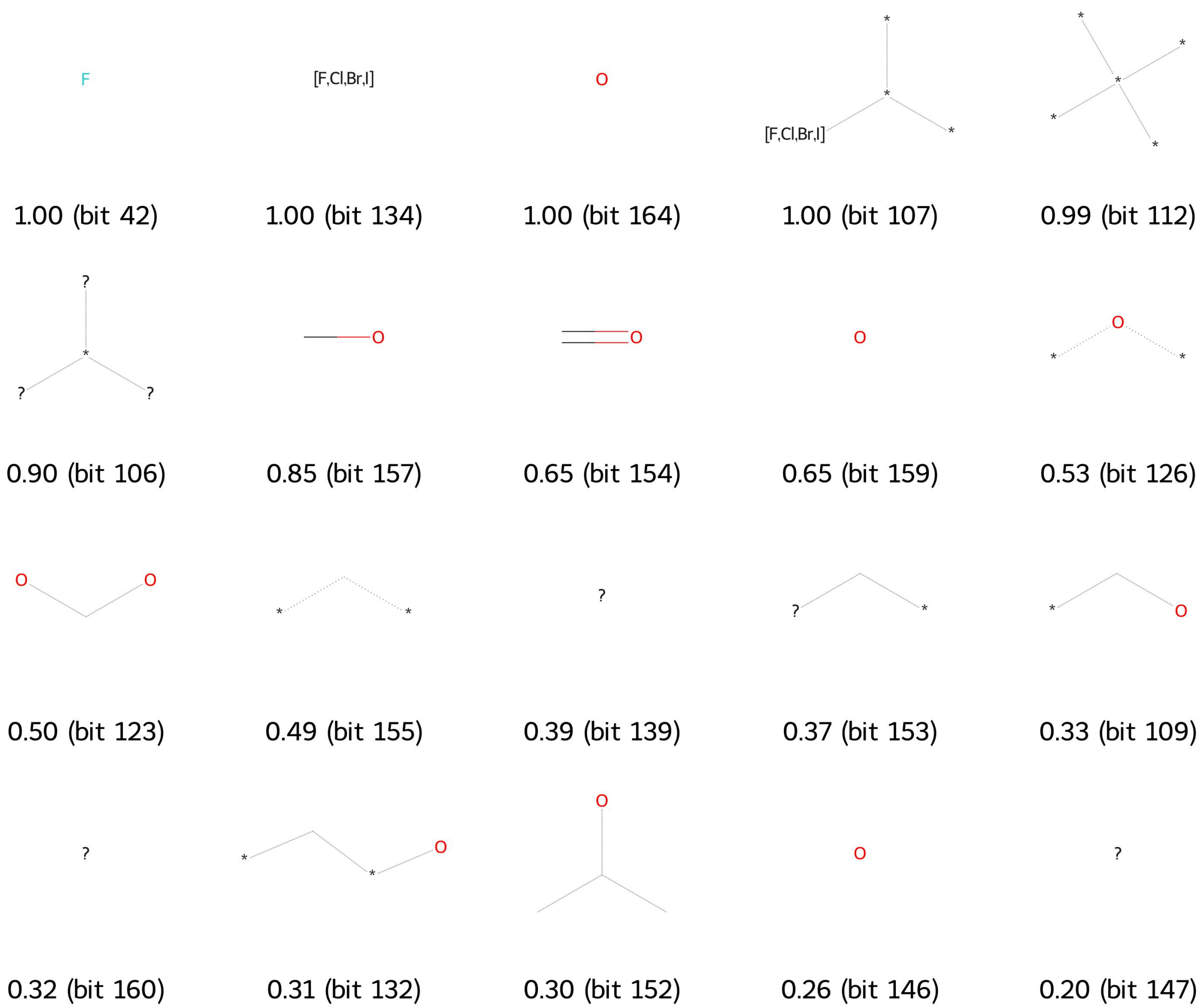

(d)

Figure S12: (continued)

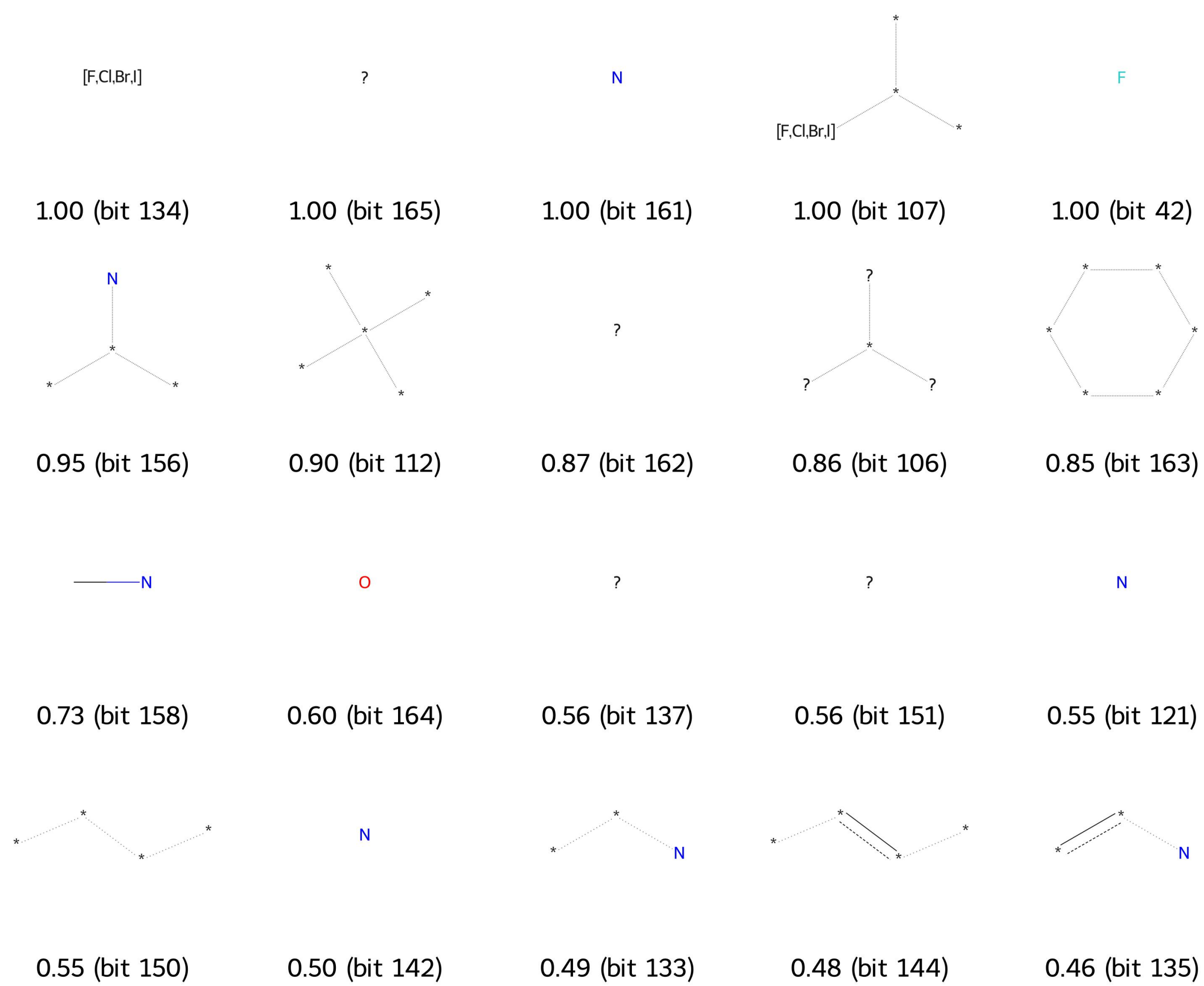

(e)

Figure S12: (continued)

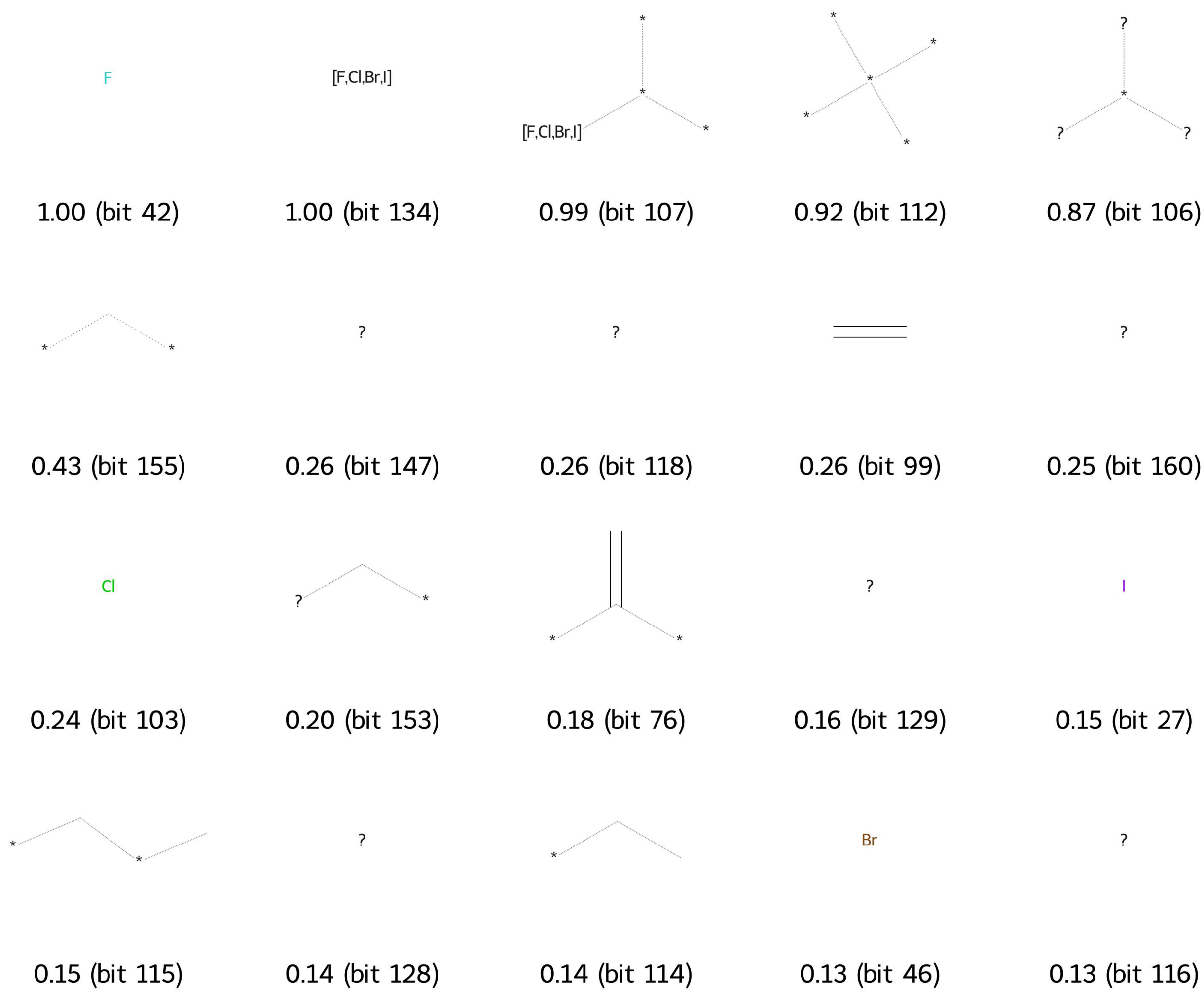

(f)

Figure S12: (continued)

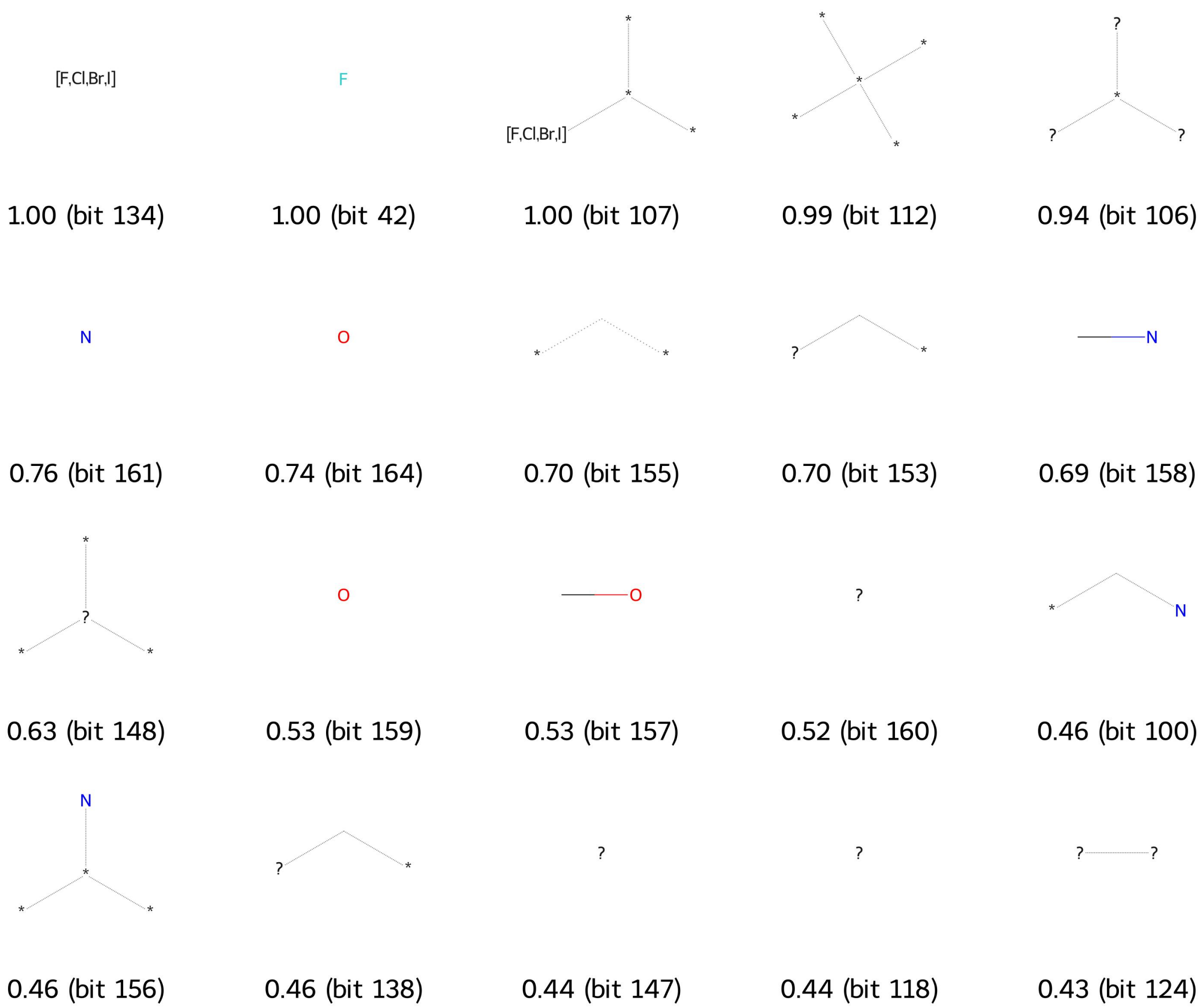

(g)

Figure S12: (continued)

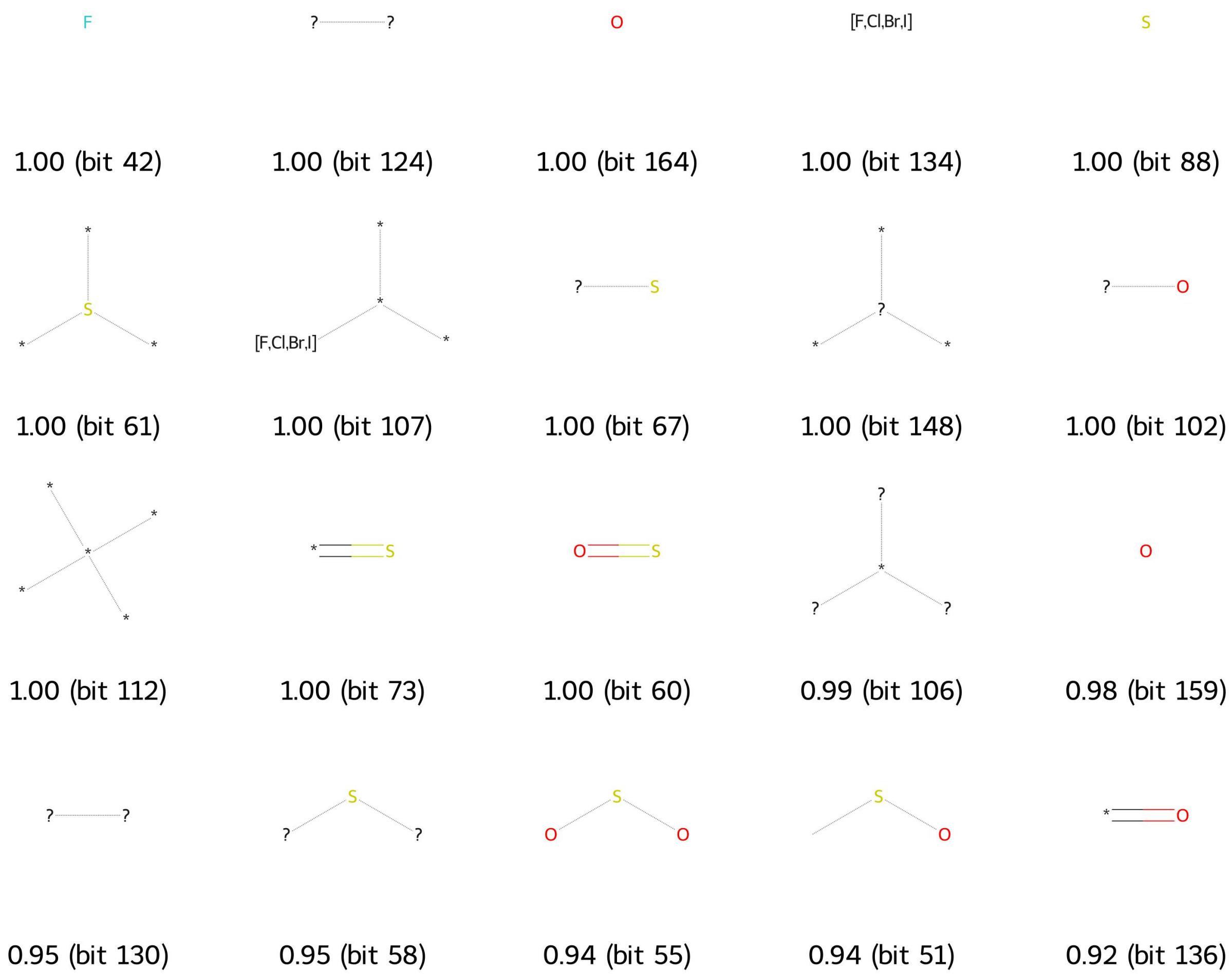

(h)

Figure S12: (continued)

Table S1: A summary of the key structural features of the eight identified PFAS families obtained via t-SNE (see Figure 8a of the main text).

Cluster no. (size)	Key structural features	PFAS structure examples
1 (1,678)	Cyclic, $-O-$, $C=O$	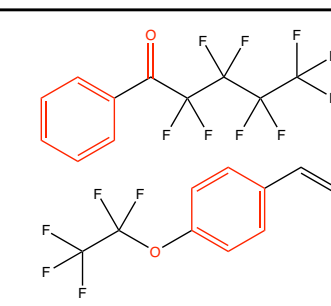
2 (1,277)	Sulfinyl and amide ($-N-S=O$)	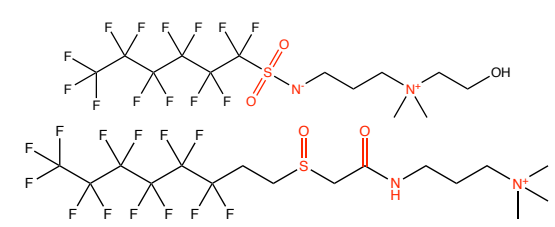
3 (1,378)	Cyclic fluorinated	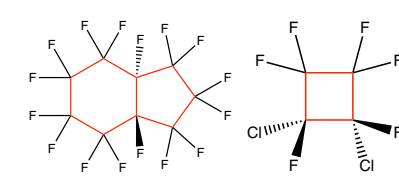
4 (3,171)	Aliphatic, $-O-$, $C=O$	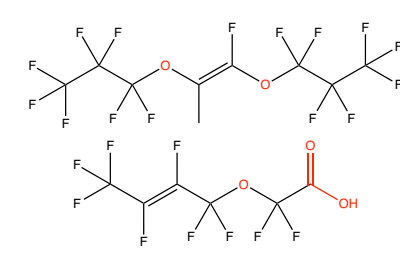
5 (2,123)	Cyclic, $-N$, NH, NH_2, $-O-$	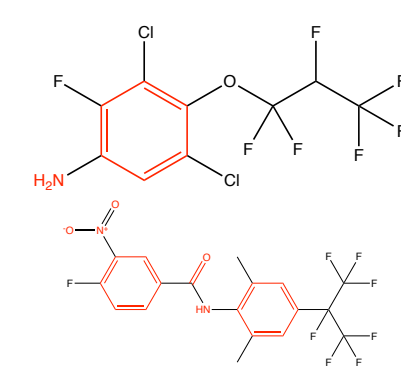
6 (2,063)	Aliphatic fluorinated	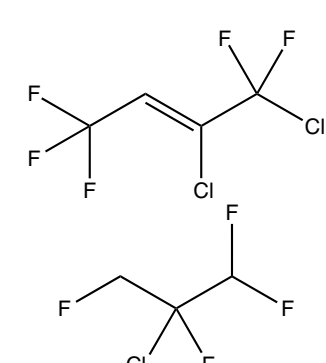
7 (1,919)	Aliphatic $-N$, NH, NH_2, $-O-$	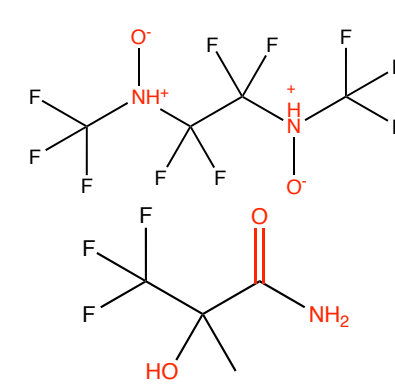
8 (1,113)	Sulfonic/sulfonate	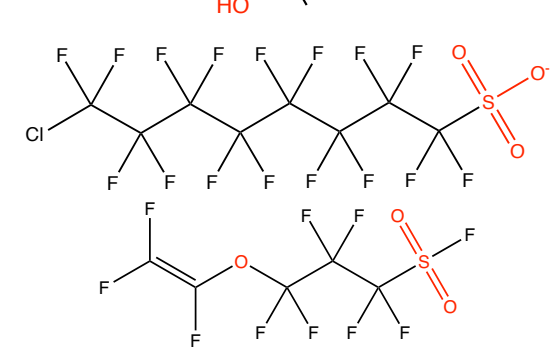

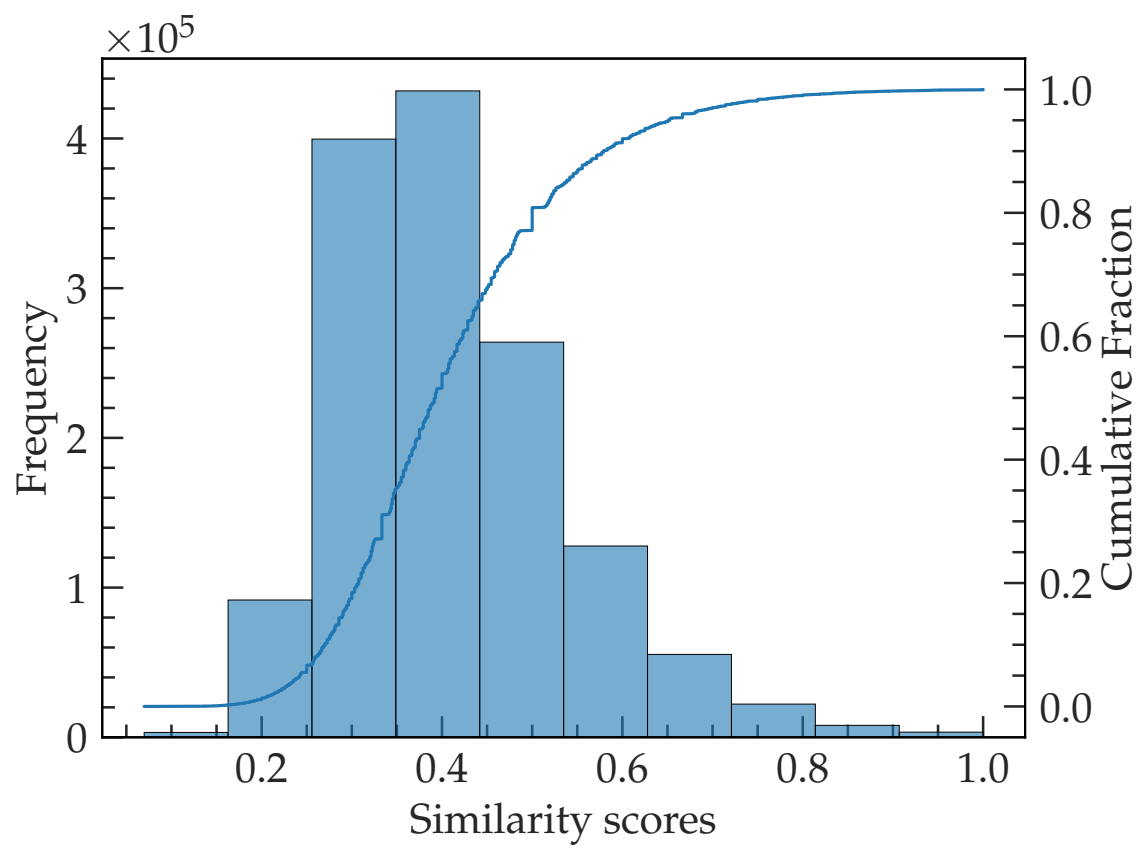

(a)

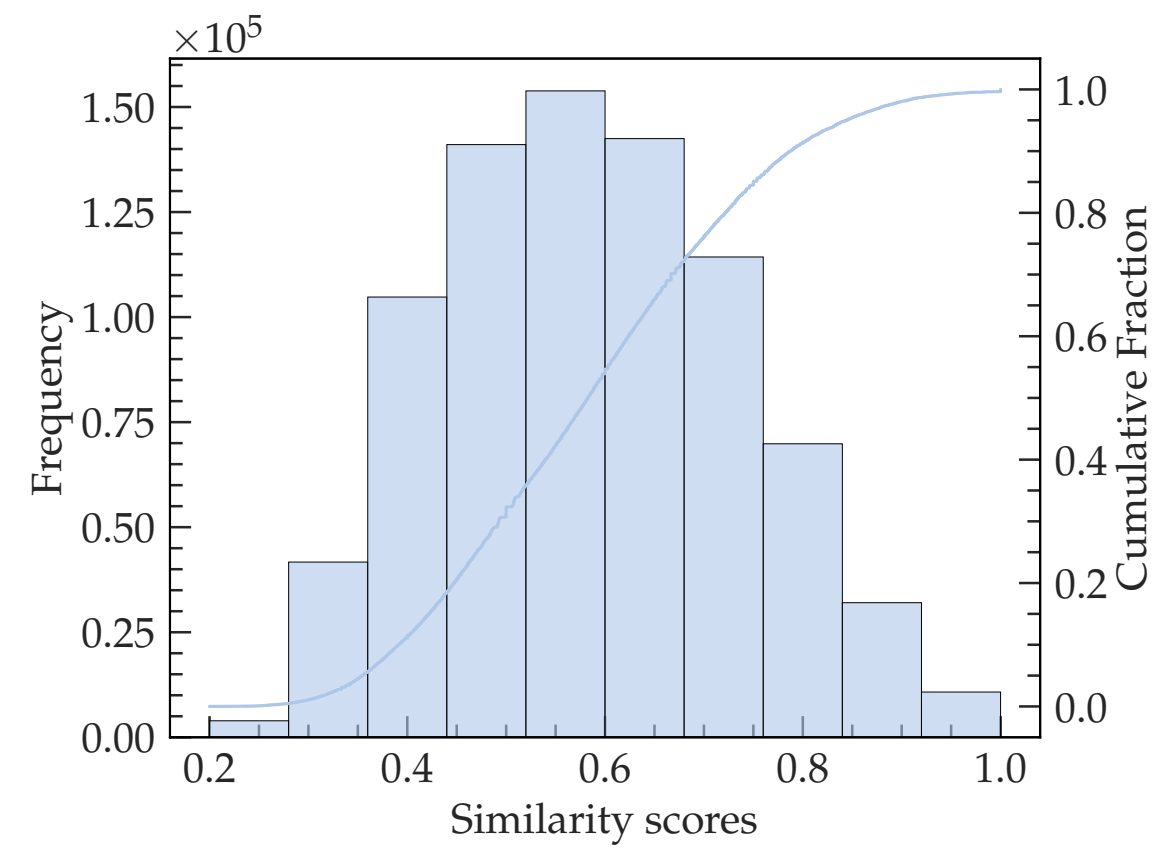

(b)

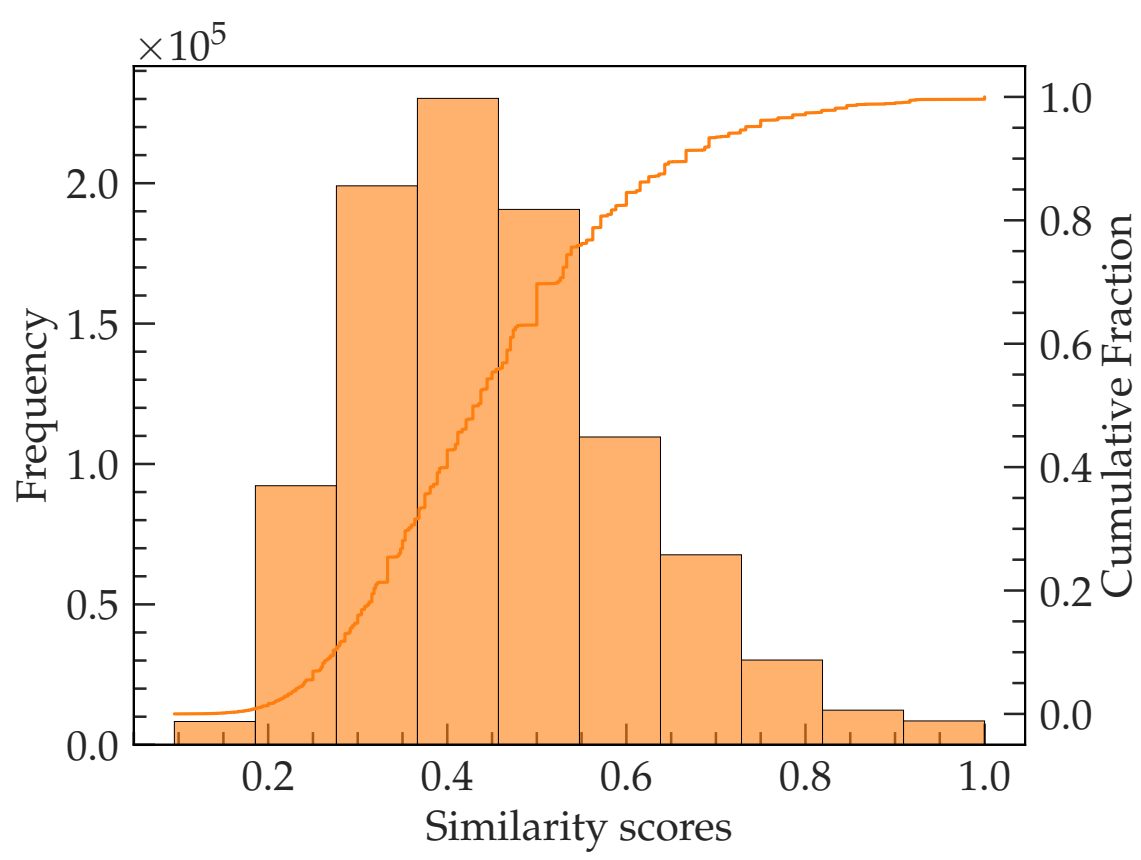

(c)

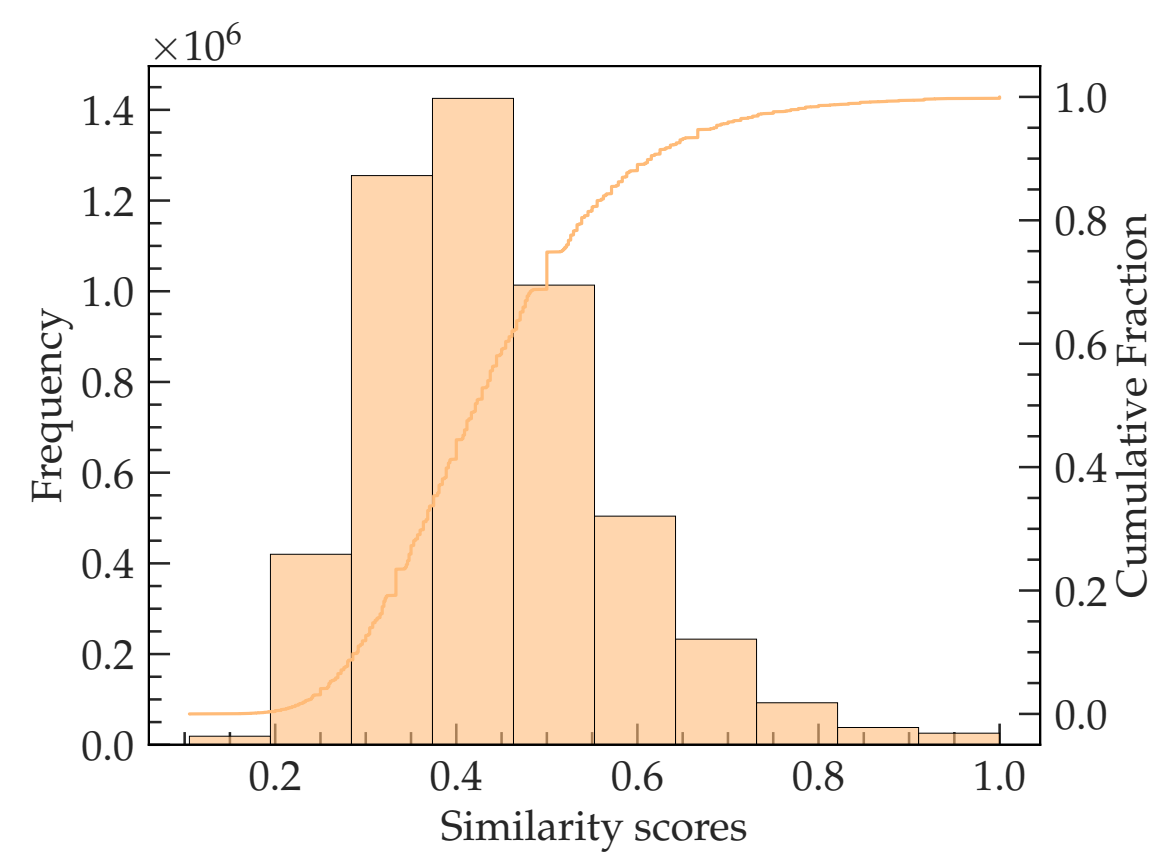

(d)

Figure S13: Distribution of pairwise PFAS structural similarity within each identified cluster: (a) Cluster 1, (b) Cluster 2, (c) Cluster 3, (d) Cluster 4, (e) Cluster 5, (f) Cluster 6, (g) Cluster 7, and (h) Cluster 8. Colors in the distribution plots are consistent with the PFAS cluster color scheme used in Figure 8 of the main text.

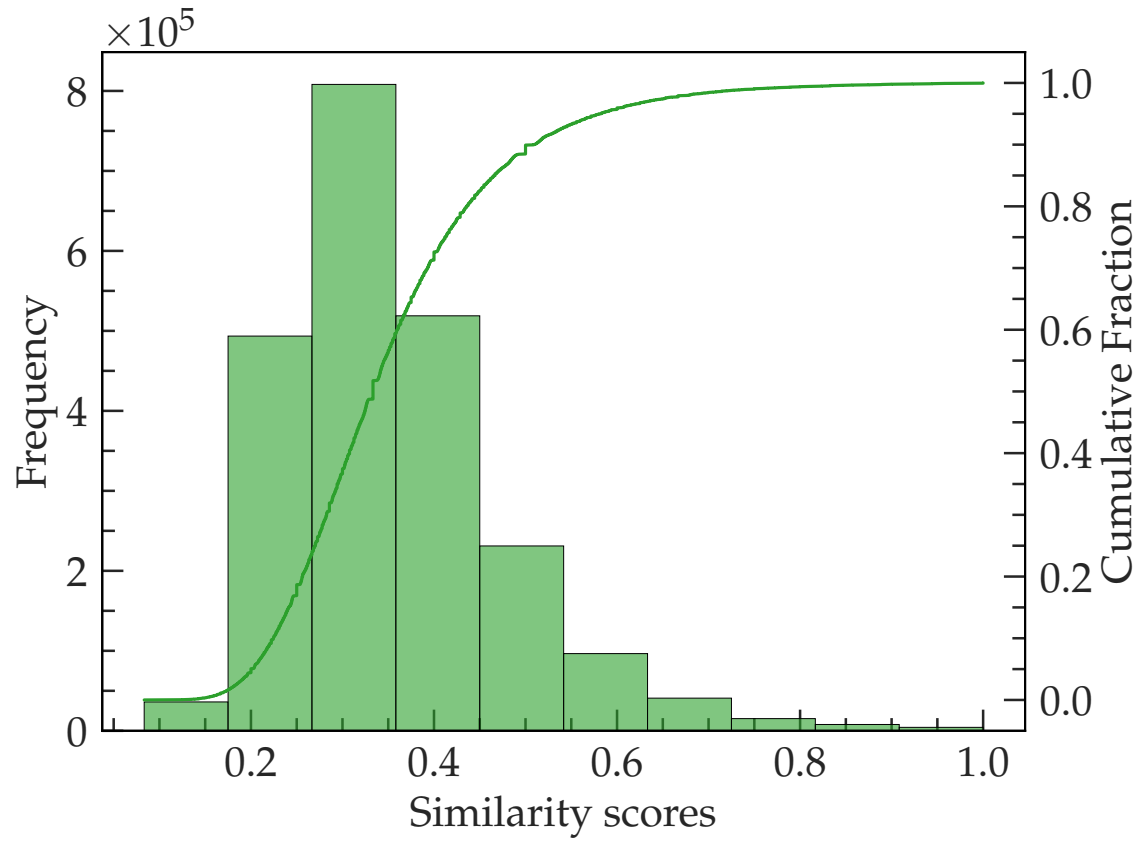

(e)

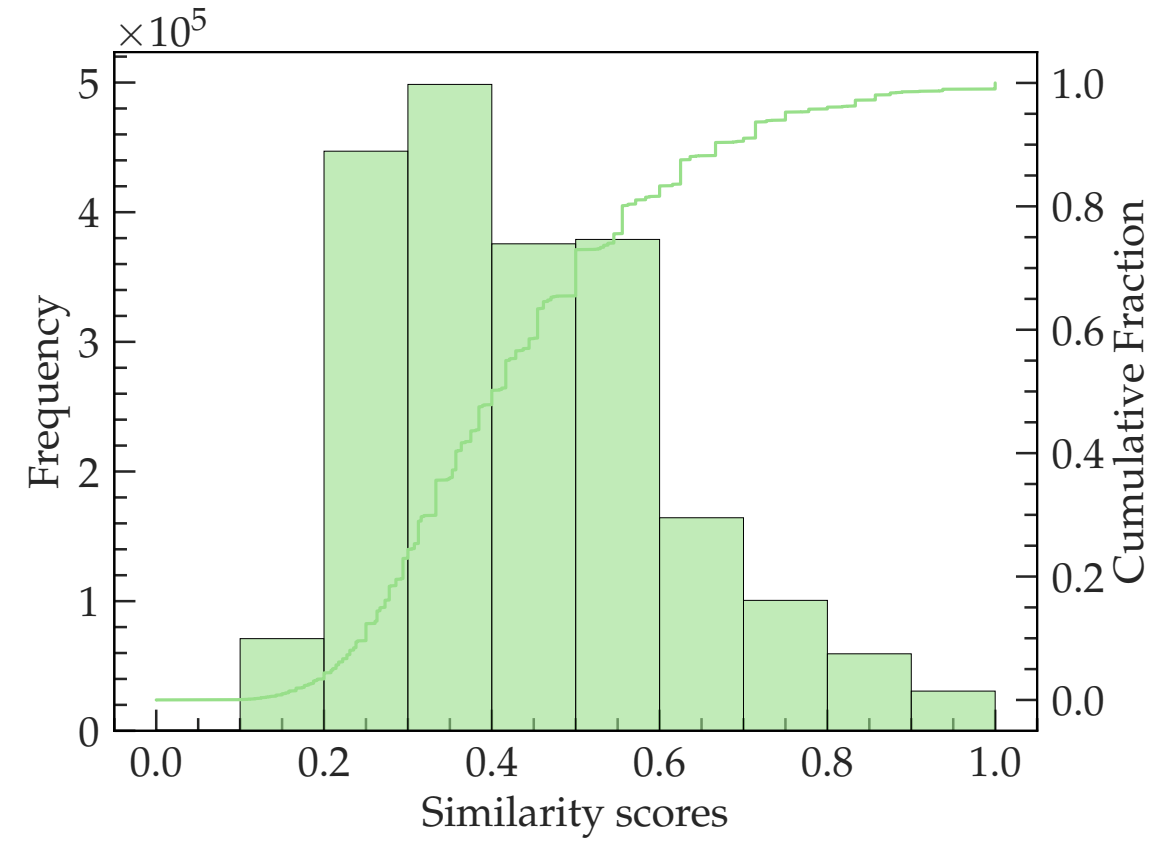

(f)

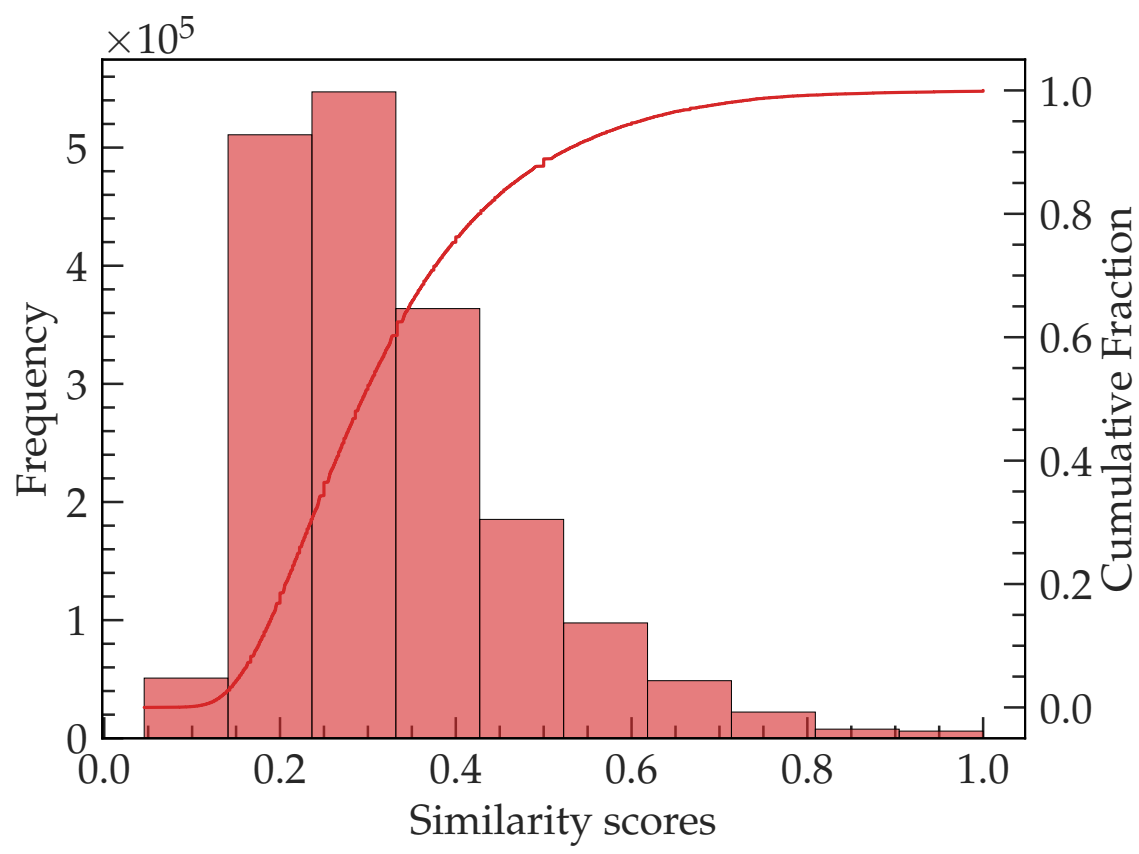

(g)

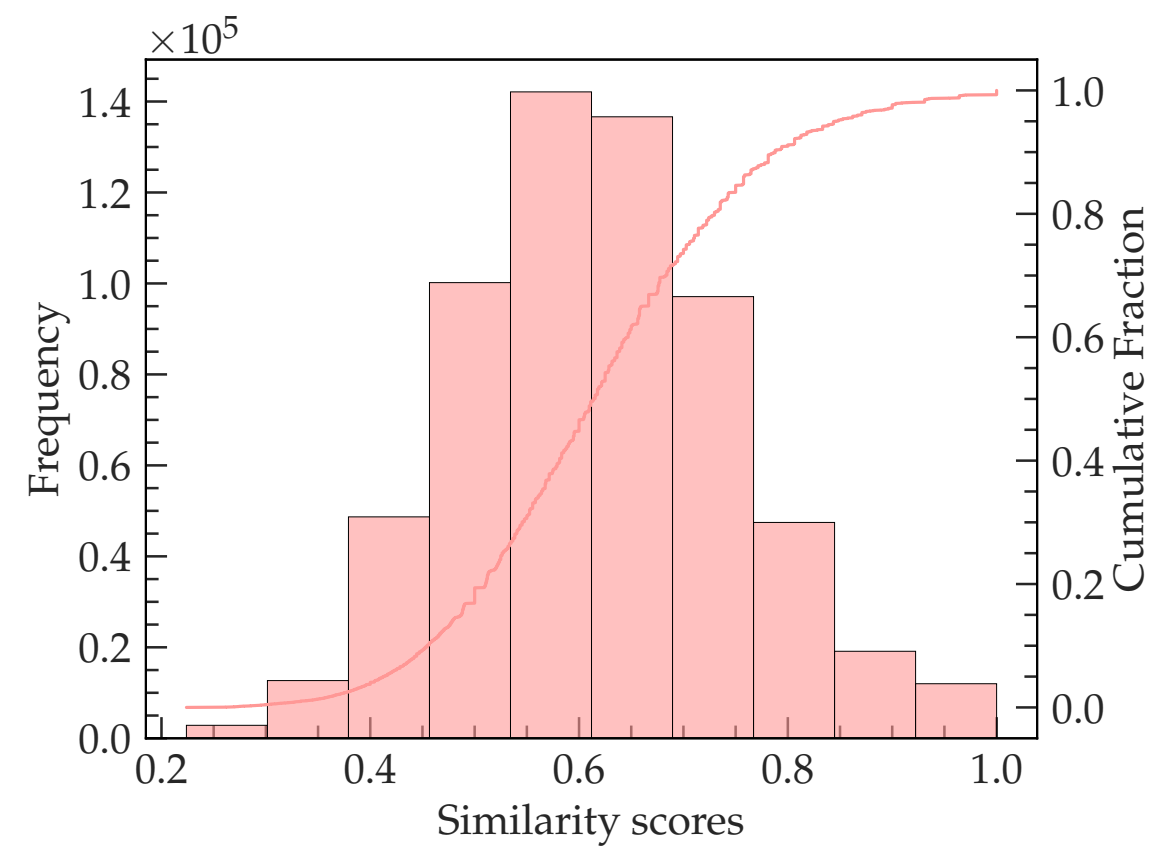

(h)

Figure S13: (continued)

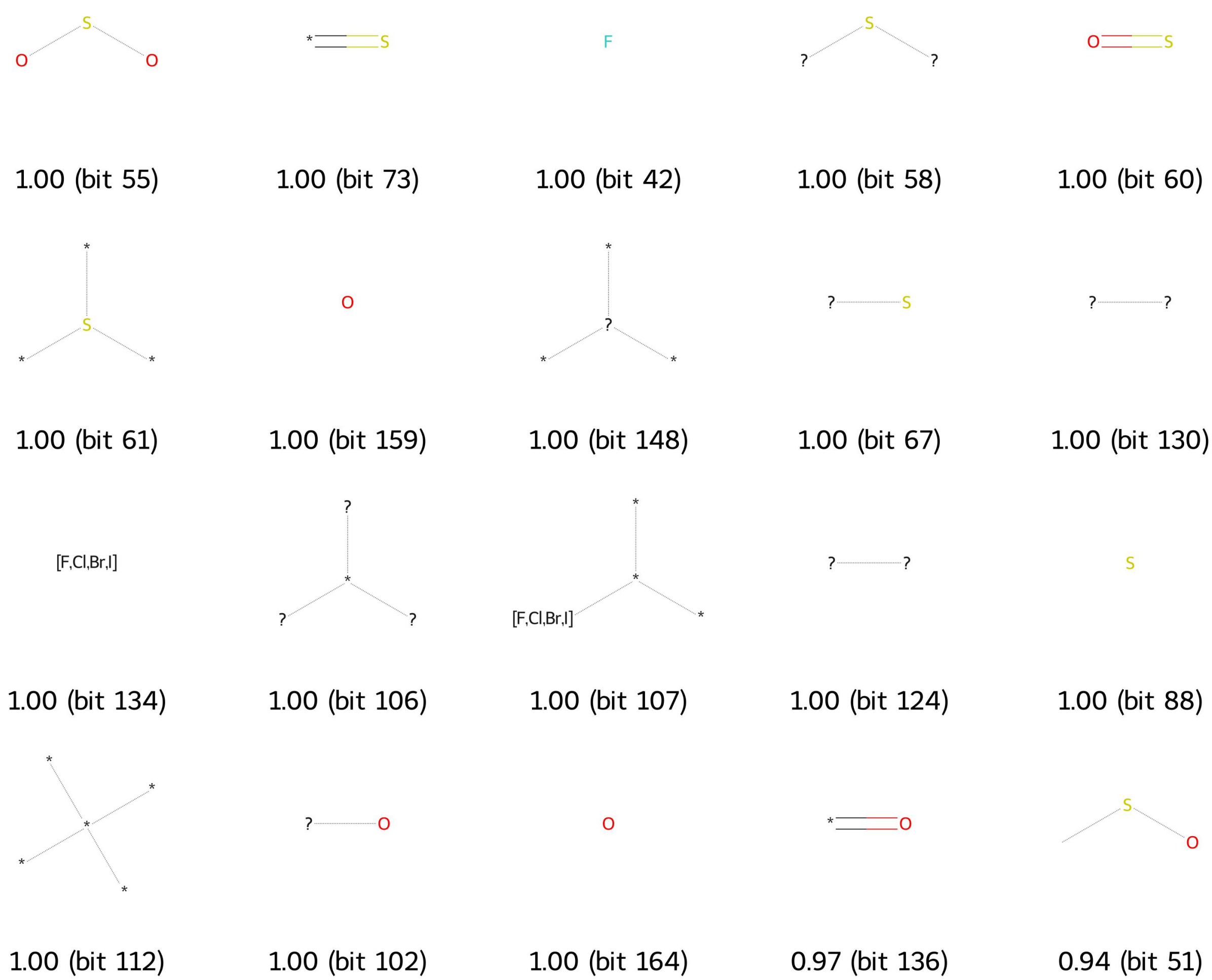

(a)

Figure S14: Top 20 most common substructures based on the MACCS keys contained in (a) MDS group 1, (b) MDS group 2, (c) MDS group 3, (d) MDS group 4, (e) MDS group 5 from Table 2 of the main text. The numbers (outside the bracket) depict the fraction of PFAS structures in the group having the specified substructure, while the bit number in the bracket corresponds to the bit that encodes the substructure based on the MACCS keys.

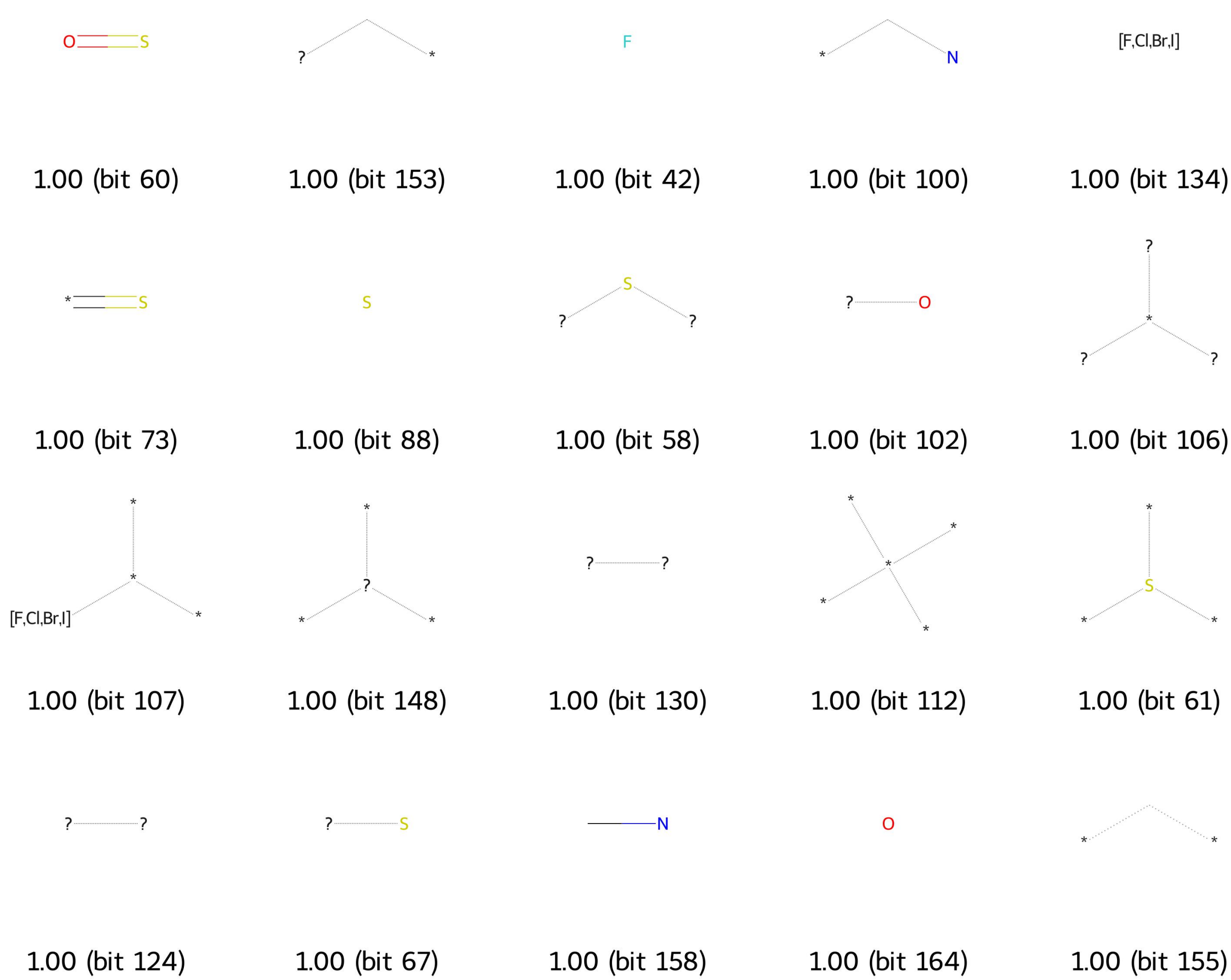

(b)

Figure S14: (continued)

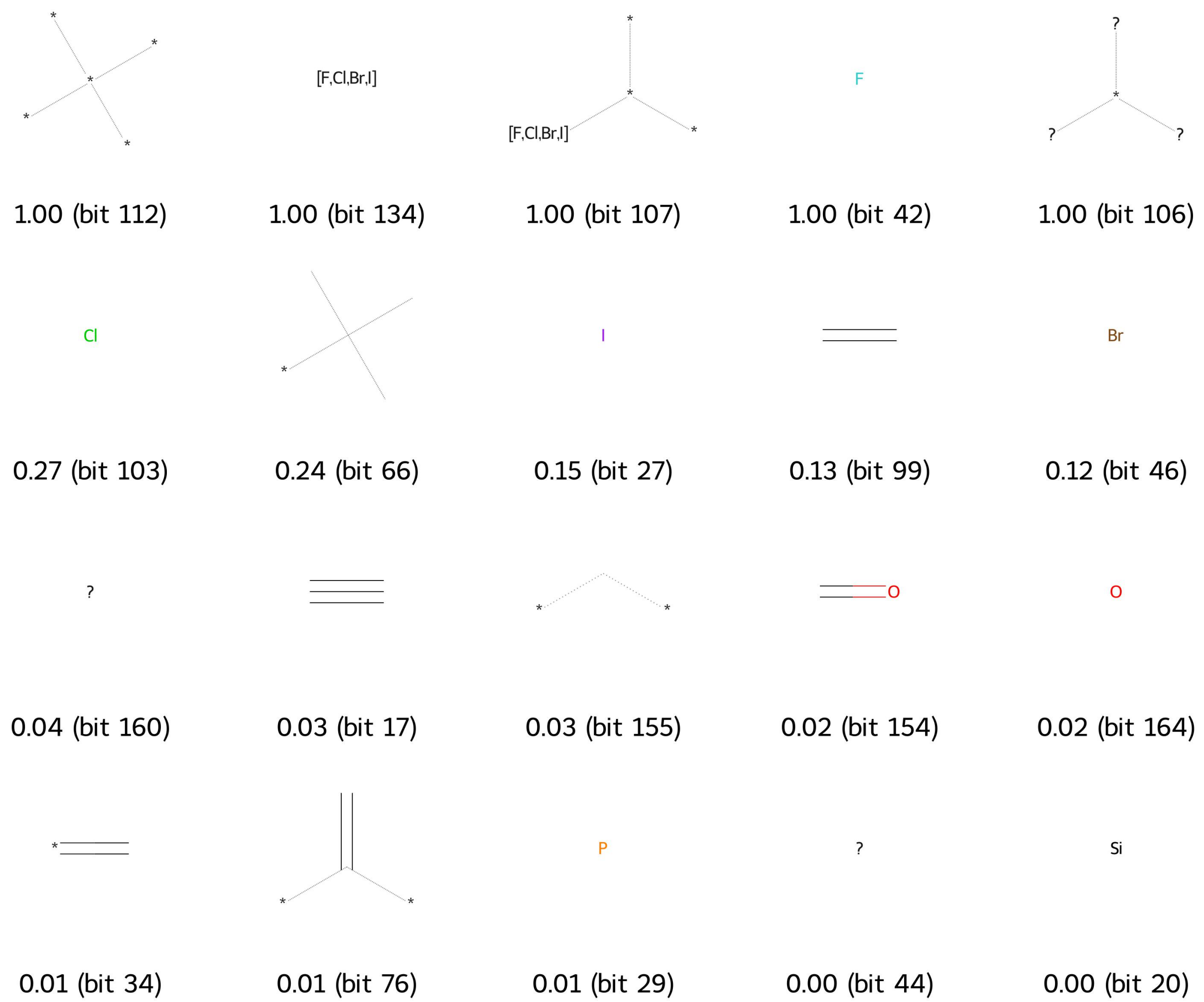

(c)

Figure S14: (continued)

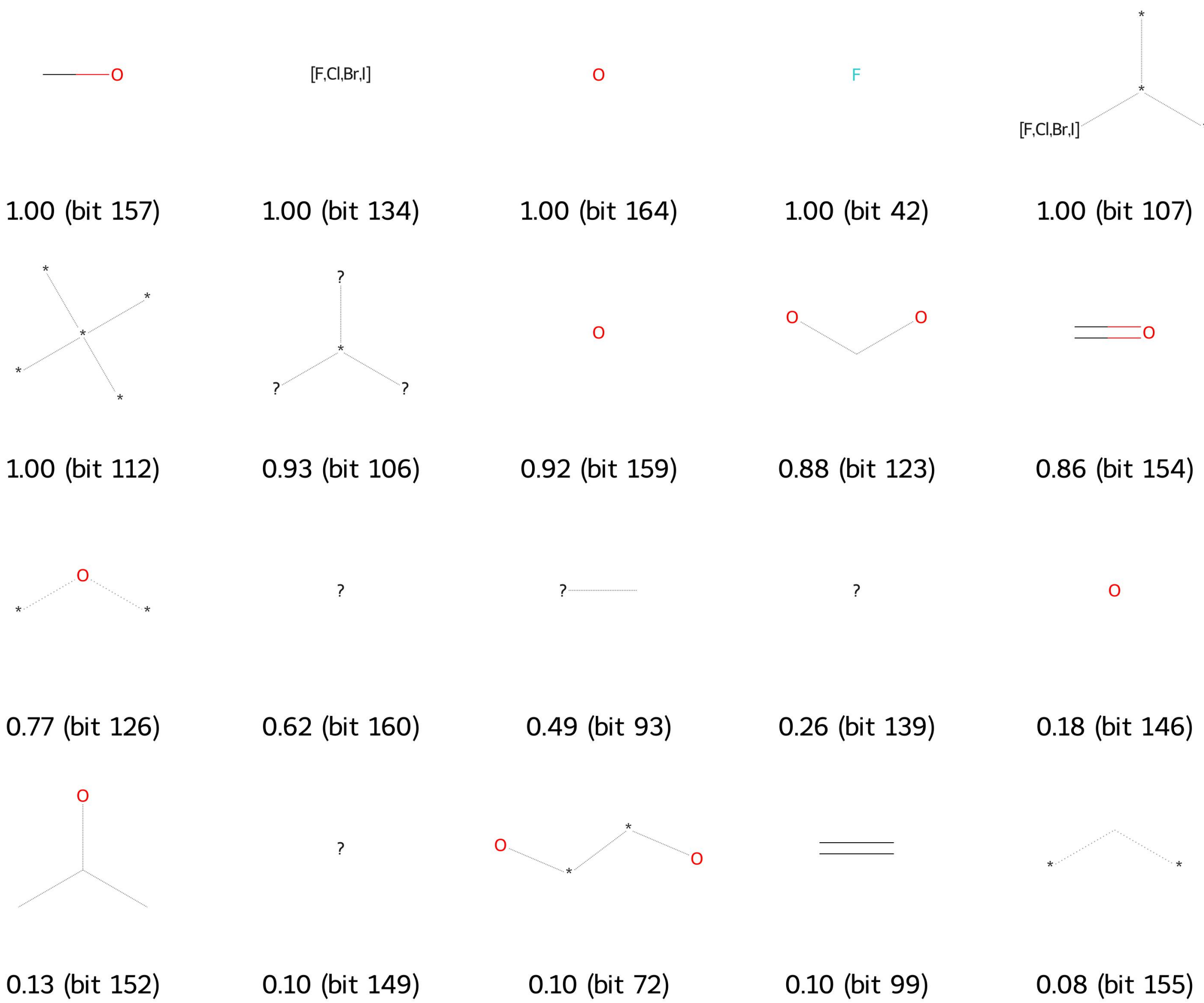

(d)

Figure S14: (continued)

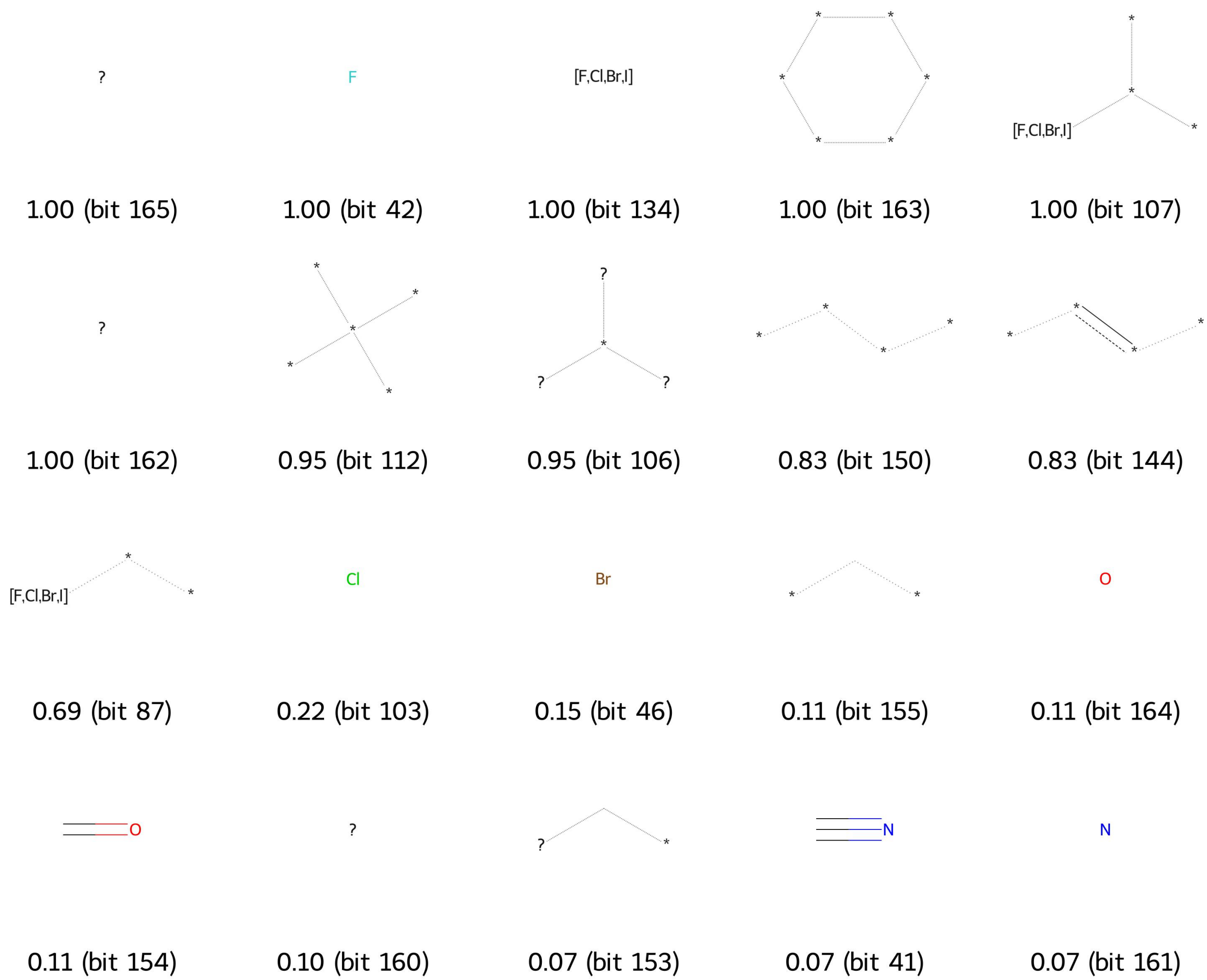

(e)

Figure S14: (continued)

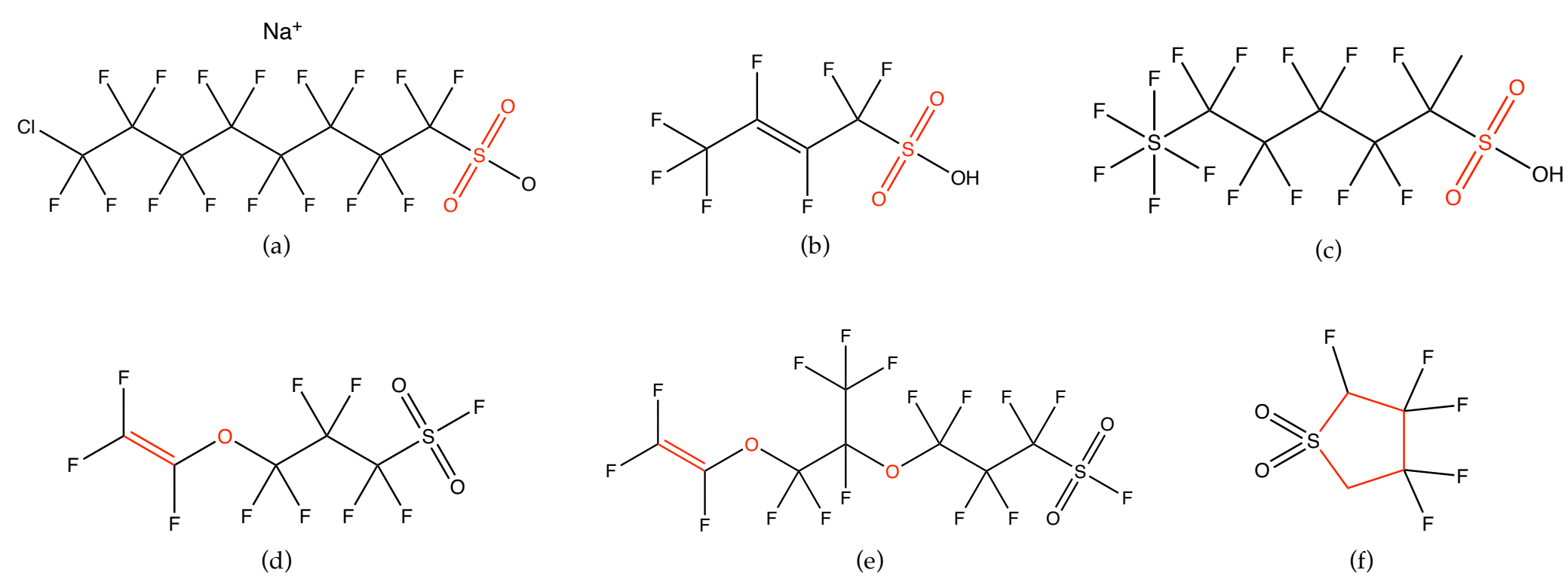

Figure S15: Comparison of representative PFAS identified by the graph-theoretic and t-SNE clustering approaches. Panels (a–c) show representative PFAS that are common to both Group 1 identified by the MDS framework (Table 2, main text) and Cluster 8 obtained from the t-SNE approach (Table S1) resulting from their shared sulfonic groups highlighted in red. Panels (d–f) show PFAS assigned to Cluster 8 by the t-SNE approach but excluded from Group 1 by the MDS framework because of additional distinguishing structural features highlighted in red. These structures are: (a) Sodium 8-chloroperfluoro-1-octanesulfonate, (b) Perfluoro-2-butenesulfonic acid, (c) 5-(Pentafluorosulfanyl)perfluoropentyl sulfonic acid, (d) Perfluoro-3-ethenyloxy-1-propanesulfonyl fluoride, (e) Perfluoro(3-[1-(ethenyloxy)propan-2-yl]oxypropane-1-sulfonyl) fluoride, and (f) 2,3,3,4,4-Pentafluorothiolane 1,1-dioxide.

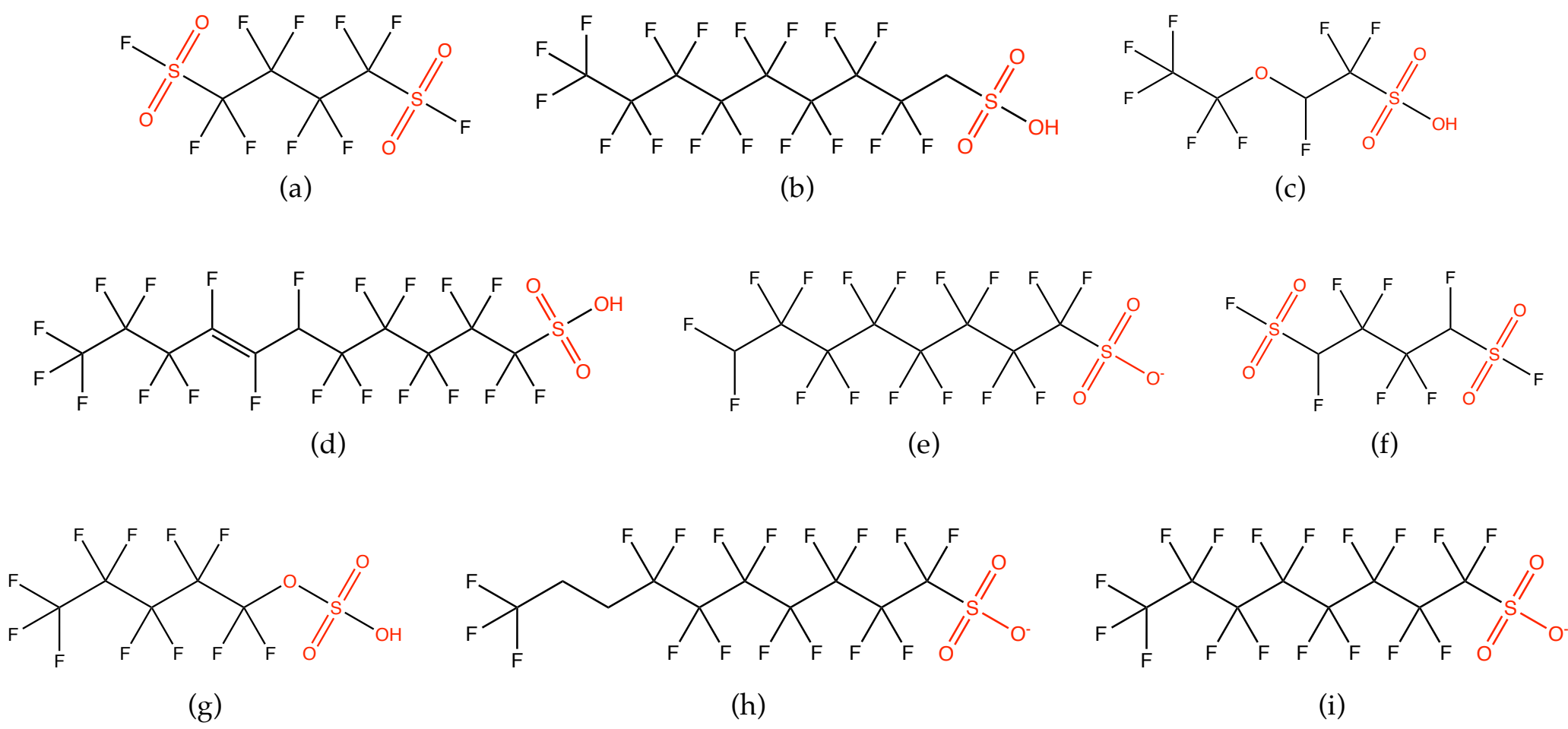

Figure S16: Example of PFAS structures found in PFAS Group 1 represented by Methylsulfenyl trifluoromethanesulfonate obtained via the MDS approach (see Table 2 of the main text). This group contains PFAS structures exclusively with the sulfonate structural motif. These structures are: (a) Perfluorobutane-1,4-disulfonyl difluoride, (b) Perfluorooctyl)methane-1-sulfonic acid, (c) 1,1,2-Trifluoro-2-(1,1,2,2,2-pentafluoroethoxy)ethanesulfonic acid, (d) (E)-5-((Perfluoropent-1-enyl)fluoromethyl)perfluoropentane-1-sulfonic acid, (e) 1,1,2,2,3,3,4,4,5,5,6,6,7,7,8,8-Hexadecafluoro-1-octanesulfonic acid, (f) 1,2,2,3,3,4-Hexafluorobutane-1,4-disulfonyl difluoride, (g) Perfluoropentyl hydrogen sulfate, (h) 7-(3,3,3-Trifluoropropyl)perfluoroheptylsulfonate, and (i) Perfluorooctanesulfonate. The highlighted (in red) substructures show the common functional group within this PFAS group.

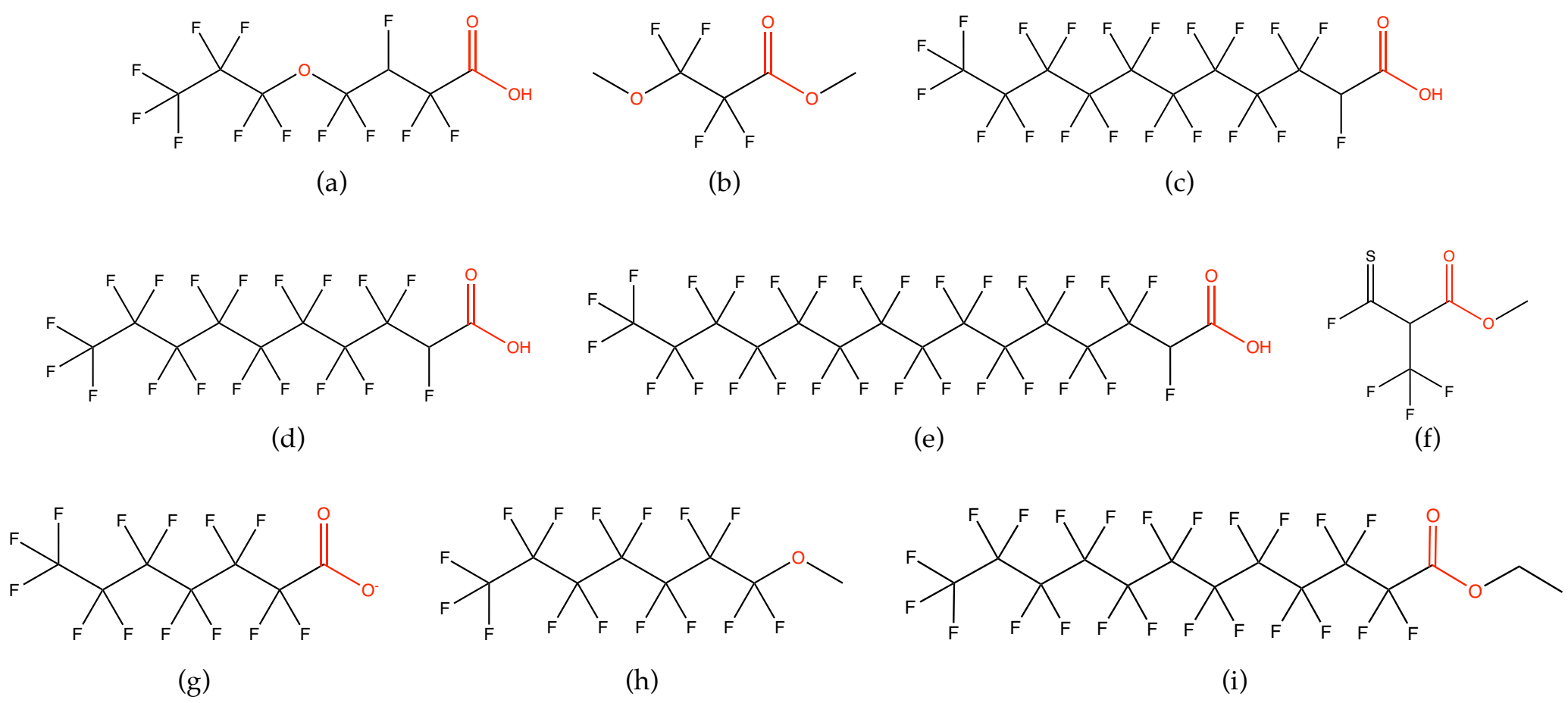

Figure S17: Example of PFAS structures found in PFAS Group 4 represented by Methyl perfluoroheptanoate obtained via the MDS approach (see Table 2 of the main text). This group contains PFAS structures exclusively with the carboxylate structural motif. These structures are: (a) 2,2,3,4,4-Pentafluoro-4-(1,1,2,2,3,3,3-heptafluoropropoxy)butanoic acid, (b) Methyl 2,2,3,3-tetrafluoro-3-methoxypropionate, (c) 11H-Perfluoroundecanoic acid, (d) 2-Hydrido-perfluorodecanoic acid, (e) 2-Hydrido-perfluoropentadecanoic acid, (f) Methyl 3,3,3-trifluoro-2-(fluorocarbonothioyl)propanoate, (g) Perfluoroheptanoate, (h) Methyl perfluoroheptyl ether, and (i) Ethyl perfluorododecanoate. The highlighted (in red) substructures show the common functional group within this PFAS group.

Text S9: Identifying the MDS via the unweighted MDS solution approach using the Morgan fingerprint molecular representation approach

As stated in the main text, the proposed graph-theoretic framework can be applied with different molecular representations. However, the choice of representation influences the resulting graph structure and, consequently, the MDS solution. Here, we solve the MDS problem using Morgan (ECFP) fingerprints,[22] with the results presented in Table S2.

Compared with the MDS results obtained using MACCS keys (Table 1 of the main text), the Morgan fingerprint representation produces fewer edges and, consequently, more isolated nodes across all similarity thresholds. This observation is consistent with the greater structural discrimination of Morgan fingerprints, which generally yield lower pairwise similarity values than MACCS keys. Accordingly, the resulting MDS sizes are substantially larger. For example, at $\epsilon \geq 0.7$, 8,823 PFAS are required to represent the entire chemical space of 14,722 PFAS, corresponding to 60% of the chemical space, compared with 2,555 PFAS (17%) using MACCS keys at the same similarity threshold.

The lower graph density obtained with Morgan fingerprints also resulted in optimality gaps approaching zero across the evaluated similarity thresholds, indicating that optimal MDS solutions were obtained. Notably, although Morgan fingerprints provide greater structural discrimination and can distinguish some molecules differing only in carbon-chain length (see Figure S18), they did not distinguish perfluorotridecanedioic acid and perfluorododecanedioic acid, which differ by a single carbon atom. These results highlight that the choice of molecular representation should be guided by the application and the level of structural detail required to define molecular similarity.

Lastly, Table S3 presents the representative PFAS of the top five largest groups obtained using the Morgan fingerprint representation. We observe that in comparison to Table 2 of the main text, different representative structures are identified. This observation is largely due to the different graph shape formed using this representation.

Table S2: Summary of results obtained from applying the unweighted MDS solution approaches to graphs constructed at varying similarity thresholds (ϵ) using the Morgan Fingerprint (radius = 2) molecular representation, evaluated under a 300-second time limit. The total number of nodes V is the total number of PFAS structures (14,722). Here, $|\mathcal{S}_H|$, $|\mathcal{S}_{IP}|$, and $|\mathcal{S}_{HIP}|$ denote the sizes of the minimum dominating sets (MDS) obtained using the heuristic (H), integer programming (IP), and hybrid heuristic–IP (HIP) approaches, respectively. The MIP gap represents the relative difference between the best-known feasible solution and the optimal (or best bound) solution.

Similarity Threshold (ϵ)	No. of Edges	$\|S_H\|$	$\|S_{IP}\|$	$\|S_{HIP}\|$	MIP gap
0.3	2,867,168	914	543	543	0.03
0.4	731,007	2259	1,768	1,768	0.00
0.5	246,356	4055	3,570	3,570	0.00
0.6	90,077	6573	6,351	6,351	0.00
0.7	43,366	8,905	8,823	8,823	0.00
0.8	26,403	10,298	10,275	10,275	0.00
0.9	18,389	11,266	11,259	11,259	0.00

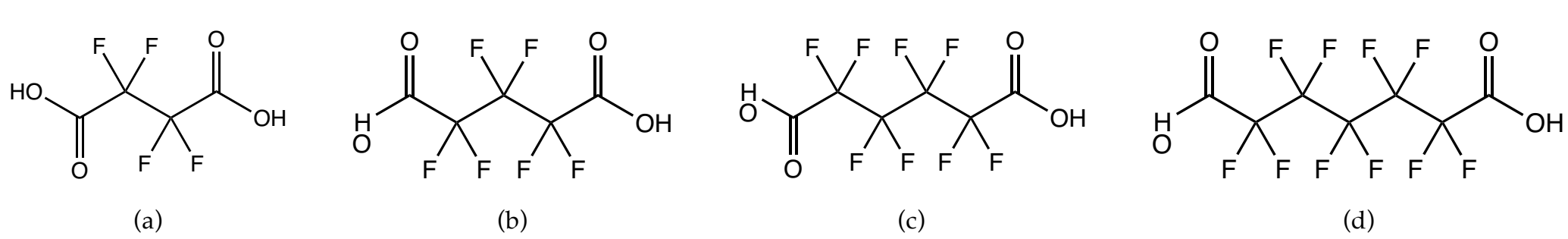

Figure S18: Examples of PFAS with the same functional group (carboxylic acid) but different carbon chain lengths that cannot be differentiated using MACCS key molecular representations. Using MACCS keys, the Tanimoto similarity is 1.0 for every pair among these four PFAS: (a) perfluorobutanedioic acid, (b) perfluoropentanedioic acid, (c) perfluorohexanedioic acid, and (d) perfluoroheptanedioic acid.

Table S3: Top five representative structures of the PFAS chemical space with the most uniquely similar PFAS structures obtained via the Morgan Fingerprint molecular representation. The number in brackets ($|\mathcal{S}_{\mathrm{HIP}}|$) is the number of unique PFAS structures that are at least 70% similar to the representative structure.

Group no. ($|\mathcal{S}_{\mathrm{HIP}}|$)	PFAS Name	Representative Structure
1 (149)	1,4-Bis(perfluoropentyl)butane	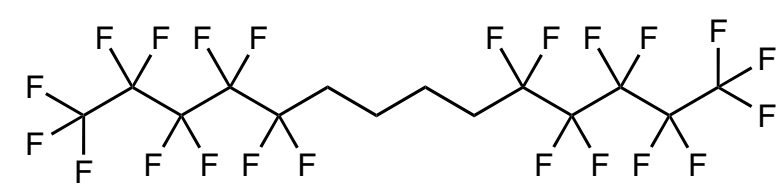
2 (70)	N-(2-Hydroxyethyl)-N,N-dimethyl-3-[(perfluorononyl)sulfonyl]amino-1-propanaminium	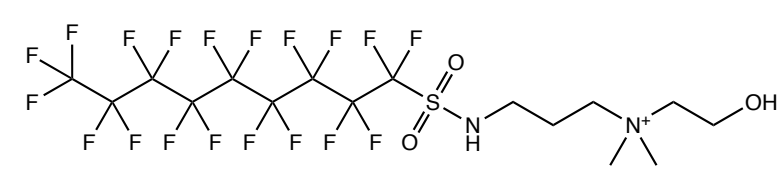
3 (67)	Perfluorobutanoate	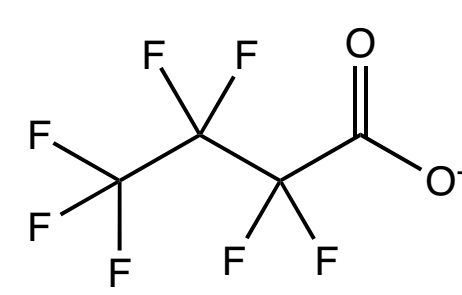
4 (58)	Pentacontafluoro-14,23-diiodohexatriacontane	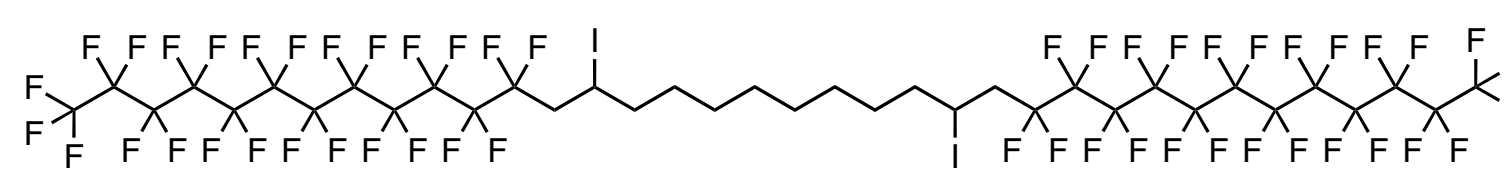
5 (54)	3-[(2-Carboxyethyl)[(perfluoro-1-sulfonyltridecyl)]amino]-N-(carboxymethyl)-N,N-dimethyl- 1-propanoate	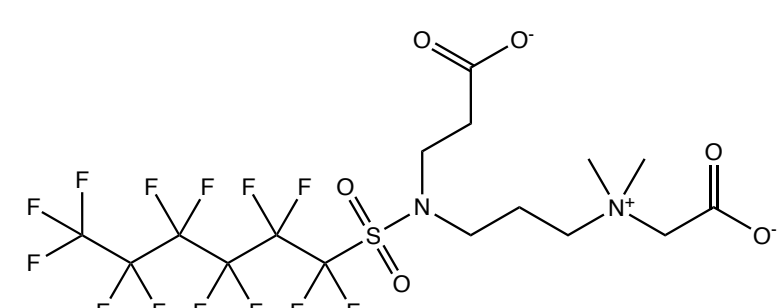

Table S4: Summary of results obtained from applying the weighted MDS formulations to graphs constructed at varying similarity thresholds (ϵ), evaluated under a 300-second time limit. Total number of nodes V is the total number of PFAS structures (14,722). Here, $|\mathcal{S}_{\mathrm{WH}}|$ and $|\mathcal{S}_{\mathrm{WHIP}}|$ denote the sizes of the minimum dominating sets (MDS) obtained using the weighted heuristic-based approximation (WH) and hybrid weighted heuristic–based integer programming (WHIP) approaches, respectively. The MIP gap represents the relative difference between the best-known feasible solution and the optimal (or best bound) solution.

Similarity Threshold (ϵ)	No. of Edges	$\lvert\mathcal{S}_{\mathrm{WH}}\rvert$	$\lvert\mathcal{S}_{\mathrm{WHIP}}\rvert$	MIP gap
0.3	29,966,836	20	20	inf
0.4	13,055,813	65	65	inf
0.5	5,906,127	183	120	0.16
0.6	2,427,930	577	393	0.02
0.7	992,494	1,443	1,148	0.00
0.8	372,039	2,820	2,579	0.00
0.9	119,533	5,207	5,085	0.00

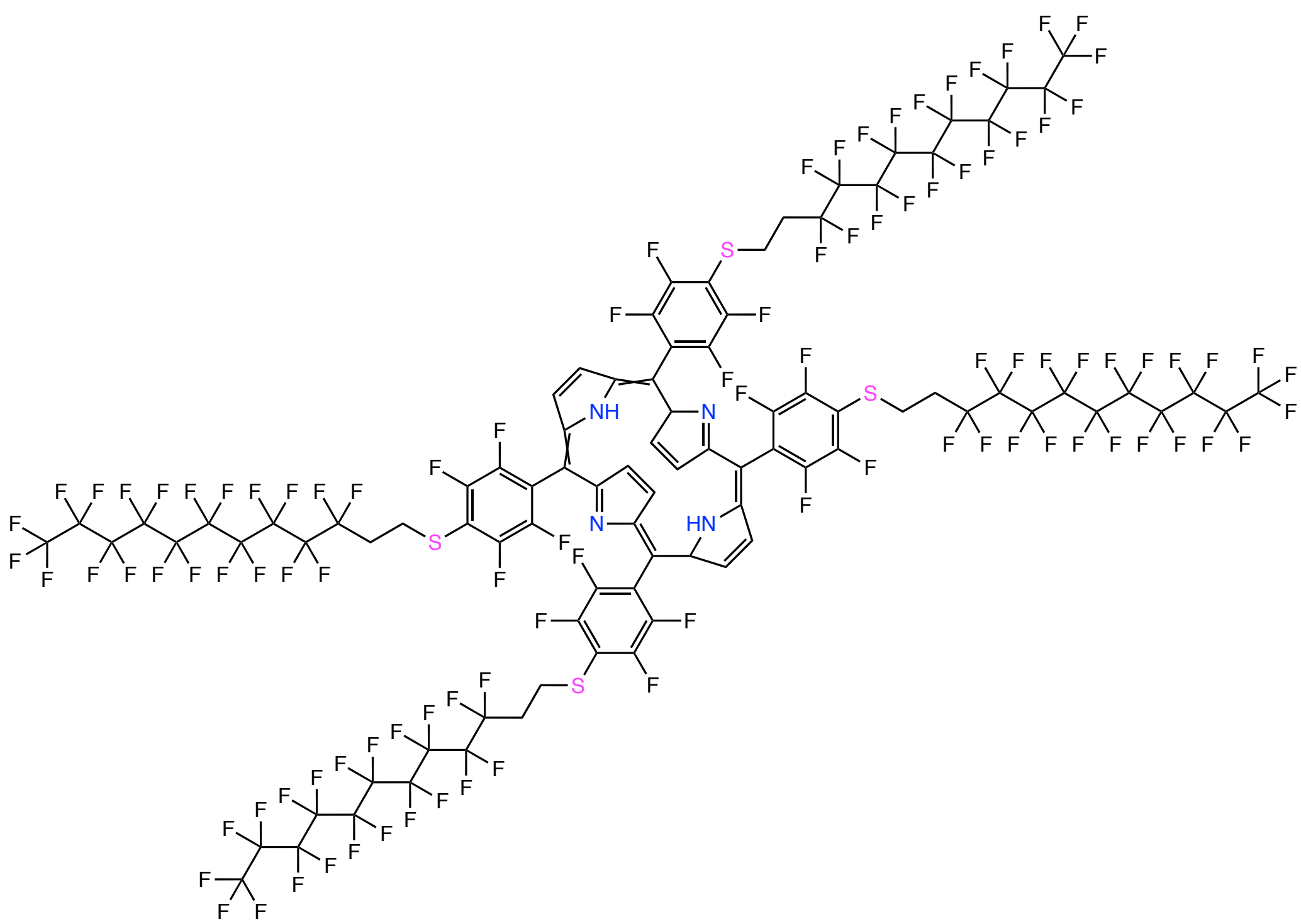

Figure S19: 5,10,15,20-Tetrakis{2,3,5,6-tetrafluoro-4-[(3,3,4,4,5,5,6,6,7,7,8,8,9,9,10,10,11,11,12,12,12-henicosafluorododecyl)sulfanyl]phenyl}porphyrin

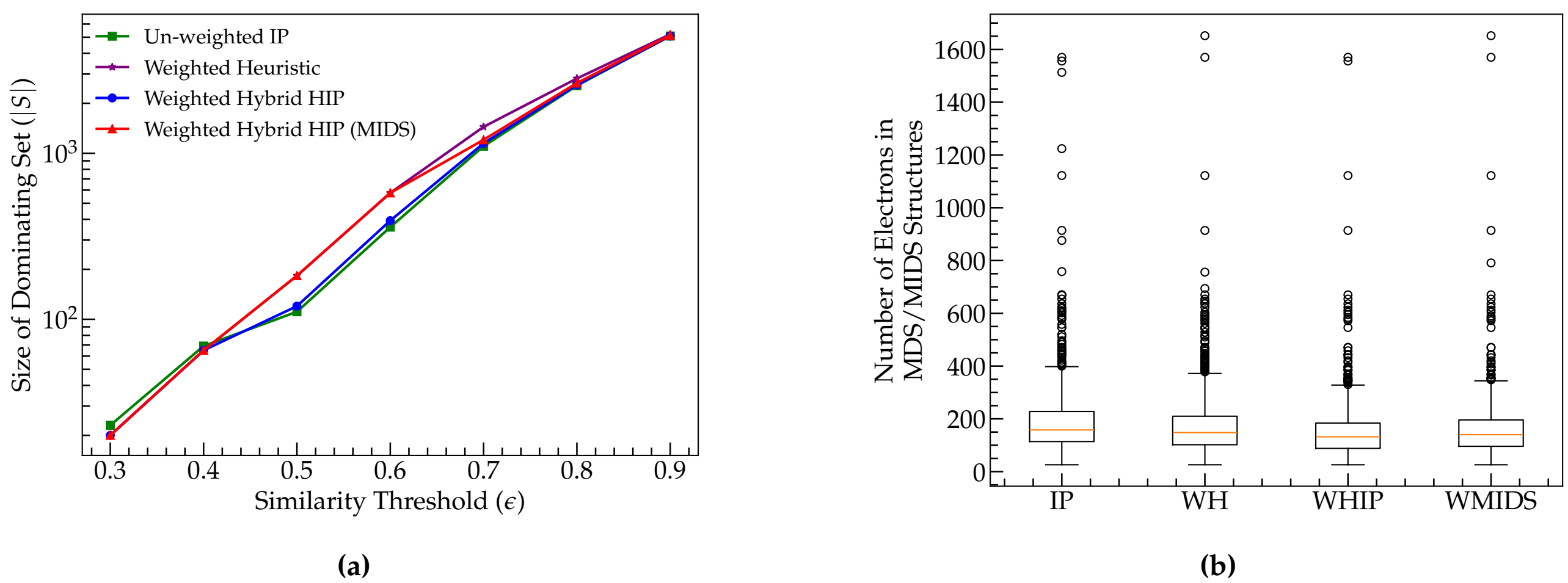

Figure S20: Solutions to the MIDS problem. (a) Comparison between unweighted and weighted minimum dominating set (MDS), and weighted minimum independent dominating set (MIDS) solutions. (b) Box plot showing the distribution of electron counts for PFAS structures included in the MDS and MIDS, obtained using the unweighted integer programming (IP), weighted heuristic (WH), weighted hybrid heuristic-IP (WHIP), and the weighted minimum independent dominating set (WMIDS) approaches. The interquartile ranges for IP, WH, WHIP, and WMIDS are $114.0 - 228.0$, $102.0 - 210.0$, $88.0 - 184.0$, and $96.0 - 196.0$, respectively, while the corresponding average electron counts are 188.6, 173.9, 154.4, and 160.4.

References

(1) Gaines, L. G.; Sinclair, G.; Williams, A. J. *Integrated Environmental Assessment and Management* **2023**, *19*, 1333–1347.

(2) Buck, R. C.; Franklin, J.; Berger, U.; Conder, J. M.; Cousins, I. T.; De Voogt, P.; Jensen, A. A.; Kannan, K.; Mabury, S. A.; Van Leeuwen, S. P. *Integrated environmental assessment and management* **2011**, *7*, 513–541.

(3) Maine Legislature. WATERS AND NAVIGATION: SALE OF CONSUMER PRODUCTS AFFECTING THE ENVIRONMENT (Chapter 16). https://legislature.maine.gov/statutes/38/title38sec1614.html (accessed 01/22/2026).

(4) For Economic Co-operation, O.; Development *Ser Risk Manag* **2021**, *61*, 1–43.

(5) Williams, A. J.; Grulke, C. M.; Edwards, J.; McEachran, A. D.; Mansouri, K.; Baker, N. C.; Patlewicz, G.; Shah, I.; Wambaugh, J. F.; Judson, R. S., et al. *Journal of cheminformatics* **2017**, *9*, 61.

(6) U.S. Environmental Protection Agency. CompTox Chemicals Dashboard. https://comptox.epa.gov/dashboard/chemical-lists/PFASSTRUCT (accessed 11/22/2025).

(7) U.S. Environmental Protection Agency. CompTox Chemicals Dashboard – Version 1. https://comptox.epa.gov/dashboard/chemical-lists/PFASSTRUCTV1 (accessed 11/22/2025).

(8) U.S. Environmental Protection Agency. CompTox Chemicals Dashboard – Version 2. https://comptox.epa.gov/dashboard/chemical-lists/PFASSTRUCTV2 (accessed 11/22/2025).

(9) U.S. Environmental Protection Agency. CompTox Chemicals Dashboard – Version 3. https://comptox.epa.gov/dashboard/chemical-lists/PFASSTRUCTV3 (accessed 11/22/2025).

(10) U.S. Environmental Protection Agency. CompTox Chemicals Dashboard – Version 4. https://comptox.epa.gov/dashboard/chemical-lists/PFASSTRUCTV4 (accessed 11/22/2025).

(11) Williams, A. J.; Gaines, L. G.; Grulke, C. M.; Lowe, C. N.; Sinclair, G. F.; Samano, V.; Thillainadarajah, I.; Meyer, B.; Patlewicz, G.; Richard, A. M. *Frontiers in environmental science* **2022**, *10*, 850019.

(12) U.S. National Center for Biotechnology information. PubChem Compound TOC. https://pubchem.ncbi.nlm.nih.gov/classification (accessed 01/22/2026).

(13) U.S. Environmental Protection Agency. CompTox Chemicals Dashboard – Version 5. https://comptox.epa.gov/dashboard/chemical-lists/PFASSTRUCTV5 (accessed 11/22/2025).

(14) Zhou, P.; Sánchez-Rivera, K. L.; Huber, G. W.; Van Lehn, R. C. *ChemSusChem* **2021**, *14*, 4307–4316.

(15) Zhou, P.; Yu, J.; Sánchez-Rivera, K. L.; Huber, G. W.; Van Lehn, R. C. *Green Chemistry* **2023**, *25*, 4402–4414.

(16) Hinton, G. E.; Roweis, S. *Advances in neural information processing systems* **2002**, *15*.

(17) Van der Maaten, L.; Hinton, G. *Journal of machine learning research* **2008**, *9*.

(18) MacQueen, J. *Proceedings of the 5th Berkeley symposium on mathematical statisticsand probability* **1967**, *1*, 281–297.

(19) Arthur, D.; Vassilvitskii, S. *k-means++: The advantages of careful seeding*; tech. rep.; Stanford, 2006.

(20) Lloyd, S. *IEEE transactions on information theory* **1982**, *28*, 129–137.

(21) Sasmal, S.; Pal, T.; Hocky, G. M.; McCullagh, M. *Journal of chemical theory and computation* **2024**, *20*, 3492–3502.

(22) Morgan, H. *Journal of Chemical Documentation* **1965**, *5*, 107–1.
